# The beneficial effect on cognition of noninvasive brain stimulation intervention in patients with dementia: a network meta-analysis of randomized controlled trials

**DOI:** 10.1186/s13195-023-01164-2

**Published:** 2023-01-25

**Authors:** Ping-Tao Tseng, Yen-Wen Chen, Bing-Yan Zeng, Bing-Syuan Zeng, Chao-Ming Hung, Cheuk-Kwan Sun, Yu-Shian Cheng, Brendon Stubbs, Andre F. Carvalho, Andre R. Brunoni, Kuan-Pin Su, Yu-Kang Tu, Yi-Cheng Wu, Tien-Yu Chen, Pao-Yen Lin, Chih-Sung Liang, Chih-Wei Hsu, Che-Sheng Chu, Mein-Woei Suen, Cheng-Ta Li

**Affiliations:** 1 Prospect Clinic for Otorhinolaryngology & Neurology, Kaohsiung City, Taiwan; 2 Institute of Biomedical Sciences, National Sun Yat-sen University, Kaohsiung, Taiwan; 3 Department of Psychology, College of Medical and Health Science, Asia University, Taichung, Taiwan; 4 Division of Community & Rehabilitation Psychiatry, Department of Psychiatry, Taipei Veterans General Hospital, No. 201, Sec. 2, Shipai Road, Beitou District, Taipei City, 11267 Taiwan; 5 Institute of Precision Medicine, National Sun Yat-sen University, Kaohsiung City, Taiwan; 6 Department of Internal Medicine, E-Da Dachang Hospital, I-Shou University, Kaohsiung, Taiwan; 7 Department of Internal Medicine, E-Da Cancer Hospital, I-Shou University, Kaohsiung, Taiwan; 8 Division of General Surgery, Department of Surgery, E-Da Cancer Hospital, I-Shou University, Kaohsiung, Taiwan; 9 School of Medicine, College of Medicine, I-Shou University, Kaohsiung, Taiwan; 10 Department of Emergency Medicine, E-Da Hospital, I-Shou University, Kaohsiung, Taiwan; 11 I-Shou University School of Medicine for International Students, Kaohsiung, Taiwan; 12 Department of Psychiatry, Tsyr-Huey Mental Hospital, Kaohsiung Jen-Ai’s Home, Kaohsiung, Taiwan; 13 Department of Psychological Medicine, Institute of Psychiatry, Psychology and Neuroscience, King’s College London, London, UK; 14 Physiotherapy Department, South London and Maudsley NHS Foundation Trust, London, UK; 15 Faculty of Health, Social Care Medicine and Education, Anglia Ruskin University, Chelmsford, UK; 16 Innovation in Mental and Physical Health and Clinical Treatment (IMPACT) Strategic Research Centre, School of Medicine, Barwon Health, Deakin University, Geelong, VIC Australia; 17 Service of Interdisciplinary Neuromodulation, National Institute of Biomarkers in Psychiatry, Laboratory of Neurosciences (LIM-27), Departamento e Instituto de Psiquiatria, Faculdade de Medicina da USP, São Paulo, Brazil; 18 Departamento de Ciências Médicas, Faculdade de Medicina da USP, São Paulo, Brazil; 19 Mind-Body Interface Laboratory (MBI-Lab), China Medical University and Hospital, Taichung, Taiwan; 20 An-Nan Hospital, China Medical University, Tainan, Taiwan; 21 Institute of Epidemiology & Preventive Medicine, College of Public Health, National Taiwan University, Taipei, Taiwan; 22 Department of Dentistry, National Taiwan University Hospital, Taipei, Taiwan; 23 Department of Sports Medicine, Landseed International Hospital, Taoyuan, Taiwan; 24 Department of Psychiatry, Tri-Service General Hospital, School of Medicine, National Defense Medical Center, Taipei, Taiwan; 25 Institute of Brain Science, National Yang Ming Chiao Tung University, Taipei, 112 Taiwan; 26 Department of Psychiatry, Kaohsiung Chang Gung Memorial Hospital and Chang Gung University College of Medicine, Kaohsiung, Taiwan; 27 Institute for Translational Research in Biomedical Sciences, Kaohsiung Chang Gung Memorial Hospital and Chang Gung University College of Medicine, Kaohsiung, Taiwan; 28 Department of Psychiatry, Beitou Branch, Tri-Service General Hospital, School of Medicine, National Defense Medical Center, Taipei, Taiwan; 29 Graduate Institute of Medical Sciences, National Defense Medical Center, Taipei, Taiwan; 30 Department of Psychiatry, Kaohsiung Veterans General Hospital, Kaohsiung, Taiwan; 31 Center for Geriatric and Gerontology, Kaohsiung Veterans General Hospital, Kaohsiung, Taiwan; 32 Gender Equality Education and Research Center, Asia University, Taichung, Taiwan; 33 Department of Medical Research, Asia University Hospital, Asia University, Taichung, Taiwan; 34 Department of Medical Research, China Medical University Hospital, China Medical University, Taichung, Taiwan; 35 Division of Psychiatry, School of Medicine, National Yang Ming Chiao Tung University, Taipei, Taiwan; 36 Institute of Brain Science and Brain Research Center, School of Medicine, National Yang Ming Chiao Tung University, Taipei, Taiwan; 37 Functional Neuroimaging and Brain Stimulation Lab, Taipei Veterans General Hospital, No. 201, Sec. 2, Shipai Road, Beitou District, Taipei City, 11267 Taiwan

**Keywords:** Cognition, Primary care, Neuropathology, Alzheimer’s disease, Dementia

## Abstract

**Background:**

Dementia [i.e., Alzheimer disease (AD)], the most common neurodegenerative disease, causes profound negative impacts on executive function and quality of life. Available pharmacological treatments often fail to achieve satisfactory outcomes. Noninvasive brain stimulation (NIBS) techniques, which focally modify cortical function and enhance synaptic long-term potentiation, are potentially beneficial for the cognition in patients with AD. The aim of the current network meta-analysis (NMA) was to evaluate the efficacy and safety of different NIBS interventions in patients with AD through NMA.

**Methods:**

Only randomized controlled trials (RCTs) examining NIBS interventions in patients with AD had been included. All NMA procedures were performed under the frequentist model. The primary and secondary outcomes were changes in cognitive function and quality of life, respectively.

**Results:**

Nineteen RCTs (639 participants) were included. The mean treatment and follow-up durations were 5.7 and 10.5 weeks, respectively. The combination of cathodal tDCS of the left dorsolateral prefrontal cortex and anodal tDCS over the right supraorbital region (c-tDCS-F3 + a-tDCS-Fp2) was associated with a significant beneficial effect on cognition compared with sham controls (standardized mean difference=2.43, 95% confidence interval=0.61–4.26, *n*=12 and 11). It was also associated with the greatest beneficial effect on cognition among all the investigated NIBS approaches. All the methods were well tolerated with regard to the safety profile, as reflected in the rates of adverse events or local discomfort, as well as acceptability, as indicated by dropout rate.

**Conclusions:**

The present findings provide evidence of the benefits of NIBS, especially tDCS, for beneficial effect on cognition in patients with AD. However, because of few studies included, this effect was not replicated yet in the other studies. Therefore, future larger-scale and longer follow-up duration RCTs should be warranted.

**Trial registration:**

PROSPERO CRD42020209516. The current study had been approved by the Institutional Review Board of the Tri-Service General Hospital, National Defense Medical Center (TSGHIRB No. B-109-29).

**Supplementary Information:**

The online version contains supplementary material available at 10.1186/s13195-023-01164-2.

## Introduction

Affecting 46.8 million people globally, the dementia [i.e., Alzheimer disease (AD)] is the most common neurodegenerative disease characterized by progressive cognitive decline [1, 2]. In individuals aged between 65 and 95 years, its incidence doubles approximately every 5 years [3]. AD causes profound negative impacts on executive function and quality of life. Patients also experience memory impairment, behavioral disturbance, and insomnia [4]. Therefore, the amelioration of cognitive decline in the early stages of the disease is regarded as essential in AD management. The efficacy of AD medications, most of which are oral, do not provide satisfactory results [5, 6].

The unsatisfactory efficacy of AD leaves room for alternative treatments. Noninvasive brain stimulation (NIBS) interventions, which are based on the theory of alterations in synaptic function and neuroplasticity [7, 8] and target specific brain regions, have attracted increasing scholarly attention. NIBS techniques include repetitive transcranial magnetic stimulation (rTMS) [9] and transcranial electrical stimulation such as transcranial direct current stimulation (tDCS) [10]. According to the frequency applied, rTMS can induce different changes in brain activity. For example, high-frequency rTMS (HF-rTMS) induces higher brain activity, whereas low-frequency rTMS (LF-rTMS) suppresses cerebral cortex activity [11]. Studies have indicated that anodal [12, 13] and cathodal [10] tDCS exert stimulate and suppress activity in the targeted cortices, respectively. However, whether suppression or enhancement associated with the polarity of tDCS remains under debate [14, 15]. In AD management, rTMS or tDCS of specific cortical regions constitute promising NIBS techniques with regard to their enhancing [10] and neuroplasticity-related effects [16], as well as their effects on neurotransmitter modulation [17, 18].

Conventional pairwise meta-analyses have indicated the substantial efficacy of tDCS [19] and rTMS [20, 21] compared with that of sham treatment in improving cognitive function in patients with AD. In 2020, a recent network meta-analysis (NMA) comparing the efficacy of different NIBS techniques in improving cognitive function in patients with mild cognitive impairment (MCI) or AD suggested that rTMS was superior to tDCS and that patients with AD responded better to rTMS and tDCS than those with MCI did [22]. We identified 3 major limitations from this study. First, it did not address the regions that were stimulated in the interventions; for instance, rTMS over the left dorsolateral prefrontal cortex (DLPFC) and over multiple brain regions were considered to be the same technique. Second, heterogeneity risk was increased because patients with MCI were included along with patients with AD. Third, the potentially synergistic effects or priming effect [23] of stimulations over different cortex within the same session were not addressed. Similar limitations are observable in a past pairwise meta-analyses [24]. Specifically, methodological limitations meant that different NIBS interventions were not effectively compared. Conventional pairwise meta-analyses cannot provide information regarding the relative efficacy of interventions that have not been directly compared in head-to-head RCTs, which constitutes an essential indicator of interventions’ therapeutic value. The risk was heterogeneity among studies which was also increased in other meta-analyses that included RCTs of not only patients with AD but also those with MCI [19, 20, 25].

Given this background, an NMA including only patients with AD that addresses variations in the targeted brain regions in NIBS interventions enables the estimation of comparative efficacy or risk and the understanding of the relative merits of different interventions. To the best of our knowledge, no NMAs on this subject have been performed. Therefore, we conducted a systematic review and NMA to compare the efficacy and safety of various central NIBS interventions in cognitive function in patients with AD.

## Methods

### General aspects

The NMA was performed according to the guidelines of the Preferred Reporting Items for Systematic Reviews and Meta-Analyses statement 2020 guideline (eTable 1) [26] and AMSTAR2 (a measurement tool to assess systematic reviews) guideline [27]. The current study had been approval by the Institutional Review Board of the Tri-Service General Hospital, National Defense Medical Center (TSGHIRB No. B-109-29).

### Search strategy and selection criteria

The systematic review involved searching the PubMed, Embase, ClinicalKey, Cochrane CENTRAL, ProQuest, ScienceDirect, Web of Science, and ClinicalTrials.gov databases from their inception to September 15, 2020, and final update at November 26, 2020 (the keyword used in each database had been listed in eTable 2). No language restrictions were applied. The reference lists of review articles and pairwise meta-analyses [19–21, 24, 25, 28–37] were manually searched for additional potentially eligible articles.

### Eligibility criteria

The PICO of the current study was (1) Patient or Problem: patients with AD; (2) Intervention: central NIBS method; (3) Comparator: Sham-control; and (4) Outcome: the changes of overall cognition. To be specific, the diagnosis of AD could be dementia due to probable or possible AD. We recognized that the definite diagnosis of AD would be clinically difficult because to obtain the biomarker evidence would be difficult in the most situation. We did not restrict to the diagnosis of AD-based biomarker confirmation. Furthermore, we did not set any restriction to the baseline dementia severity.

We included only published RCTs, which were conducted on humans that used either sham or active controls, evaluating changes of overall cognition rather than a single domain of cognition. The central NIBS method used in patients with AD was set as the target in the comparison arms. To reduce the potential risk of heterogeneity, we only included RCTs on patients with AD and not those on patients with MCI. The NIBS methods considered comprised rTMS, tDCS, and theta burst stimulation (TBS). Detailed categorization of the treatment arms is presented in the node definition section.

In sum, the exclusion criteria were as follows: (1) not being an RCT, (2) not reporting the target outcomes (defined later), (3) not being related to the NIBS methods, and (4) not including patients with AD. In cases of duplicated usage of data (i.e., different articles using the same sample sources), we included only the article that had the most informative and largest sample source.

### Data extraction

Two authors (YW Chen and BS Zeng) independently screened the studies, extracted the relevant information from the manuscripts, and evaluated the risk of bias in the included studies. Data was extracted on September 20, 2020. Any disagreements were resolved through discussion with the corresponding author (PT Tseng). If manuscript data were not available, we contacted the corresponding authors of coauthors of the publication to obtain the original data. We only extracted data on NIBS techniques, which did not include peripheral stimulation.

### Outcomes

#### Primary outcome

Changes in cognitive function, the primary outcome, could be measured using the Mini-Mental State Examination (MMSE) or the Alzheimer’s Disease Assessment Scale-Cognitive subscale (ADAS-Cog), which are used worldwide to measure cognitive decline in patients with AD following treatment for Alzheimer dementia. When a study provided results on both scales, the MMSE results were prioritized for primary outcome measurement for the following reasons. (1) The MMSE scores had approximately linear relationships with the quality of life scores (association between the Quality of Life Scale and MMSE scores: *r* = .30, *P* < .0001) [38]. (2) The MMSE is widely used and approved to serve as a surrogate for more time-consuming methods for dementia staging such as the Clinical Dementia Rating [39]. (3) The initial MMSE scores were significant for determining the time to clinically meaningful decline during longitudinal follow-up [40]. Similarly, the MMSE total score can serve as an index of disease progression and cognitive decline in patients with AD [41]. (4) The MMSE is suitable for evaluating patients with varying severities of AD; by contrast, the ADAS-Cog is recommended only for patients with MMSE scores of ≥14 [42]. Whenever MMSE scores were not available, we have chosen ADAS-Cog scores. Notably, previous RCTs have found that patients with AD receiving sham controls exhibited improvement in cognitive function but returned to baseline in a longer follow-up (2–6 mo) [10, 43]. Therefore, to reduce the risk of these potential time effects, we extracted the data from the “final follow-up assessment.”

#### Secondary outcome, safety profile, and intervention acceptability

The secondary outcome was the changes in quality of life. The safety profile was calculated using the rate of any adverse event and the rate of any local discomfort, including headache, itching, swelling, or local erythematous changes. Intervention acceptability was calculated using the dropout rate, which was defined as the percentage of patients withdrawing their participation before the end of study period for any reason.

### Node definition

Because the central NIBS methods varied widely among the studies, we categorized them into 2 major subgroups: (1) rTMS modalities, namely HF-rTMS (≥5 Hz) and LF-rTMS (<5 Hz), and (2) tDCS modalities, which were particularly categorized according to the anodal or cathodal placement position (e.g., a-tDCS over F3 or c-tDCS over F3). We further categorized the treatment arms on the basis of the stimulation position according to the electroencephalogram brain map. The nomenclature of the node definition was defined according to our previous four NMAs of NIBS studies in other different neuropsychiatric diseases [23, 44–46].

### Cochrane risk-of-bias tool

Two independent authors (YW Chen and BS Zeng) evaluated the risk of bias (interrater reliability =.85) for each domain using the Cochrane risk-of-bias tool [47]. The studies were further categorized according to overall risk of bias.

### Statistical analysis

The NMA was performed using STATA (version 16.0; StataCorp Statistics/Data Analysis, StataCorp LLC, College Station, TX, USA). We estimated the standardized mean difference (SMD) with a 95% confidence intervals (95%CIs) for the continuous variable (i.e., the primary outcome of changes in cognitive function). In the subgroup analysis based on specific cognitive rating scales (i.e., the MMSE and ADAS-Cog), we estimated the mean differences (MDs) with 95%CIs to provide additional clinical information. For the categorical variables (i.e., the safety profile and acceptability), we estimated efficacy using odds ratios and 95%CIs and applied a 0.5 zero-cell correction during the meta-analysis procedure. However, if both the intervention and control arms of a study contained zeroes, we did not apply this correction procedure because of the risk of increasing the bias but rather excluded such studies from the analysis [48, 49]. We used the frequentist model of NMAs to compare the effect sizes of studies with similar interventions. All comparisons were performed using 2-tailed *t* tests, and differences were considered significant at *P* < .05. Between-study heterogeneity was evaluated using the tau value, the estimated standard deviation of the effect across the studies.

The meta-analysis procedure was mixed comparisons with direct and indirect comparisons made using generalized linear mixed models [50]. Specifically, indirect comparisons were conducted using transitivity, in which the differences between treatments A and B could be calculated from their comparisons with a third treatment C. To compare multiple treatment arms, we combined the direct and indirect evidence from the included studies [51]. The mvmeta command in STATA software [52] was used in the NMA. The restricted maximum likelihood method was used to evaluate the between-study variance [53].

To provide additional information for clinical application, we calculated the relative ranking probabilities of the treatment effects of all treatments for the target outcomes. In brief, we used the surface under the cumulative ranking curve (SUCRA), which indicates the percentage of the mean rank of each treatment relative to an imaginary intervention that is without uncertainty the optimal one [54].

We evaluated the potential within-network inconsistencies between the direct and indirect evidence by using the loop-specific approach, identifying local inconsistencies through the node-splitting method. The design-by-treatment model was used to evaluate global inconsistencies in the NMA [55]. We evaluated the quality of evidence with the Grading of Recommendations Assessment, Development and Evaluation (GRADE) rating tools described previously [56, 57]. Finally, according to the rationale of a previous NMA study [58], we assessed the effectiveness of the sham interventions to justify our assumption of transitivity. Specifically, we determined cognitive changes following sham tDCS and rTMS using Comprehensive Meta-Analysis software (version 3; Biostat, Englewood, NJ, USA).

## Results

**Fig. 1:**
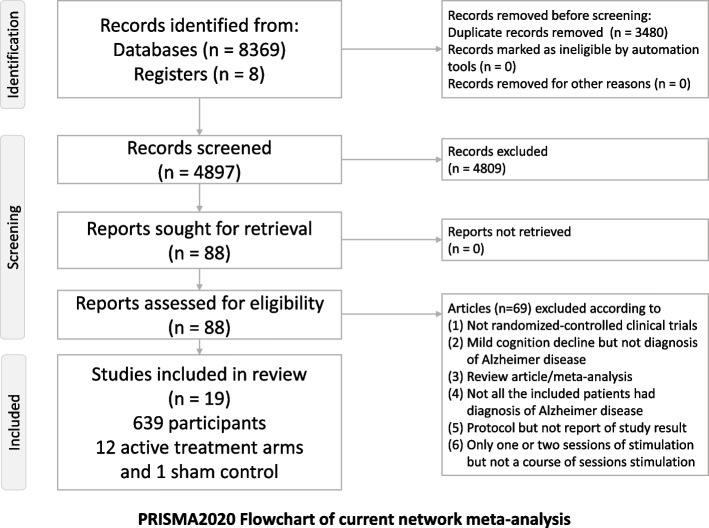
PRISMA2020 flowchart of the current network meta-analysis

**Fig. 2:**
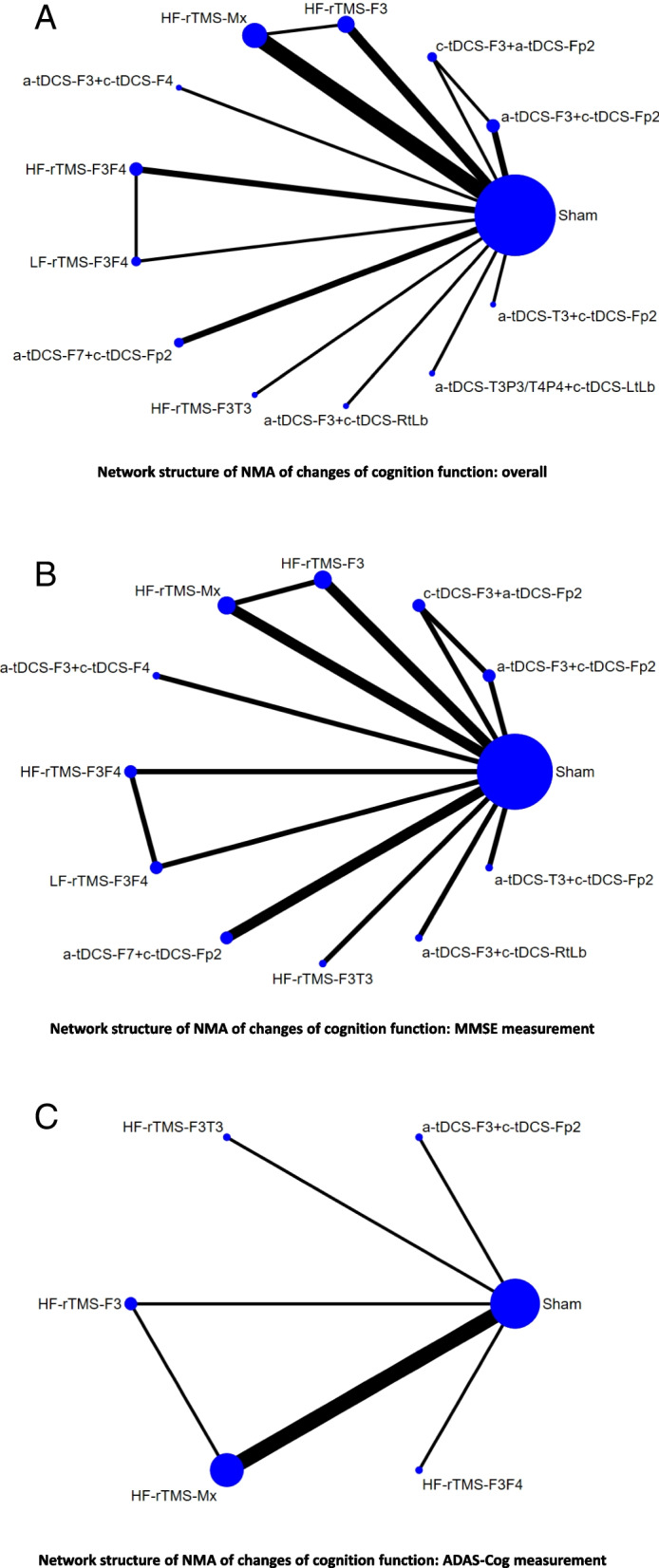
Network structure of the changes of cognitive function. Overall structure of the network meta-analysis. The lines between nodes represent direct comparisons in various trials, and the size of each circle is proportional to the number of participants in each specific treatment. The thickness of the lines is proportional to the number of trials connected to the network

After the initial screening procedure, 88 articles were considered for full-text review (Fig. 1). After 69 were excluded for various reasons (eTable 3), 19 remained for analysis (eTable 4) [2, 9, 10, 43, 59–73]. Figure 2 presents the overall geometric distribution of the treatment arms.

### Characteristics of the included studies

The mean age of the 639 participants was 72.5 years (range = 65.7–80.5 y; interquartile range [IQR] = 69.0–73.6 y), and women constituted 54.7% (range =10.0–83.3%; IQR = 44.1–65.4%). The mean duration of central NIBS treatment was 5.7 weeks (range = 2–32 wk; IQR = 2–6 wk). The corresponding mean follow-up duration was 10.5 weeks (range = 2–32 wk; IQR = 3.8–13.3 wk). The mean baseline MMSE was 19.1 (range = 14.8–22.5; IQR = 15.9–21.5). The most included RCTs allowed participants to use concomitant medication during the interventions [9, 43, 59–64, 67, 68, 70–72]. The investigated NIBS approaches were tDCS [10, 43, 60, 63, 67, 69, 72] and rTMS [2, 9, 59, 61, 62, 64–66, 68, 70, 71, 73]. All the tDCS treatments applied were 2mA in intensity. Although we planned to consider other NIBS techniques (i.e., TBS), no relevant RCTs on patients with AD receiving such therapies were retrieved.

### Primary outcome: changes in cognitive function (based on either MMSE or ADAS-Cog measurement)

**Fig. 3:**
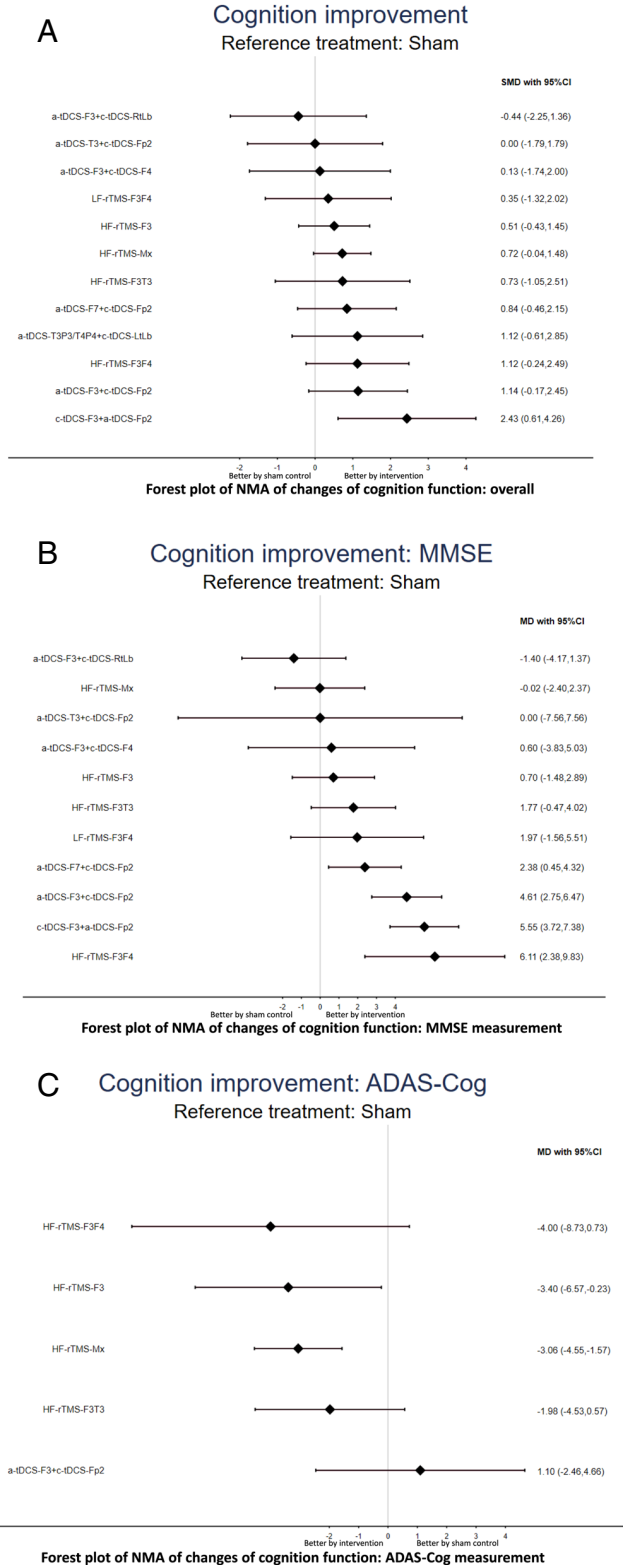
Forest plot of changes of cognitive function. When the effect size (expressed as standardized mean differences) exceeded zero, the specified treatment was associated with greater improvement in cognitive function in patients with Alzheimer disease than in patients receiving sham controls

The NMA revealed that only the cathodal tDCS of the left DLPFC (F3) and anodal tDCS over the right supraorbital region montage (Fp2; c-tDCS-F3 + a-tDCS-Fp2; SMD = 2.43 [95%CIs = 0.61-4.26]) were associated with significant beneficial effect on cognition compared with sham controls (Table 1, Figs. 2A and 3A). The associations between the NIBS methods and the beneficial effect on cognition were ranked according to the SUCRA. In brief, c-tDCS-F3 + a-tDCS-Fp2 was associated with the greatest benefit, followed by high-frequency rTMS over the bilateral DLPFC (HF-rTMS-F3F4; SMD = 1.12 [95%CIs = −0.24 to 2.49]) and anodal tDCS of the left DLPFC and cathodal tDCS over the right supraorbital region (a-tDCS-F3 + c-tDCS-Fp2; SMD = 1.14 [95%CIs = −0.17 to 2.45]; eTable 6A).

**Table 1:** League table of the changes of overall cognition function

c-tDCS-F3 + a-tDCS-Fp2		0.59 (−0.24, 1.42)									*3.50 (2.28, 4.71)	
1.31 (−0.97, 3.59)	HF-rTMS-F3F4							*0.82 (0.08, 1.56)			*1.16 (0.49, 1.83)	
1.30 (−0.42, 3.01)	−0.02 (−1.91, 1.87)	a-tDCS-F3 + c-tDCS-Fp2									1.30 (−1.77, 4.37)	
1.31 (−1.21, 3.83)	0.00 (−2.20, 2.20)	0.02 (−2.15, 2.19)	a-tDCS-T3P3/T4P4 + c-tDCS-LtLb								*1.12 (0.48, 1.76)	
1.59 (−0.65, 3.83)	0.28 (−1.61, 2.17)	0.30 (−1.55, 2.14)	0.28 (−1.89, 2.45)	a-tDCS-F7 + c-tDCS-Fp2							0.81 (−0.07, 1.68)	
1.71 (−0.26, 3.69)	0.40 (−1.16, 1.97)	0.42 (−1.09, 1.93)	0.40 (−1.49, 2.29)	0.12 (−1.39, 1.64)	HF-rTMS-Mx		−0.26 (−1.17, 0.64)				*0.78 (0.15, 1.42)	
1.71 (−0.85, 4.26)	0.40 (−1.85, 2.64)	0.41 (−1.80, 2.62)	0.39 (−2.09, 2.88)	0.12 (−2.10, 2.33)	−0.01 (−1.95, 1.93)	HF-rTMS-F3T3					0.73 (−0.04, 1.50)	
1.93 (−0.13, 3.99)	0.62 (−1.04, 2.27)	0.63 (−0.98, 2.24)	0.62 (−1.35, 2.59)	0.34 (−1.27, 1.95)	0.21 (−0.87, 1.30)	0.22 (−1.79, 2.24)	HF-rTMS-F3				*0.48 (0.04, 0.92)	
2.09 (−0.39, 4.56)	0.78 (−0.90, 2.45)	0.79 (−1.33, 2.91)	0.77 (−1.63, 3.18)	0.50 (−1.62, 2.62)	0.37 (−1.46, 2.20)	0.38 (−2.06, 2.82)	0.16 (−1.76, 2.07)	LF-rTMS-F3F4			0.39 (−0.33, 1.11)	
2.31 (−0.31, 4.92)	1.00 (−1.32, 3.31)	1.01 (−1.27, 3.29)	0.99 (−1.55, 3.54)	0.72 (−1.56, 3.00)	0.59 (−1.42, 2.61)	0.60 (−1.98, 3.18)	0.38 (−1.71, 2.47)	0.22 (−2.28, 2.72)	a-tDCS-F3 + c-tDCS-F4		0.13 (−0.82, 1.08)	
2.43 (−0.12, 4.99)	1.12 (−1.13, 3.37)	1.14 (−1.08, 3.36)	1.12 (−1.37, 3.61)	0.84 (−1.37, 3.06)	0.72 (−1.22, 2.66)	0.73 (−1.80, 3.25)	0.51 (−1.52, 2.53)	0.35 (−2.10, 2.79)	0.13 (−2.46, 2.72)	a-tDCS-T3 + c-tDCS-Fp2	0.00 (−0.79, 0.79)	
*2.43 (0.61, 4.26)	1.12 (−0.24, 2.49)	1.14 (−0.17, 2.45)	1.12 (−0.61, 2.85)	0.84 (−0.46, 2.15)	0.72 (−0.04, 1.48)	0.73 (−1.05, 2.51)	0.51 (−0.43, 1.45)	0.35 (−1.32, 2.02)	0.13 (−1.74, 2.00)	−0.00 (−1.79, 1.79)	Sham	0.44 (−0.37, 1.26)
*2.88 (0.31, 5.45)	1.57 (−0.69, 3.83)	1.58 (−0.64, 3.81)	1.57 (−0.93, 4.07)	1.29 (−0.94, 3.51)	1.16 (−0.79, 3.12)	1.17 (−1.36, 3.71)	0.95 (−1.08, 2.98)	0.79 (−1.66, 3.25)	0.57 (−2.02, 3.17)	0.44 (−2.09, 2.98)	0.44 (−1.36, 2.25)	a-tDCS-F3 + c-tDCS-RtLb

Pairwise (upper-right portion) and network (lower-left portion) meta-analysis results are presented as estimate effect sizes for the outcome of changes of overall cognition function in patients with Alzheimer’s dementia. Interventions are reported in order of mean ranking of beneficial effect on overall cognition function, and outcomes are expressed as standardized mean difference (SMD) (95% confidence intervals). For the pairwise meta-analyses, SMD of more than 0 indicate that the treatment specified in the row got more beneficial effect than that specified in the column. For the network meta-analysis (NMA), SMD of more than 0 indicate that the treatment specified in the column got more beneficial effect than that specified in the row. Bold results marked with * indicate statistical significance

*Abbreviations*: c-tDCS-F3 + a-tDCS-Fp2, cathodal tDCS of the left DLPFC and anodal over the right supraorbital region; HF-rTMS-F3F4, high-frequency rTMS over bilateral DLPFC; a-tDCS-F3 + c-tDCS-Fp2, anodal tDCS of the left DLPFC and cathodal over the right supraorbital region; a-tDCS-T3P3/T4P4 + c-tDCS-LtLb, anodal tDCS 2mA alternatively over the bilateral temporo-parietal lobe (T3-P3 or T4-P4) and cathodal over left arm deltoid muscle; a-tDCS-F7 + c-tDCS-Fp2, anodal tDCS of the left frontotemporal lobe and cathodal over the right frontal lobe; HF-rTMS-Mx, high-frequency rTMS multifocal stimulation; HF-rTMS-F3T3, high-frequency rTMS over left DLPFC and left lateral temporal lobe; HF-rTMS-F3, high-frequency rTMS over left DLPFC; LF-rTMS-F3F4, low frequency rTMS over bilateral DLPFC; a-tDCS-F3 + c-tDCS-F4, anodal tDCS of the left DLPFC and cathodal over the right DLPFC; a-tDCS-T3 + c-tDCS-Fp2, anodal tDCS of the left lateral temporal lobe and cathodal over the right frontal lobe; a-tDCS-F3 + c-tDCS-RtLb, anodal tDCS of the left DLPFC and cathodal over the right deltoid muscle; DLPFC, dorsolateral prefrontal cortex; rTMS, repetitive transcranial magnetic stimulation; Sham, sham control; tDCS, transcranial direct current stimulation

Notably, the sham therapy effect did not significantly differ between the sham rTMS and tDCS therapies (*P* = .061). However, a significant placebo effect was observed in the sham rTMS group (SMD = 0.171 [95%CIs = 0.009–0.333], *P* = .038) but not in the sham tDCS group (SMD= −0.124 [95%CIs = −0.386 to 0.138], *P* = .355; eFigure 1).

### Subgroup of primary outcome: changes in cognitive function as measured by the MMSE

Only the HF-rTMS-F3F4 (MD = 6.11 [95%CIs = 2.38–9.83]), c-tDCS-F3 + a-tDCS-Fp2 (MD = 5.55 [95%CIs = 3.72–7.38]), a-tDCS-F3 + c-tDCS-Fp2 (MD = 4.61 [95%CIs = 2.75–6.47]), and anodal tDCS of the left frontotemporal lobe and cathodal tDCS over the right Fp2 region (a-tDCS-F7 + c-tDCS-Fp2; MD = 2.38 [95%CIs = 0.45–4.32]) were associated with significant beneficial effect on cognition (measured by MMSE) compared with sham controls (Table 2, Figs. 2B and 3B). The associations between an NIBS method and the changes of cognitive function (measured by MMSE) were ranked according to the SUCRA. In brief, HF-rTMS-F3F4 was associated with the greatest benefit, followed by c-tDCS-F3 + a-tDCS-Fp2 and a-tDCS-F3 + c-tDCS-Fp2 (eTable 6B).

**Table 2:** League table of the changes of overall cognition function: measured with MMSE

HF-rTMS-F3F4				*4.13 (0.29, 7.98)						*6.11 (2.62, 9.59)	
0.56 (−3.59, 4.71)	c-tDCS-F3 + a-tDCS-Fp2	0.94 (−0.31, 2.19)								*5.55 (4.28, 6.82)	
1.50 (−2.67, 5.66)	0.94 (−0.88, 2.76)	a-tDCS-F3 + c-tDCS-Fp2								*4.61 (3.30, 5.93)	
3.72 (−0.47, 7.92)	*3.17 (0.50, 5.83)	2.23 (−0.46, 4.91)	a-tDCS-F7 + c-tDCS-Fp2							2.47 (−0.15, 5.09)	
*4.13 (0.07, 8.20)	3.58 (−0.40, 7.56)	2.64 (−1.36, 6.63)	0.41 (−3.62, 4.44)	LF-rTMS-F3F4						1.97 (−1.31, 5.26)	
4.34 (−0.01, 8.68)	*3.78 (0.88, 6.67)	2.84 (−0.08, 5.76)	0.61 (−2.35, 3.57)	0.20 (−3.99, 4.39)	HF-rTMS-F3T3					1.77 (−0.05, 3.59)	
*5.40 (1.08, 9.72)	*4.85 (1.99, 7.70)	*3.91 (1.03, 6.78)	1.68 (−1.24, 4.60)	1.27 (−2.89, 5.43)	1.07 (−2.07, 4.20)	HF-rTMS-F3			1.64 (−3.62, 6.91)	0.55 (−1.60, 2.70)	
5.51 (−0.28, 11.29)	*4.95 (0.16, 9.74)	4.01 (−0.79, 8.81)	1.78 (−3.05, 6.62)	1.37 (−4.29, 7.04)	1.17 (−3.79, 6.14)	0.10 (−4.83, 5.04)	a-tDCS-F3 + c-tDCS-F4			0.60 (−3.63, 4.83)	
6.11 (−2.32, 14.54)	5.55 (−2.23, 13.33)	4.61 (−3.18, 12.40)	2.38 (−5.42, 10.19)	1.97 (−6.38, 10.32)	1.77 (−6.12, 9.66)	0.70 (−7.17, 8.58)	0.60 (−8.17, 9.37)	a-tDCS-T3 + c-tDCS-Fp2		0.00 (−7.45, 7.45)	
*6.12 (1.70, 10.55)	*5.57 (2.56, 8.57)	*4.63 (1.60, 7.65)	2.40 (−0.70, 5.50)	1.99 (−2.28, 6.26)	1.79 (−1.49, 5.06)	0.72 (−2.23, 3.67)	0.62 (−4.42, 5.65)	0.02 (−7.92, 7.95)	HF-rTMS-Mx	0.20 (−2.37, 2.77)	
*6.11 (2.38, 9.83)	*5.55 (3.72, 7.38)	*4.61 (2.75, 6.47)	*2.38 (0.45, 4.32)	1.97 (−1.56, 5.51)	1.77 (−0.47, 4.02)	0.70 (−1.48, 2.89)	0.60 (−3.83, 5.03)	−0.00 (−7.56, 7.56)	−0.02 (−2.40, 2.37)	Sham	1.40 (−1.03, 3.83)
*7.51 (2.87, 12.15)	*6.95 (3.63, 10.27)	*6.01 (2.67, 9.35)	*3.78 (0.41, 7.16)	3.37 (−1.12, 7.86)	3.17 (−0.39, 6.73)	2.10 (−1.42, 5.63)	2.00 (−3.22, 7.22)	1.40 (−6.65, 9.45)	1.38 (−2.27, 5.04)	1.40 (−1.37, 4.17)	a-tDCS-F3 + c-tDCS-RtLb

Pairwise (upper-right portion) and network (lower-left portion) meta-analysis results are presented as estimate effect sizes for the outcome of changes of cognition function in measurement of MMSE in patients with Alzheimer’s dementia. Interventions are reported in order of mean ranking of beneficial effect on cognition function in measurement of MMSE, and outcomes are expressed as mean difference (MD) (95% confidence intervals). For the pairwise meta-analyses, MD of more than 0 indicate that the treatment specified in the row got more beneficial effect than that specified in the column. For the network meta-analysis (NMA), MD of more than 0 indicate that the treatment specified in the column got more beneficial effect than that specified in the row. Bold results marked with * indicate statistical significance

*Abbreviations:* HF-rTMS-F3F4, high-frequency rTMS over bilateral DLPFC; c-tDCS-F3 + a-tDCS-Fp2, cathodal tDCS of the left DLPFC and anodal over the right supraorbital region; a-tDCS-F3 + c-tDCS-Fp2, anodal tDCS of the left DLPFC and cathodal over the right supraorbital region; a-tDCS-F7 + c-tDCS-Fp2, anodal tDCS of the left frontotemporal lobe and cathodal over the right frontal lobe; LF-rTMS-F3F4, low frequency rTMS over bilateral DLPFC; HF-rTMS-F3T3, high-frequency rTMS over left DLPFC and left lateral temporal lobe; HF-rTMS-F3, high-frequency rTMS over left DLPFC; a-tDCS-F3 + c-tDCS-F4, anodal tDCS of the left DLPFC and cathodal over the right DLPFC; a-tDCS-T3 + c-tDCS-Fp2, anodal tDCS of the left lateral temporal lobe and cathodal over the right frontal lobe; HF-rTMS-Mx, high-frequency rTMS multifocal stimulation; a-tDCS-F3 + c-tDCS-RtLb, anodal tDCS of the left DLPFC and cathodal over the right deltoid muscle; MMSE: mini-mental state examination; DLPFC, dorsolateral prefrontal cortex; rTMS, repetitive transcranial magnetic stimulation; Sham, sham control; tDCS, transcranial direct current stimulation

### Subgroup of primary outcome: changes in cognitive function as measured by the ADAS-Cog

The NMA revealed that only the high-frequency rTMS over the left DLPFC (HF-rTMS-F3; MD = −3.40 [95%CIs = −6.57 to −0.23]) and high-frequency rTMS multifocal stimulation (HF-rTMS-Mx; MD = −3.06 [95%CIs = −4.55 to −1.57]) were associated with significant beneficial effect on cognition (measured by ADAS-Cog) compared with sham controls (Table 3, Figs. 2C and 3C). The associations between NIBS interventions and benefit of cognitive decline (measured by ADAS-Cog) were ranked according to the SUCRA. In brief, HF-rTMS-F3F4 was associated with the greatest benefit (MD = −4.00 [95%CIs = −8.73 to 0.73]) compared with sham controls, followed by HF-rTMS-F3 and HF-rTMS-Mx (eTable 6C).

**Table 3:** League table of the changes of overall cognition function: measured with ADAS-Cog

HF-rTMS-F3F4				−4.00 (−8.28, 0.28)	
−0.60 (−6.30, 5.09)	HF-rTMS-F3	0.10 (−7.21, 7.40)		*−3.49 (−6.33, −0.65)	
−0.94 (−5.90, 4.02)	−0.34 (−3.74, 3.06)	HF-rTMS-Mx		*−3.03 (−4.43, −1.63)	
−2.02 (−7.39, 3.35)	−1.42 (−5.49, 2.65)	−1.08 (−4.03, 1.87)	HF-rTMS-F3T3	*−1.98 (−3.54, −0.42)	
−4.00 (−8.73, 0.73)	*−3.40 (−6.57, −0.23)	*−3.06 (−4.55, −1.57)	−1.98 (−4.53, 0.57)	Sham	−1.10 (−4.04, 1.84)
−5.10 (−11.02, 0.82)	−4.50 (−9.27, 0.27)	*−4.16 (−8.02, −0.30)	−3.08 (−7.46, 1.30)	−1.10 (−4.66, 2.46)	a-tDCS-F3 + c-tDCS-Fp2

Pairwise (upper-right portion) and network (lower-left portion) meta-analysis results are presented as estimate effect sizes for the outcome of changes of cognition function in measurement of ADAS-Cog in patients with Alzheimer’s dementia. Interventions are reported in order of mean ranking of beneficial effect on cognition function in measurement of ADAS-Cog, and outcomes are expressed as mean difference (MD) (95% confidence intervals). For the pairwise meta-analyses, MD of less than 0 indicate that the treatment specified in the row got more beneficial effect than that specified in the column. For the network meta-analysis (NMA), MD of less than 0 indicate that the treatment specified in the column got more beneficial effect than that specified in the row. Bold results marked with * indicate statistical significance

*Abbreviations: ADAS-Cog*, Alzheimer’s disease assessment scale-cognitive subscale; *HF-rTMS-F3F4*, high-frequency rTMS over bilateral DLPFC; *HF-rTMS-F3*, high-frequency rTMS over left DLPFC; *HF-rTMS-Mx*, high-frequency rTMS multifocal stimulation; *HF-rTMS-F3T3*, high-frequency rTMS over left DLPFC and left lateral temporal lobe; *a-tDCS-F3 + c-tDCS-Fp2*, anodal tDCS of the left DLPFC and cathodal over the right supraorbital region; *DLPFC*, dorsolateral prefrontal cortex; *rTMS*, repetitive transcranial magnetic stimulation; *Sham*, sham control; *tDCS*, transcranial direct current stimulation

### Secondary outcome: changes in quality of life

None of the NIBS methods were associated with differences in changes of quality of life compared with sham controls (eTable 5A, eTable 6D, eFigure 2A, and eFigure 3A).

### Safety profile: any adverse event rate

The NMA revealed that none of the NIBS methods were associated with different rates of any adverse events compared with sham controls (eTable 5B, eTable 6E, eFigure 2B, and eFigure 3B).

### Safety profile: any local discomfort rate

No differences between the NIBS interventions and sham controls with regard to local discomfort rate were observed (eTable 5C, eTable 6F, eFigure 2C, and eFigure 3C).

### Intervention acceptability: dropout rate

No differences in dropout rate between the NIBS interventions and sham controls were noted (eTable 5D, eTable 6G, eFigure 2D, and eFigure 3D).

### Risk of bias, publication bias, inconsistency, and GRADE ratings

Overall, 74.4% (99/133 items), 24.1% (32/133 items), and 1.5% (2/133 items) of the included studies had a low, unclear, and high risk of bias, respectively, to which unclear reporting of the allocation procedure was the major contributor of unclear risk of bias (eFigure 4A-B). Funnel plots of publication bias revealed general symmetry and Egger’s test results indicated no significant publication bias among the included articles (eFigure 5A-N). Overall, no inconsistencies in the NMA were demonstrated in either local inconsistencies, as assessed using the loop-specific approach and node-splitting method, or global inconsistencies, as determined using the design-by-treatment method, with the exception of significant inconsistency in the primary outcome (design-by-treatment method: *P* = .0125) (eTable 7-8). However, this inconsistency disappeared in the subgroup of the primary outcome: changes of cognitive function measured by MMSE or ADAS-Cog (design-by-treatment method: *P* = .7001 and .9044, respectively). The results of GRADE evaluation are listed in eTable 9. In brief, the overall quality of evidence of the overall NMA, direct evidence, and indirect evidence were low to medium.

## Discussion

To the best of our knowledge, this is the first comprehensive NMA investigating the efficacy and safety of NIBS interventions in patients with AD. As mentioned, c-tDCS-F3 + a-tDCS-Fp2 was associated with significant beneficial effect on cognition compared with sham controls. When focusing on specific cognition-rating scales, HF-rTMS-F3F4, c-tDCS-F3 + a-tDCS-Fp2, and a-tDCS-F3 + c-tDCS-Fp2 were determined to be associated with significant beneficial effect on cognition (measured by MMSE) compared with sham controls. Only HF-rTMS-F3 and HF-rTMS-Mx were associated with significant beneficial effect on cognition (measured by ADAS-Cog) compared with sham controls. Notably, a study reported significant placebo effects in sham rTMS therapy [74]. In addition, all the NIBS methods were well tolerated with regard to safety profile, as reflected in the rates of adverse events or local discomfort, as well as acceptability, as indicated by dropout rate.

The first main finding of this study was that the c-tDCS-F3 + a-tDCS-Fp2 was associated with a significant beneficial effect on cognition compared with sham controls and was also associated with the greatest beneficial effect on cognition among all the NIBS methods. The role of tDCS in changes of cognitive function in patients with AD is believed to be associated with the cognitive-enhancing effect of tDCS targeting the DLPFC, an area widely connected to cortical/subcortical regions and associated with executive control and memory [75]. However, the effect of the direction of tDCS currents on the specific cortical region is under debate. Although cathodal tDCS is generally believed to be associated with inhibitory effects on the targeted cortex, tDCS administered at higher intensity (i.e., 2mA) appears to result in reversal to excitatory effects [15]. Studies have indicated that these cortical effects outlast the original stimulation due to synaptic long-term potentiation [10, 76]. Another hypothetical mechanism regarding the cognitive benefits of tDCS involves the concerns about the activation of the cognitive reserve pool through acetylcholine and dopamine modulation [17, 18]. In addition, the beneficial effect of tDCS on cognitive function might be derived not only from cortical targeting but also from the effect of currents spreading to nearby cortical regions [77]. Taken together, the potential beneficial effect of c-tDCS-F3 + a-tDCS-Fp2 on the beneficial effect on cognition, if any, may involve different effects on multiple cortical regions. Notably, the finding of a beneficial cognitive effect of c-tDCS-F3 + a-tDCS-Fp2 was mainly drawn from a single study using small sample sizes (*n* = 12 and 11) [10]. In addition, its confidence interval was relatively wide (i.e., 95%CIs = 0.61–4.26). Therefore, it should be interpreted with caution, and future large-scale RCTs are warranted to provide more evidence.

Significant inconsistency in the primary outcome was noted overall (design-by-treatment method: *P* = .0125). However, this inconsistency disappeared in the subgroup of the primary outcome: changes of cognitive function measured by MMSE or ADAS-Cog (design-by-treatment method: *P* = .7001 and .9044, respectively). When we reexamined the evidence of the individual treatment arm, we found that the potential beneficial effect of a-tDCS-F3 + c-tDCS-Fp2 was also demonstrated in the subgroup analysis of changes of cognitive decline as measured by MMSE. In the overall NMA results, a-tDCS-F3 + c-tDCS-Fp2 did not achieve significant outcomes, in contrast to the fact that anodal tDCS targeted to the left DLPFC and left temporal cortex, among other cortices, has been reported to enhance executive function and the memory process [12, 13]. The nonsignificant result may be ascribable to the relatively shorter follow-up duration in one RCT, in which the a-tDCS-F3 + c-tDCS-Fp2 and sham control groups did not differ significantly in cognitive changes at a 3-week follow-up [72]. RCTs have shown that patients with AD receiving sham tDCS exhibited improvement in cognitive function but returned to baseline in a longer follow-up (i.e., 2-6 mo) [10, 43]. In the present study, we tried to reduce the potential impacts of time by extracting data at the final follow-up. Nevertheless, the relatively short follow-up duration (mean follow-up duration was 10.5 wks) may have confounded with time. Therefore, future RCTs are encouraged to use longer follow-up duration (i.e., at least 2–6 mo).

In the present NMA, the tDCS studies had relatively consistent findings. By contrast, those of the rTMS studies were inconsistent. This may be partially attributed to bias in the rTMS studies, such as the significant placebo effect observed in sham rTMS (SMD = 0.171, 95%CIs = 0.009-0.333, *P* = .038, eFigure 1). The placebo effect of NIBS interventions on cognitive outcomes may be associated with several mechanisms. First, because mimicking active rTMS is challenging, several modifications have been developed for rTMS, such as the application of peripheral auditory clicking sounds for intersensory facilitation [78]. The potential placebo effect may also result from the introduction of the intervention itself, the expectation of outcomes, optimism, and emotional goal-seeking [79]. Therefore, as suggested in one study, the alleviation of negative emotion (i.e., depressive mood) in older adults and the presence or company of study staff members may contribute to cognitive improvement [70]. To reduce the potential bias from the placebo effect, the development of adequate rTMS sham control interventions should be urgent. Finally, the electromagnetic field of standard coils decays rapidly, and they can only penetrate to a depth of 2–3 cm [80], which may be insufficient to reach the outer brain layers of older adults and patients with brain atrophy, both characteristics of patients with AD. Therefore, the potential of alternative rTMS techniques that can reach deeper structures (e.g., deep TMS) should be explored.

### Limitations

This study has several limitations. First, this NMA may have been underpowered because of the heterogeneity of the participants (e.g., with regard to comorbidities, effects of concomitant medications on cognition, baseline AD severity, differences in AD onset age, different methods to define the stimulation target, different NIBS stimulation protocols, and follow-up duration). Second, although all the RCTs included a sham control in their study design, they may not have been adequately blinded because of differences in the interventions used. The placebo effect by the sham control, especially that of sham rTMS (SMD = 0.171, 95%CIs = 0.009–0.333, *P* = .038, eFigure 1), may also have imposed bias with regard to patients with AD [70]. Third, given the relatively small number of RCTs (and by extension patients) included, the main present findings should perhaps be conservatively applied in clinical practice. Specifically, the evidence of potentially beneficial effect of c-tDCS-F3 + a-tDCS-Fp2 on cognition in patients with AD was mainly derived from one RCT [10], which evaluated patients with mild-to-moderate AD (MMSE score between 11 and 23) with a 10-week follow-up. Therefore, the corresponding finding in the present study should be limited to patients with the same severity of AD that were followed up for at least 10 weeks. Fourth, whether tDCS can target specific cortices remains under debate [77]. Therefore, whether the potential beneficial effect of tDCS resulted from enhancement on the specific cortex remains unclear. Fifth, although most of the included RCTs provided comparisons between NIBS and sham control interventions, few provided comparisons between different NIBS interventions [2, 10, 66]. Therefore, the geometric structure of the current NMA was relatively weak (Fig. 2A–C). Sixth, although we intended to also include RCTs of deep TMS and TBS by adding these modalities in our search keywords, there was a lack of such RCTs available at present time. Finally, significant inconsistency was detected in the primary outcome overall. Although this inconsistency disappeared after subgroup analysis, clinicians should remain cautious in practical applications of those findings.

## Conclusion

In summary, c-tDCS-F3 + a-tDCS-Fp2 was associated with significant beneficial effect on cognition compared with sham control. Moreover, it was associated with the greatest benefit of cognitive decline among all the NIBS interventions. In addition, all of the methods were suggested to be well tolerated with regard to safety profile, as reflected in the rates of adverse events or local discomfort, as well as acceptability, as indicated by dropout rate. The significant placebo effect in sham rTMS indicates that adequate sham control interventions in rTMS therapy should be urgently developed. However, because the treatment duration of the included studies was relatively short (mean=5.7 weeks, IQR = 2–6 weeks), future RCTs of tDCS are encouraged to use longer treatment duration to obtain more evidence of the beneficial effect by long-term NIBS interventions.

## Supplementary Information

**Additional file 1: eTable 1.** PRISMA 2020 checklist of the current network meta-analysis. **eTable 2.** Keyword used in each database and search results. **eTable 3.** Excluded studies and reason. **eTable 4.** Characteristics of the included studies. **eTable 5.** A League table of the changes of quality of life. B: League table of the rate of any adverse event. C: League table of the rate of local discomfort. D:League table of the drop-out rate. **eTable 6.** A SUCRA of the changes of cognition function-overall. B: SUCRA of the changes of cognition function: measured with MMSE. C: SUCRA of the changes of cognition function: measured with ADAS-Cog. D: SUCRA of the changes of quality of life. E: SUCRA of the rate of any adverse event. F: SUCRA of the rate of local discomfort. G: SUCRA of the drop-out rate. **eTable 7.** Inconsistency of different intervention. **eTable 8.** Estimated between-studies standard deviation of different outcome. **eTable 9.** GRADE evaluation quality of evidence for primary outcome. **eFigure1.** Test for transitivity assumption of primary outcome: changes of cognition function-overall. **eFigure2.** A network structure of NMA of changes of quality of life. B network structure of NMA of safety profile in aspect of rate of any adverse event. C network structure of NMA of safety profile in aspect of rate of any local discomfort. D network structure of NMA of acceptability in aspect of drop-out rate. **eFigure3.** A forest plot of NMA of change of quality of life. B forest plot of NMA of safety profile in aspect of rate of any adverse event. C forest plot of NMA of safety profile in aspect of rate of any local discomfort. D forest plot of NMA of acceptability in aspect of drop-out rate. **eFigure4.** A overview of risk of bias. B detailed risk of bias in each study. **eFigure5.** A Funnel plot of changes of cognition function: overall. B Egger’s regression of changes of cognition function: overall. C Funnel plot of changes of cognition function: MMSE measurement. D Egger’s regression of changes of cognition function: MMSE measurement. E Funnel plot of changes of cognition function: ADAS-Cog measurement. F Egger’s regression of changes of cognition function: ADAS-Cog measurement. G Funnel plot of changes of quality of life. H Egger’s regression of changes of quality of life. I Funnel plot of safety profile in aspect of rate of any adverse event. J Egger’s regression of safety profile in aspect of rate of any adverse event. K Funnel plot of safety profile in aspect of rate of any local discomfort. L Egger’s regression of safety profile in aspect of rate of any local discomfort. M Funnel plot of acceptability in aspect of drop-out rate. N Egger’s regression of acceptability in aspect of drop-out rate.

## Data Availability

All the data and materials of the current study were available upon reasonable request.

## Abbreviations

AD: Alzheimer’s disease
ADAS-Cog: Alzheimer’s disease assessment scale-cognitive subscale
a-tDCS-F3 + c-tDCS-F4: Anodal tDCS of the left DLPFC and cathodal over the right DLPFC
a-tDCS-F3 + c-tDCS-Fp2: Anodal tDCS of the left DLPFC and cathodal over the right supraorbital region
a-tDCS-F3 + c-tDCS-RtLb: Anodal tDCS of the left DLPFC and cathodal over the right deltoid muscle
a-tDCS-F7 + c-tDCS-Fp2: Anodal tDCS of the left frontotemporal lobe and cathodal over the right frontal lobe
a-tDCS-T3 + c-tDCS-Fp2: Anodal tDCS of the left lateral temporal lobe and cathodal over the right frontal lobe
a-tDCS-T3 + c-tDCS-RtLb: Anodal tDCS of the left lateral temporal lobe and cathodal over the right upper limb
a-tDCS-T3P3/T4P4 + c-tDCS-LtLb: Anodal tDCS 2mA alternatively over the bilateral temporo-parietal lobe (T3-P3 or T4-P4) and cathodal over left arm deltoid muscle
CDR: Clinical Dementia Rating
CI: Confidence interval
c-tDCS-F3 + a-tDCS-Fp2: Cathodal tDCS of the left DLPFC and anodal over the right supraorbital region
DLPFC: Dorsolateral prefrontal cortex
dTMS: Deep TMS
HF-rTMS: High-frequency rTMS
HF-rTMS-F3: High-frequency rTMS over left DLPFC
HF-rTMS-F3F4: High-frequency rTMS over bilateral DLPFC
HF-rTMS-F3T3: High-frequency rTMS over left DLPFC and left lateral temporal lobe
HF-rTMS-F4: High-frequency rTMS over right DLPFC
HF-rTMS-Mx: High-frequency rTMS multifocal stimulation
IQR: Interquartile range
LF-rTMS: Low-frequency rTMS
LF-rTMS-F3F4: Low-frequency rTMS over bilateral DLPFC
MD: Mean difference
MMSE: Mini-Mental State Examination
NIBS: Noninvasive brain stimulation
NMA: Network meta-analysis
OR: Odds ratio
PRISMA: Preferred Reporting Items for Systematic Reviews and Meta-Analyses
RCT: Randomized controlled trial
rTMS: Repetitive transcranial magnetic stimulation
Sham: Sham control
SMD: Standardized mean difference
SUCRA: Surface under the cumulative ranking curve
TBS: Theta-burst stimulation
tDCS: Transcranial direct current stimulation
