# Supplementary material for: The beneficial effect on cognition of noninvasive brain stimulation intervention in patients with dementia: a network meta-analysis of randomized controlled trials
Source: Alzheimers Res Ther. 2023 Jan 25;15:20. doi: 10.1186/s13195-023-01164-2 (PMC9875424; doi:10.1186/s13195-023-01164-2)
Supplement: Supplementary file 1 — Additional file 1: eTable 1. PRISMA 2020 checklist of the current network meta-analysis. eTable 2. Keyword used in each database and search results. eTable 3. Excluded studies and reason. eTable 4. Characteristics of the included studies. eTable 5. A League table of the changes of quality of life. B: League table of the rate of any adverse event. C: League table of the rate of local discomfort. D:League table of the drop-out rate. eTable 6. A SUCRA of the changes of cognition function-overall. B: SUCRA of the changes of cognition function: measured with MMSE. C: SUCRA of the changes of cognition function: measured with ADAS-Cog. D: SUCRA of the changes of quality of life. E: SUCRA of the rate of any adverse event. F: SUCRA of the rate of local discomfort. G: SUCRA of the drop-out rate. eTable 7. Inconsistency of different intervention. eTable 8. Estimated between-studies standard deviation of different outcome. eTable 9. GRADE evaluation quality of evidence for primary outcome. eFigure1. Test for transitivity assumption of primary outcome: changes of cognition function-overall. eFigure2. A network structure of NMA of changes of quality of life. B network structure of NMA of safety profile in aspect of rate of any adverse event. C network structure of NMA of safety profile in aspect of rate of any local discomfort. D network structure of NMA of acceptability in aspect of drop-out rate. eFigure3. A forest plot of NMA of change of quality of life. B forest plot of NMA of safety profile in aspect of rate of any adverse event. C forest plot of NMA of safety profile in aspect of rate of any local discomfort. D forest plot of NMA of acceptability in aspect of drop-out rate. eFigure4. A overview of risk of bias. B detailed risk of bias in each study. eFigure5. A Funnel plot of changes of cognition function: overall. B Egger’s regression of changes of cognition function: overall. C Funnel plot of changes of cognition function: MMSE measurement. D Egger’s regression of change [file 13195_2023_1164_MOESM1_ESM.pdf]

eTable 1: PRISMA 2020 checklist of the current network meta-analysis

Section and Topic	Item #	Checklist item	Page where item is reported
**TITLE**			
Title	1	Identify the report as a systematic review.	1
**ABSTRACT**			
Abstract	2	See the PRISMA 2020 for Abstracts checklist.	5
**INTRODUCTION**			
Rationale	3	Describe the rationale for the review in the context of existing knowledge.	7-8
Objectives	4	Provide an explicit statement of the objective(s) or question(s) the review addresses.	7-8
**METHODS**			
Eligibility criteria	5	Specify the inclusion and exclusion criteria for the review and how studies were grouped for the syntheses.	9-10
Information sources	6	Specify all databases, registers, websites, organisations, reference lists and other sources searched or consulted to identify studies. Specify the date when each source was last searched or consulted.	9-10
Search strategy	7	Present the full search strategies for all databases, registers and websites, including any filters and limits used.	9-10
Selection process	8	Specify the methods used to decide whether a study met the inclusion criteria of the review, including how many reviewers screened each record and each report retrieved, whether they worked independently, and if applicable, details of automation tools used in the process.	9-10
Data collection process	9	Specify the methods used to collect data from reports, including how many reviewers collected data from each report, whether they worked independently, any processes for obtaining or confirming data from study investigators, and if applicable, details of automation tools used in the process.	9-10
Data items	10a	List and define all outcomes for which data were sought. Specify whether all results that were compatible with each outcome domain in each study were sought (e.g. for all measures, time points, analyses), and if not, the methods used to decide which results to collect.	10-11
	10b	List and define all other variables for which data were sought (e.g. participant and intervention characteristics, funding sources). Describe any assumptions made about any missing or unclear information.	10-11
Study risk of bias assessment	11	Specify the methods used to assess risk of bias in the included studies, including details of the tool(s) used, how many reviewers assessed each study and whether they worked independently, and if applicable, details of automation tools used in the process.	10-11
Effect measures	12	Specify for each outcome the effect measure(s) (e.g. risk ratio, mean difference) used in the synthesis or presentation of results.	10-11
Synthesis methods	13a	Describe the processes used to decide which studies were eligible for each synthesis (e.g. tabulating the study intervention characteristics and comparing against the planned groups for each synthesis (item #5)).	11-12
	13b	Describe any methods required to prepare the data for presentation or synthesis, such as handling of missing summary statistics, or data conversions.	11-12
	13c	Describe any methods used to tabulate or visually display results of individual studies and syntheses.	11-12
	13d	Describe any methods used to synthesize results and provide a rationale for the choice(s). If meta-analysis was performed, describe the model(s), method(s) to identify the presence and extent of statistical heterogeneity, and software package(s) used.	11-12
	13e	Describe any methods used to explore possible causes of heterogeneity among study results (e.g. subgroup analysis, meta-regression).	12-13
	13f	Describe any sensitivity analyses conducted to assess robustness of the synthesized results.	12-13
Reporting bias assessment	14	Describe any methods used to assess risk of bias due to missing results in a synthesis (arising from reporting biases).	12-13
Certainty assessment	15	Describe any methods used to assess certainty (or confidence) in the body of evidence for an outcome.	12-13
**RESULTS**			

Section and Topic	Item #	Checklist item	Page where item is reported
Study selection	16a	Describe the results of the search and selection process, from the number of records identified in the search to the number of studies included in the review, ideally using a flow diagram.	14-15, Fig 1, eTab 2
	16b	Cite studies that might appear to meet the inclusion criteria, but which were excluded, and explain why they were excluded.	14-15, eTab 3
Study characteristics	17	Cite each included study and present its characteristics.	14-15, eTab 4
Risk of bias in studies	18	Present assessments of risk of bias for each included study.	14-15, eFig 4
Results of individual studies	19	For all outcomes, present, for each study: (a) summary statistics for each group (where appropriate) and (b) an effect estimate and its precision (e.g. confidence/credible interval), ideally using structured tables or plots.	14-15, eTab 4
Results of syntheses	20a	For each synthesis, briefly summarise the characteristics and risk of bias among contributing studies.	15-16, Fig 2
	20b	Present results of all statistical syntheses conducted. If meta-analysis was done, present for each the summary estimate and its precision (e.g. confidence/credible interval) and measures of statistical heterogeneity. If comparing groups, describe the direction of the effect.	15-16, Fig 3
	20c	Present results of all investigations of possible causes of heterogeneity among study results.	15-16, eTab 7-8
	20d	Present results of all sensitivity analyses conducted to assess the robustness of the synthesized results.	15-16
Reporting biases	21	Present assessments of risk of bias due to missing results (arising from reporting biases) for each synthesis assessed.	16-17, eFig 4
Certainty of evidence	22	Present assessments of certainty (or confidence) in the body of evidence for each outcome assessed.	16-17
**DISCUSSION**			
Discussion	23a	Provide a general interpretation of the results in the context of other evidence.	18-20
	23b	Discuss any limitations of the evidence included in the review.	20-21
	23c	Discuss any limitations of the review processes used.	20-21
	23d	Discuss implications of the results for practice, policy, and future research.	21
**OTHER INFORMATION**			
Registration and protocol	24a	Provide registration information for the review, including register name and registration number, or state that the review was not registered.	5
	24b	Indicate where the review protocol can be accessed, or state that a protocol was not prepared.	5
	24c	Describe and explain any amendments to information provided at registration or in the protocol.	5
Support	25	Describe sources of financial or non-financial support for the review, and the role of the funders or sponsors in the review.	22
Competing interests	26	Declare any competing interests of review authors.	22
Availability of data, code and other materials	27	Report which of the following are publicly available and where they can be found: template data collection forms; data extracted from included studies; data used for all analyses; analytic code; any other materials used in the review.	22

The current checklist followed the latest PRISMA 2020 guideline [1].

eTable 2: Keyword used in each database and search results

Database	Keyword	Filter	Date	Result
PubMed	(deep transcranial magnetic stimulation OR dTMS OR repetitive transcranial magnetic stimulation OR rTMS OR TMS OR non-invasive brain stimulation OR theta burst stimulation OR transcranial direct current stimulation OR TBS OR tDCS OR vagus nerve stimulation OR vagal nerve stimulation OR tVNS OR nVNS OR VNS OR static magnetic field stimulation OR SMS OR tSMS OR colon electric stimulation) AND (Alzheimer disease OR Alzheimer's disease OR Alzheimer dementia OR Alzheimer’s dementia OR dementia OR senile dementia) AND (random OR randomized OR randomised)	NA	2020/11/26	147
Embase	(deep transcranial magnetic stimulation OR dTMS OR repetitive transcranial magnetic stimulation OR rTMS OR TMS OR non-invasive brain stimulation OR theta burst stimulation OR transcranial direct current stimulation OR TBS OR tDCS OR vagus nerve stimulation OR vagal nerve stimulation OR tVNS OR nVNS OR VNS OR static magnetic field stimulation OR SMS OR tSMS OR colon electric stimulation) AND (Alzheimer OR Alzheimer disease OR Alzheimer dementia) AND (random OR randomized OR randomised)	NA	2020/11/26	210
ClinicalKey	(non-invasive brain stimulation) AND (Alzheimer OR Alzheimer disease OR Alzheimer dementia)	NA	2020/11/26	728
Cochrane CENTRAL	(deep transcranial magnetic stimulation OR dTMS OR repetitive transcranial magnetic stimulation OR rTMS OR TMS OR non-invasive brain stimulation OR theta	NA	2020/11/26	240

	burst stimulation OR transcranial direct current stimulation OR TBS OR tDCS OR vagus nerve stimulation OR vagal nerve stimulation OR tVNS OR nVNS OR VNS OR static magnetic field stimulation OR SMS OR tSMS OR colon electric stimulation) AND (Alzheimer disease OR Alzheimer's disease OR Alzheimer dementia OR Alzheimer’s dementia OR dementia OR senile dementia) AND (random OR randomized OR randomised)			
ProQuest	(non-invasive brain stimulation) AND (Alzheimer OR Alzheimer disease OR Alzheimer dementia)	NA	2020/11/26	4798
ScienceDirect	(non-invasive brain stimulation) AND (Alzheimer OR Alzheimer disease OR Alzheimer dementia)	research article	2020/11/26	2438
Web of Science	(deep transcranial magnetic stimulation OR dTMS OR repetitive transcranial magnetic stimulation OR rTMS OR TMS OR non-invasive brain stimulation OR theta burst stimulation OR transcranial direct current stimulation OR TBS OR tDCS OR vagus nerve stimulation OR vagal nerve stimulation OR tVNS OR nVNS OR VNS OR static magnetic field stimulation OR SMS OR tSMS OR colon electric stimulation) AND (Alzheimer disease OR Alzheimer's disease OR Alzheimer dementia OR Alzheimer’s dementia OR dementia OR senile dementia) AND (random OR randomized OR randomised)	NA	2020/11/26	111
ClinicalTrials.gov	(non-invasive brain stimulation) AND (Alzheimer OR Alzheimer disease OR Alzheimer dementia)	NA	2020/11/26	8

Abbreviation: NA: not applied

eTable 3: Excluded studies and reason

Reason	Numbers	References
Diagnostic evaluation but not intervention evaluation	1	[2]
Investigate brain plasticity and working memory but not overall cognitive function	1	[3]
Meta-analysis	11	[4-14]
Mild cognition decline but not diagnosis of Alzheimer disease	3	[15-17]
Network meta-analysis	1	[18]
Not all the included patients had diagnosis of Alzheimer disease	22	[19-40]
Not associated with non-invasive brain stimulation intervention	1	[41]
Not randomized controlled trial	5	[42-46]
Only one-two sessions of stimulation but not a course of sessions stimulation	7	[47-53]
Protocol but not report of study result	13	[54-66]
Review article	4	[67-70]

eTable 4: Characteristics of the included studies

Study	Baseline AD severity	Comparison	Subjects	mean age	female (%)	baseline MMSE	Tx duration (weeks)	Follow-up (weeks)#	Country
Brem, A.K. (2020)[71]	mild to moderate, MMSE 24-18	real cognitive training combined with real rTMS 10 Hz multifocal stimulation (R/L DLPFC, R/L IPL, L STG, L IFG) (real/real)	16	69.3±6.8	75.0	21.2±2.7	6	12	Multiple countries
		real cognitive training and sham rTMS (real/sham)	10	69.1±5.2	50.0	22.0±1.8			
Gangemi, A. (2020) study 1[72]	mild to moderate, MMSE 20-14	anodal tDCS 2mA of the left frontotemporal lobe and cathodal over right frontal lobe	13	67.5±2.8	NA	14.9±1.8	2	2	Italy
		Sham control	13	69.0±3.1		15.3±1.8			
Gangemi, A. (2020) study 2[72]	mild to moderate, MMSE 20-14	anodal tDCS 2mA of the left frontotemporal lobe and cathodal over right frontal lobe	9	68.5±2.8	NA	15.8±1.8	32	32	Italy
		Sham control	9	68.7±3.1		15.9±1.6			
Padala, P.R. (2020)[73]	mild to moderate, MMSE >= 18	10Hz rTMS 3000 pulses over left DLPFC	9	74.3±5.7	11.1	22.9±3.4	4	12	USA
		Sham control	11	79.6±7.7	9.1	21.4±3.3			
Sabbagh, M. (2020)[74]	mild to moderate, MMSE 26-18	rTMS 10 Hz 1300 pulses multifocal stimulation (Broca's area; Wernicke's area; left/right DLPFC; left/right inferior parietal lobule)	79	76.9	48.1	21.7	6	12	Multiple countries
		Sham control	50	76.7	42.0	21.3			
Im, J.J. (2019)[75]	mild to moderate	anodal tDCS 2mA of the left DLPFC and cathodal over the right DLPFC	11	71.9±9.2	90.9	20.1±3.8	24	24	South Korea
		Sham control	7	74.9±5.0	71.4	22.1±4.6			

Khedr, E.M. (2019)[76]	mild to moderate	anodal tDCS 2mA alternatively over the bilateral temporo-parietal lobe and cathodal over left arm deltoid muscle<br>Sham control	23<br>21	64.2±3.6<br>65.2±4.5	43.5<br>38.1	45.5±10.0<br>48.8±12.7<br>(Modified MMSE)	2	2	Egypt
Zhang, F. (2019)[77]	mild to moderate	10Hz rTMS 1000 pulses over left DLPFC and left lateral temporal lobe<br>Sham control	15<br>13	69.0±8.2<br>68.5±7.9	80.0<br>76.9	20.5±4.2<br>19.8±5.1	4	8	China
Alcala-Lozano, R. (2018)[78]	mild to moderate, MMSE >= 15	5Hz rTMS 1500 pulses over left DLPFC<br>5 Hz rTMS 1500 pulses multifocal stimulation (Broca's area; Wernicke's area; left/right DLPFC; left/right parietal somatosensory association cortex)	10<br>9	73.3±6.0<br>71.0±4.3	60.0<br>55.6	NA	3	7	USA
Zhao, J. (2017)[79]	mild to moderate, MMSE 26-18	rTMS 20 Hz multifocal stimulation (bilateral parietal and bilateral posterior temporal)<br>Sham control	17<br>13	69.3±5.8<br>71.4±5.2	58.8<br>54.8	22.2±2.8<br>22.8±2.3	6	12	China
Bystad, M. (2016)[80]	mild to moderate, MMSE >= 18	anodal tDCS 2mA of the left lateral temporal lobe and cathodal over right frontal lobe<br>Sham control	12<br>13	70.0±8.0<br>75.0±8.7	41.7<br>46.2	20.0±2.8<br>21.2±3.9	2	2	Norway
Lee, J. (2016)[81]	mild to moderate, MMSE 26-18	rTMS 10 Hz 1200 pulses multifocal stimulation (Broca's area; Wernicke's area; left/right DLPFC; left/right parietal somatosensory association cortex)<br>Sham control	18<br>8	72.1±7.6<br>70.3±4.8	55.6<br>62.5	22.4±2.9<br>22.8±2.5	6	12	South Korea
Rutherford, G. (2015) Stage 1[82]	not mentioned	20Hz rTMS 2000 pulses over bilateral DLPFC<br>Sham control	6<br>4	NA	60	NA	4	4	Canada
Wu, Y. (2015)[83]	mild, moderate, and severe, MMSE < 24	20Hz rTMS 1200 pulses over left DLPFC<br>Sham control	26<br>26	71.4±4.9<br>71.9±4.8	61.5<br>57.7	15.3±3.1<br>15.2±3.1	4	4	China

Cotelli, M. (2014)[84]	mild to moderate	anodal tDCS 2mA of the left DLPFC and cathodal over the right deltoid muscle plus individualized computerized memory training	12	76.6±4.6	83.3	20.1±2.4	2	26	Italy
		Sham control plus individualized computerized memory training	12	74.7±6.1	75.0	20.8±2.1			
Khedr, E.M. (2014)[85]	mild to moderate, MMSE 23-11	anodal tDCS 2mA of the left DLPFC and cathodal over right supraorbital region	11	68.5±7.2	45.5	18.4±3.9	2	10	Egypt
		cathodal tDCS 2mA of the left DLPFC and anodal over right supraorbital region	12	70.7±5.4	33.3	18.8±2.9			
		Sham control	11	67.3±5.9	54.5	16.9±2.9			
Suemoto, C.K. (2014)[86]	mild to moderate, MMSE 20-10	anodal tDCS 2mA of the left DLPFC and cathodal over right supraorbital region	20	79.4±7.1	75.0	15.0±3.1	2	3	Brazil
		Sham control	20	81.6±8.0	65.0	15.4±2.6			
Rabey, J.M. (2013)[87]	mild to moderate, MMSE 24-18	rTMS 10 Hz 1300 pulses multifocal stimulation (Broca's area; Wernicke's area; left/right DLPFC; left/right parietal somatosensory association cortex)	7	72.6±8.9	28.6	22.0±1.6	18	18	Israel
		Sham control	8	75.4±9.1	37.6	22.0±1.4			
Ahmed, M.A. (2012)[88]	mild, moderate, and severe, MMSE 21 - 6	20Hz rTMS 2000 pulses over bilateral DLPFC	15	65.9±5.9	66.7	14.7±3.7	5	17	Egypt
		1Hz rTMS 2000 pulses over bilateral DLPFC	15	68.6±6.7	60.0	12.7±3.9			
		Sham control	15	68.3±4.9	66.7	13.9±3.9			
Cotelli, M. (2011)[89]	mild to moderate	20Hz rTMS 2000 pulses over left DLPFC	5	71.2±6.1	NA	16.2±2.7	2	2	Italy
		Sham control	5	74.4±3.8		16.0±2.0			

#: indicated "Treatment + post-treatment follow-up"

Abbreviation: AD: Alzheimer's disease; DLPFC: dorsolateral prefrontal cortex; IFG: inferior frontal gyrus; IPL: inferior parietal lobule; MMSE: mini-mental state examination; NA: not available; rTMS: repetitive transcranial magnetic stimulation; STG: superior temporal gyrus; tDCS: transcranial direct current stimulation; Tx: treatment

eTable 5A: League table of the changes of quality of life

HF-rTMS-F3F4			-0.63 (-1.36,0.10)		***-0.97 (-1.72,-0.23)**	
-0.28 (-1.82,1.25)	a-tDCS-F3 + c-tDCS-RtLb				-0.69 (-1.52,0.14)	
-0.32 (-1.82,1.18)	-0.04 (-1.58,1.51)	HF-rTMS-F3T3			-0.65 (-1.42,0.11)	
-0.63 (-1.67,0.41)	-0.35 (-1.87,1.17)	-0.31 (-1.80,1.18)	LF-rTMS-F3F4		-0.34 (-1.06,0.38)	
-0.70 (-2.13,0.72)	-0.42 (-1.89,1.05)	-0.38 (-1.82,1.05)	-0.07 (-1.48,1.34)	HF-rTMS-F3	-0.27 (-1.18,0.65)	
-0.97 (-2.03,0.08)	-0.69 (-1.80,0.42)	-0.65 (-1.72,0.42)	-0.34 (-1.38,0.70)	-0.27 (-1.23,0.69)	Sham	-0.19 (-0.98,0.60)
-1.16 (-2.68,0.35)	-0.88 (-2.44,0.68)	-0.85 (-2.37,0.68)	-0.53 (-2.04,0.97)	-0.46 (-1.91,0.99)	-0.19 (-1.28,0.90)	HF-rTMS-Mx

Pairwise (upper-right portion) and network (lower-left portion) meta-analysis results are presented as estimate effect sizes for the outcome of improvement of quality of life in patients with Alzheimer’s dementia. Interventions are reported in order of mean ranking of improvement of quality of life, and outcomes are expressed as standardized mean difference (SMD) (95% confidence intervals). For the pairwise meta-analyses, SMD of less than 0 indicate that the treatment specified in the row got more improvement than that specified in the column. For the network meta-analysis (NMA), SMD of less than 0 indicate that the treatment specified in the column got more improvement than that specified in the row. Bold results marked with * indicate statistical significance.

eTable 5B: League table of the rate of any adverse event

Sham	0.63 (0.12,3.31)	0.45 (0.10,2.11)	0.26 (0.06,1.21)	0.18 (0.01,4.03)	***0.04 (0.00,0.83)**
0.92 (0.17,4.80)	HF-rTMS-Mx	0.08 (0.00,1.67)			
0.31 (0.06,1.61)	0.33 (0.04,3.08)	HF-rTMS-F3			
0.26 (0.02,3.05)	0.29 (0.02,5.54)	0.86 (0.05,16.56)	a-tDCS-F3 + c-tDCS-Fp2		
0.18 (0.00,6.95)	0.20 (0.00,10.87)	0.60 (0.01,32.51)	0.69 (0.01,55.32)	a-tDCS-T3P3/T4P4 + c-tDCS-LtLb	
0.04 (0.00,1.46)	0.05 (0.00,2.30)	0.14 (0.00,6.89)	0.16 (0.00,11.80)	0.23 (0.00,36.97)	HF-rTMS-F3T3

Pairwise (upper-right portion) and network (lower-left portion) meta-analysis results are presented as estimate effect sizes for the outcome of rate of any adverse event in patients with Alzheimer's dementia. Interventions are reported in order of mean ranking of tolerability, and outcomes are expressed as odds ratio (OR) (95% confidence intervals). For the pairwise meta-analyses, OR of less than 1 indicate that the treatment specified in the row got better tolerability than that specified in the column. For the network meta-analysis (NMA), OR of less than 1 indicate that the treatment specified in the column got better tolerability than that specified in the row. Bold results marked with * indicate statistical significance.

eTable 5C: League table of the rate of local discomfort

HF-rTMS-Mx	0.64 (0.07,6.02)		0.08 (0.00,1.67)		
0.44 (0.05,4.14)	Sham	0.53 (0.11,2.60)	0.39 (0.03,5.85)	0.18 (0.01,4.03)	***0.04 (0.00,0.83)**
0.23 (0.01,8.52)	0.53 (0.03,8.89)	a-tDCS-F3 + c-tDCS-Fp2			
0.15 (0.01,2.04)	0.35 (0.05,2.58)	0.66 (0.02,20.92)	HF-rTMS-F3		
0.08 (0.00,7.02)	0.18 (0.00,8.78)	0.35 (0.00,41.57)	0.52 (0.01,40.86)	a-tDCS-T3P3/T4P4 + c-tDCS-LtLb	
0.02 (0.00,1.50)	0.04 (0.00,1.86)	0.08 (0.00,8.92)	0.12 (0.00,8.71)	0.23 (0.00,51.65)	HF-rTMS-F3T3

Pairwise (upper-right portion) and network (lower-left portion) meta-analysis results are presented as estimate effect sizes for the outcome of rate of local discomfort in patients with Alzheimer's dementia. Interventions are reported in order of mean ranking of tolerability, and outcomes are expressed as odds ratio (OR) (95% confidence intervals). For the pairwise meta-analyses, OR of less than 1 indicate that the treatment specified in the row got better tolerability than that specified in the column. For the network meta-analysis (NMA), OR of less than 1 indicate that the treatment specified in the column got better tolerability than that specified in the row. Bold results marked with * indicate statistical significance.

eTable 5D:League table of the drop-out rate

a-tDCS-T3P3/T4P4 + c-tDCS-LtLb				0.18 (0.01,4.03)			
0.50 (0.00,95.79)	HF-rTMS-F3			0.37 (0.01,10.18)			
0.29 (0.00,43.78)	0.58 (0.00,101.64)	a-tDCS-F3 + c-tDCS-F4		0.64 (0.03,11.91)			
0.21 (0.00,12.80)	0.43 (0.01,30.64)	0.74 (0.01,39.41)	HF-rTMS-Mx	0.88 (0.16,4.86)			
0.18 (0.00,6.84)	0.37 (0.01,16.75)	0.64 (0.02,20.74)	0.86 (0.13,5.92)	Sham	0.94 (0.20,4.29)	0.31 (0.01,8.33)	0.27 (0.02,3.10)
0.17 (0.00,13.39)	0.35 (0.00,31.76)	0.60 (0.01,41.56)	0.81 (0.04,17.87)	0.94 (0.08,10.58)	HF-rTMS-F3T3		
0.06 (0.00,10.71)	0.11 (0.00,24.72)	0.20 (0.00,33.89)	0.27 (0.00,18.76)	0.31 (0.01,13.71)	0.33 (0.00,29.68)	a-tDCS-T3 + c-tDCS-Fp2	
0.05 (0.00,5.77)	0.10 (0.00,13.51)	0.17 (0.00,18.09)	0.24 (0.01,8.86)	0.27 (0.01,5.90)	0.29 (0.01,14.59)	0.88 (0.01,116.70)	a-tDCS-F3 + c-tDCS-RtLb

Pairwise (upper-right portion) and network (lower-left portion) meta-analysis results are presented as estimate effect sizes for the outcome of drop-out rate in patients with Alzheimer’s dementia. Interventions are reported in order of mean ranking of tolerability, and outcomes are expressed as odds ratio (OR) (95% confidence intervals). For the pairwise meta-analyses, OR of less than 1 indicate that the treatment specified in the row got better tolerability than that specified in the column. For the network meta-analysis (NMA), OR of less than 1 indicate that the treatment specified in the column got better tolerability than that specified in the row. Bold results marked with * indicate statistical significance.

Abbreviation: AD: Alzheimer’s disease; ADAS-Cog: Alzheimer's disease assessment scale-cognitive subscale; a-tDCS-F3 + c-tDCS-F4: anodal tDCS of the left DLPFC and cathodal over the right DLPFC; a-tDCS-F3 + c-tDCS-Fp2: anodal tDCS of the left DLPFC and cathodal over right supraorbital region; a-tDCS-F3 + c-tDCS-RtLb: anodal tDCS of the left DLPFC and cathodal over the right deltoid muscle; a-tDCS-F7 + c-tDCS-Fp2: anodal tDCS of the left frontotemporal lobe and cathodal over right frontal lobe; a-tDCS-T3 + c-tDCS-Fp2: anodal tDCS of the left lateral temporal lobe and cathodal over right frontal lobe; a-tDCS-T3 + c-tDCS-RtLb: anodal tDCS of the left lateral temporal lobe and cathodal over right upper limb; a-tDCS-T3P3/T4P4 + c-tDCS-LtLb: anodal tDCS 2mA alternatively over the bilateral temporo-parietal lobe (T3-P3 or T4-P4) and cathodal over left arm deltoid muscle; CDR: clinical dementia rating; CI: confidence interval; c-tDCS-F3 + a-tDCS-Fp2: cathodal tDCS of the left DLPFC and anodal over right supraorbital region; DLPFC: dorsolateral prefrontal cortex; dTMS: deep TMS; HF-rTMS: high-frequency rTMS; HF-rTMS-F3: high frequency rTMS over left DLPFC; HF-rTMS-F3F4: high frequency rTMS over

bilateral DLPFC; HF-rTMS-F3T3: high frequency rTMS over left DLPFC and left lateral temporal lobe; HF-rTMS-F4: high frequency rTMS over right DLPFC; HF-rTMS-Mx: high frequency rTMS multifocal stimulation; IQR: interquartile range; LF-rTMS: low-frequency rTMS; LF-rTMS-F3F4: low frequency rTMS over bilateral DLPFC; MD: mean difference; MMSE: mini-mental state examination; NIBS: noninvasive brain stimulation; NMA: network meta-analysis; OR: odds ratio; PRISMA: preferred reporting items for systematic reviews and the meta-analysis; RCT: randomized controlled trial; rTMS: repetitive transcranial magnetic stimulation; Sham: sham control; SMD: standardized mean difference; SUCRA: surface under the cumulative ranking curve; TBS: theta-burst stimulation; tDCS: transcranial direct current stimulation

eTable 6A: SUCRA of the changes of cognition function-overall

Treatment	SUCRA
c-tDCS-F3 + a-tDCS-Fp2	92.9
HF-rTMS-F3F4	67.5
a-tDCS-F3 + c-tDCS-Fp2	67.0
a-tDCS-T3P3/T4P4 + c-tDCS-LtLb	65.1
a-tDCS-F7 + c-tDCS-Fp2	58.3
HF-rTMS-Mx	54.3
HF-rTMS-F3T3	52.3
HF-rTMS-F3	46.0
LF-rTMS-F3F4	39.7
a-tDCS-F3 + c-tDCS-F4	35.2
a-tDCS-T3 + c-tDCS-Fp2	30.6
Sham	22.3
a-tDCS-F3 + c-tDCS-RtLb	18.6

Sorted by efficacy order (the former, the better beneficial effect on cognition function)

eTable 6B: SUCRA of the changes of cognition function: measured with MMSE

Treatment	SUCRA
HF-rTMS-F3F4	92.5
c-tDCS-F3 + a-tDCS-Fp2	91.8
a-tDCS-F3 + c-tDCS-Fp2	81.9
a-tDCS-F7 + c-tDCS-Fp2	59.5
LF-rTMS-F3F4	52.6
HF-rTMS-F3T3	51.6
HF-rTMS-F3	36.9
a-tDCS-F3 + c-tDCS-F4	35.9
a-tDCS-T3 + c-tDCS-Fp2	34.6
HF-rTMS-Mx	26.4
Sham	24.9
a-tDCS-F3 + c-tDCS-RtLb	11.4

Sorted by efficacy order (the former, the better beneficial effect on cognition function)

eTable 6C: SUCRA of the changes of cognition function: measured with ADAS-Cog

Treatment	SUCRA
HF-rTMS-F3F4	23.5
HF-rTMS-F3	25.6
HF-rTMS-Mx	28.9
HF-rTMS-F3T3	48.3
Sham	83.3
a-tDCS-F3 + c-tDCS-Fp2	90.3

Sorted by efficacy order (the former, the better beneficial effect on cognition function)

eTable 6D: SUCRA of the changes of quality of life

Treatment	SUCRA
HF-rTMS-F3F4	18.7
a-tDCS-F3 + c-tDCS-RtLb	31.0
HF-rTMS-F3T3	35.0
LF-rTMS-F3F4	53.4
HF-rTMS-F3	55.5
Sham	76.6
HF-rTMS-Mx	79.8

Sorted by efficacy order (the former, the better improvement of quality of life)

eTable 6E: SUCRA of the rate of any adverse event

Treatment	SUCRA
Sham	81.6
HF-rTMS-Mx	76.0
HF-rTMS-F3	45.1
a-tDCS-F3 + c-tDCS-Fp2	44.3
a-tDCS-T3P3/T4P4 + c-tDCS-LtLb	37.8
HF-rTMS-F3T3	15.3

Sorted by efficacy order (the former, the less rate of any adverse event)

eTable 6F: SUCRA of the rate of local discomfort

Treatment	SUCRA
HF-rTMS-Mx	86.3
Sham	69.0
a-tDCS-F3 + c-tDCS-Fp2	53.9
HF-rTMS-F3	42.7
a-tDCS-T3P3/T4P4 + c-tDCS-LtLb	34.1
HF-rTMS-F3T3	14.0

Sorted by efficacy order (the former, the less rate of local discomfort)

eTable 6G: SUCRA of the drop-out rate

Treatment	SUCRA
a-tDCS-T3P3/T4P4 + c-tDCS-LtLb	76.1
HF-rTMS-F3	66.6
a-tDCS-F3 + c-tDCS-F4	59.4
HF-rTMS-Mx	52.2
Sham	47.8
HF-rTMS-F3T3	46.2
a-tDCS-T3 + c-tDCS-Fp2	27.0
a-tDCS-F3 + c-tDCS-RtLb	24.7

Sorted by efficacy order (the former, the less drop-out rate)

Abbreviation: AD: Alzheimer's disease; ADAS-Cog: Alzheimer's disease assessment scale-cognitive subscale; a-tDCS-F3 + c-tDCS-F4: anodal tDCS of the left DLPFC and cathodal over the right DLPFC; a-tDCS-F3 + c-tDCS-Fp2: anodal tDCS of the left DLPFC and cathodal over right supraorbital region; a-tDCS-F3 + c-tDCS-RtLb: anodal tDCS of the left DLPFC and cathodal over the right deltoid muscle; a-tDCS-F7 + c-tDCS-Fp2: anodal tDCS of the left frontotemporal lobe and cathodal over right frontal lobe; a-tDCS-T3 + c-tDCS-Fp2: anodal tDCS of the left lateral temporal lobe and cathodal over right frontal lobe; a-tDCS-T3 + c-tDCS-RtLb: anodal tDCS of the left lateral temporal lobe and cathodal over right upper limb; a-tDCS-T3P3/T4P4 + c-tDCS-LtLb: anodal tDCS 2mA alternatively over the bilateral temporo-parietal lobe (T3-P3 or T4-P4) and cathodal over left arm deltoid muscle; CDR: clinical dementia rating; CI: confidence interval; c-tDCS-F3 + a-tDCS-Fp2: cathodal tDCS of the left DLPFC and anodal over right supraorbital region; DLPFC: dorsolateral prefrontal cortex; dTMS: deep TMS; HF-rTMS: high-frequency rTMS; HF-rTMS-F3: high frequency rTMS over left DLPFC; HF-rTMS-F3F4: high frequency rTMS over bilateral DLPFC; HF-rTMS-F3T3: high frequency rTMS over left DLPFC

and left lateral temporal lobe; HF-rTMS-F4: high frequency rTMS over right DLPFC; HF-rTMS-Mx: high frequency rTMS multifocal stimulation; IQR: interquartile range; LF-rTMS: low-frequency rTMS; LF-rTMS-F3F4: low frequency rTMS over bilateral DLPFC; MD: mean difference; MMSE: mini-mental state examination; NIBS: noninvasive brain stimulation; NMA: network meta-analysis; OR: odds ratio; PRISMA: preferred reporting items for systematic reviews and the meta-analysis; RCT: randomized controlled trial; rTMS: repetitive transcranial magnetic stimulation; Sham: sham control; SMD: standardized mean difference; SUCRA: surface under the cumulative ranking curve; TBS: theta-burst stimulation; tDCS: transcranial direct current stimulation

eTable 7: Inconsistency of different intervention

Part 1: design-by-treatment and loop inconsistency model

Inconsistency model	chi^2	Prob>chi^2
	Improvement of cognitive function: overall	
design-by-treatment	10.86	0.0125
loop inconsistency	0.36	0.5459
	Improvement of cognitive function: measurement with MMSE	
design-by-treatment	0.15	0.7001
loop inconsistency	0.15	0.7001
	Improvement of cognitive function: measurement with ADAS-Cog	
design-by-treatment	0.01	0.9044
loop inconsistency	0.01	0.9044
	Change of quality of life	
design-by-treatment	1.41	0.2354
loop inconsistency	1.41	0.2354
	Safety profile: rate of any adverse event	
design-by-treatment	1.09	0.2970
loop inconsistency	1.09	0.2970
	Safety profile: rate of local discomfort	
design-by-treatment	0.16	0.6937
loop inconsistency	0.16	0.6937
	Acceptability: drop-out rate	

design-by-treatment	0.26	0.6082
loop inconsistency	0.26	0.6082

Part 2: side-splitting inconsistency model:

Part of improvement of cognitive function: overall

Side	symmetric		nosymmetric		Treatments used	
	P>z	tau	P>z	tau		
A B	.	.	.	.	A (reference):	Sham
A C *	0	0.418386	.	.	B:	a-tDCS-F3 + c-tDCS-Fp2
A D	0.546	0.86479	0.546	0.86479	C:	c-tDCS-F3 + a-tDCS-Fp2
A E	0.546	0.86479	0.546	0.86479	D:	HF-rTMS-F3
A F	.	.	.	.	E:	HF-rTMS-Mx
A G	.	.	.	.	F:	a-tDCS-F3 + c-tDCS-F4
A H *	0.887	0.874469	.	.	G:	HF-rTMS-F3F4
A I	.	.	.	.	H:	LF-rTMS-F3F4
A J	.	.	.	.	I:	a-tDCS-F7 + c-tDCS-Fp2
A K	.	.	.	.	J:	HF-rTMS-F3T3
A L	.	.	.	.	K:	a-tDCS-F3 + c-tDCS-RtLb
A M	.	.	.	.	L:	a-tDCS-T3P3/T4P4 + c-tDCS-LtLb

B C *	0	0.418386	.	.	M:	a-tDCS-T3 + c-tDCS-Fp2
D E	0.546	0.86479	0.546	0.864791		
G H *	0.887	0.874469	.	.		

Part of improvement of cognitive function: measurement with MMSE

Side	symmetric		nosymmetric		Treatments used	
	P>z	tau	P>z	tau		
A B	.	.	.	.	A (reference):	Sham
A C	.	.	.	.	B:	a-tDCS-F3 + c-tDCS-Fp2
A D	0.7	0.842798	0.7	0.842798	C:	c-tDCS-F3 + a-tDCS-Fp2
A E	0.7	0.842799	0.7	0.842799	D:	HF-rTMS-F3
A F	.	.	.	.	E:	HF-rTMS-Mx
A G	.	.	.	.	F:	a-tDCS-F3 + c-tDCS-F4
A H	.	.	.	.	G:	HF-rTMS-F3F4
A I	.	.	.	.	H:	LF-rTMS-F3F4
A J	.	.	.	.	I:	a-tDCS-F7 + c-tDCS-Fp2
A K	.	.	.	.	J:	HF-rTMS-F3T3
A L	.	.	.	.	K:	a-tDCS-F3 + c-tDCS-RtLb
B C	.	.	.	.	L:	a-tDCS-T3 + c-tDCS-Fp2
D E	0.7	0.842798	0.7	0.842799		
G H	.	.	.	.		

Part of improvement of cognitive function: measurement with ADAS-Cog

Side	symmetric		nosymmetric		Treatments used	
	P>z	tau	P>z	tau		
A B	.	.	.	.	A (reference):	Sham
A C	.	.	.	.	B:	a-tDCS-F3 + c-tDCS-Fp2
A D	0.904	1.078794	0.904	1.078794	C:	HF-rTMS-F3T3
A E	0.904	1.078793	0.904	1.078793	D:	HF-rTMS-F3
A F	.	.	.	.	E:	HF-rTMS-Mx
D E	0.904	1.078793	0.904	1.078798	F:	HF-rTMS-F3F4

Part of change of quality of life

Side	symmetric		nosymmetric		Treatments used	
	P>z	tau	P>z	tau		
A B	.	.	.	.	A (reference):	Sham
A C	.	.	.	.	B:	a-tDCS-F3 + c-tDCS-RtLb
A D	.	.	.	.	C:	HF-rTMS-F3T3
A E	.	.	.	.	D:	HF-rTMS-F3
A F	.	.	.	.	E:	HF-rTMS-Mx

A G	.	.	.	.	F:	LF-rTMS-F3F4
F G	.	.	.	.	G:	HF-rTMS-F3F4

Part of safety profile: rate of any adverse event

Side	symmetric		nosymmetric		Treatments used	
	P>z	tau	P>z	tau		
A B	.	.	.	.	A (reference):	Sham
A C	.	.	.	.	B:	a-tDCS-F3 + c-tDCS-Fp2
A D	0.297	0.798316	0.297	0.798316	C:	a-tDCS-T3P3/T4P4 + c-tDCS-LtLb
A E	0.297	0.798314	0.297	0.798314	D:	HF-rTMS-F3
A F	.	.	.	.	E:	HF-rTMS-Mx
D E	0.297	0.798315	0.297	0.798314	F:	HF-rTMS-F3T3

Part of safety profile: rate of local discomfort

Side	symmetric		nosymmetric		Treatments used	
	P>z	tau	P>z	tau		
A B	.	.	.	.	A (reference):	Sham
A C	.	.	.	.	B:	a-tDCS-F3 + c-tDCS-Fp2
A D	0.694	1.433658	0.694	1.433658	C:	a-tDCS-T3P3/T4P4 + c-

						tDCS-LtLb
A E	0.694	1.433658	0.694	1.433658	D:	HF-rTMS-F3
A F	.	.	.	.	E:	HF-rTMS-Mx
D E	0.694	1.433658	0.694	1.433657	F:	HF-rTMS-F3T3

Part of acceptability: drop-out rate

Side	symmetric		nosymmetric		Treatments used	
	P>z	tau	P>z	tau		
A B	.	.	.	.	A (reference):	Sham
A C	.	.	.	.	B:	a-tDCS-F3 + c-tDCS-RtLb
A D	.	.	.	.	C:	a-tDCS-T3 + c-tDCS-Fp2
A E	.	.	.	.	D:	a-tDCS-T3P3/T4P4 + c-tDCS-LtLb
A F	.	.	.	.	E:	HF-rTMS-Mx
A G	.	.	.	.	F:	a-tDCS-F3 + c-tDCS-F4
A H	.	.	.	.	G:	HF-rTMS-F3T3
					H:	HF-rTMS-F3

Abbreviation: ADAS-Cog: Alzheimer's disease assessment scale-cognitive subscale; a-tDCS-F3 + c-tDCS-F4: anodal tDCS of the left DLPFC and cathodal over the right DLPFC; a-tDCS-F3 + c-tDCS-Fp2: anodal tDCS of the left DLPFC and cathodal over right supraorbital region; a-tDCS-F3 + c-tDCS-RtLb: anodal tDCS of the left DLPFC and cathodal over the right deltoid muscle; a-tDCS-F7 + c-tDCS-Fp2: anodal tDCS of the left frontotemporal lobe and cathodal over right frontal lobe; a-tDCS-T3 + c-tDCS-Fp2: anodal tDCS of the

left lateral temporal lobe and cathodal over right frontal lobe; a-tDCS-T3 + c-tDCS-RtLb: anodal tDCS of the left lateral temporal lobe and cathodal over right upper limb; a-tDCS-T3P3/T4P4 + c-tDCS-LtLb: anodal tDCS 2mA alternatively over the bilateral temporo-parietal lobe (T3-P3 or T4-P4) and cathodal over left arm deltoid muscle; CI: confidence interval; c-tDCS-F3 + a-tDCS-Fp2: cathodal tDCS of the left DLPFC and anodal over right supraorbital region; DLPFC: dorsolateral prefrontal cortex; HF-rTMS: high-frequency rTMS; HF-rTMS-F3: high frequency rTMS over left DLPFC; HF-rTMS-F3F4: high frequency rTMS over bilateral DLPFC; HF-rTMS-F3T3: high frequency rTMS over left DLPFC and left lateral temporal lobe; HF-rTMS-F4: high frequency rTMS over right DLPFC; HF-rTMS-Mx: high frequency rTMS multifocal stimulation; IQR: interquartile range; LF-rTMS: low-frequency rTMS; LF-rTMS-F3F4: low frequency rTMS over bilateral DLPFC; MD: mean difference; MMSE: mini-mental state examination; NIBS: noninvasive brain stimulation; NMA: network meta-analysis; OR: odds ratio; rTMS: repetitive transcranial magnetic stimulation; Sham: sham control; SMD: standardized mean difference; SUCRA: surface under the cumulative ranking curve; tDCS: transcranial direct current stimulation

eTable 8: Estimated between-studies standard deviation of different outcome

Outcome	Estimated between-studies standard deviation
Improvement of cognitive function: overall	0.82055962
Improvement of cognitive function: measurement with MMSE	0.67268911
Improvement of cognitive function: measurement with ADAS-Cog	1.0263506
Change of quality of life	0.38079079
Safety profile: rate of any adverse event	0.97720328
Safety profile: rate of local discomfort	1.1883289
Acceptability: drop-out rate	0.96217797

eTable 9: GRADE evaluation quality of evidence for primary outcome

We evaluated the quality of evidence followed the articles of GRADE Working Group [90] and of Cipriani, A [91].

Comparisons (study number)	GRADE					
	Direct		Indirect		Network meta-analysis	
	SMD (95% CI)	The final rating of direct evidence	Co-efficient (SE)	The final rating of indirect evidence	SMD (95% CI)	Overall quality of evidence
c-tDCS-F3 + a-tDCS-Fp2 vs HF-rTMS-F3F4					1.31 (-0.97,3.59)	⊕◯◯◯ Very low
c-tDCS-F3 + a-tDCS-Fp2 vs a-tDCS-F3 + c-tDCS-Fp2	0.59 (-0.24,1.42)	⊕⊕◯◯ Low	6.85 (1.69)	⊕⊕◯◯ Low	1.30 (-0.42,3.01)	⊕⊕⊕◯ Medium
c-tDCS-F3 + a-tDCS-Fp2 vs a-tDCS-T3P3/T4P4 + c-tDCS-LtLb					1.31 (-1.21,3.83)	⊕◯◯◯ Very low
c-tDCS-F3 + a-tDCS-Fp2 vs a-tDCS-F7 + c-tDCS-Fp2					1.59 (-0.65,3.83)	⊕◯◯◯ Very low
c-tDCS-F3 + a-tDCS-Fp2 vs HF-rTMS-Mx					1.71 (-0.26,3.69)	⊕◯◯◯ Very low
c-tDCS-F3 + a-tDCS-Fp2 vs HF-rTMS-F3T3					1.71 (-0.85,4.26)	⊕◯◯◯ Very low
c-tDCS-F3 + a-tDCS-Fp2 vs HF-rTMS-F3					1.93 (-0.13,3.99)	⊕◯◯◯ Very low
c-tDCS-F3 + a-tDCS-Fp2 vs LF-rTMS-F3F4					2.09 (-0.39,4.56)	⊕◯◯◯ Very low
c-tDCS-F3 + a-tDCS-Fp2 vs a-tDCS-F3 + c-tDCS-F4					2.31 (-0.31,4.92)	⊕◯◯◯ Very low

c-tDCS-F3 + a-tDCS-Fp2 vs a-tDCS-T3 + c-tDCS-Fp2					2.43 (-0.12,4.99)	⊕◯◯◯ Very low
c-tDCS-F3 + a-tDCS-Fp2 vs Sham	***3.50 (2.28,4.71)**	⊕⊕⊕◯ Medium	-2.77 (1.50)	⊕⊕◯◯ Low	***2.43 (0.61,4.26)**	⊕⊕⊕⊕ High
c-tDCS-F3 + a-tDCS-Fp2 vs a-tDCS-F3 + c-tDCS-RtLb					***2.88 (0.31,5.45)**	⊕⊕◯◯ Low
HF-rTMS-F3F4 vs a-tDCS-F3 + c-tDCS-Fp2					-0.02 (-1.91,1.87)	⊕⊕◯◯ Low
HF-rTMS-F3F4 vs a-tDCS-T3P3/T4P4 + c-tDCS-LtLb					0.00 (-2.20,2.20)	⊕◯◯◯ Very low
HF-rTMS-F3F4 vs a-tDCS-F7 + c-tDCS-Fp2					0.28 (-1.61,2.17)	⊕◯◯◯ Very low
HF-rTMS-F3F4 vs HF-rTMS-Mx					0.40 (-1.16,1.97)	⊕⊕◯◯ Low
HF-rTMS-F3F4 vs HF-rTMS-F3T3					0.40 (-1.85,2.64)	⊕◯◯◯ Very low
HF-rTMS-F3F4 vs HF-rTMS-F3					0.62 (-1.04,2.27)	⊕◯◯◯ Very low
HF-rTMS-F3F4 vs LF-rTMS-F3F4	***0.82 (0.08,1.56)**	⊕⊕⊕◯ Medium	-0.40 (2.79)	⊕◯◯◯ Very low	0.78 (-0.90,2.45)	⊕⊕⊕⊕ High
HF-rTMS-F3F4 vs a-tDCS-F3 + c-tDCS-F4					1.00 (-1.32,3.31)	⊕◯◯◯ Very low
HF-rTMS-F3F4 vs a-tDCS-T3 + c-tDCS-Fp2					1.12 (-1.13,3.37)	⊕◯◯◯ Very low
HF-rTMS-F3F4 vs Sham	***1.16 (0.49,1.83)**	⊕⊕⊕◯ Medium			1.12 (-0.24,2.49)	⊕⊕⊕◯ Medium
HF-rTMS-F3F4 vs a-tDCS-F3 + c-tDCS-RtLb					1.57 (-0.69,3.83)	⊕◯◯◯ Very low
a-tDCS-F3 + c-tDCS-Fp2 vs a-tDCS-T3P3/T4P4 + c-tDCS-LtLb					0.02 (-2.15,2.19)	⊕◯◯◯ Very low
a-tDCS-F3 + c-tDCS-Fp2 vs a-tDCS-F7 + c-tDCS-Fp2					0.30 (-1.55,2.14)	⊕◯◯◯ Very low

a-tDCS-F3 + c-tDCS-Fp2 vs HF-rTMS-Mx			0.42 (-1.09,1.93)	⊕⊕◯◯ Low
a-tDCS-F3 + c-tDCS-Fp2 vs HF-rTMS-F3T3			0.41 (-1.80,2.62)	⊕◯◯◯ Very low
a-tDCS-F3 + c-tDCS-Fp2 vs HF-rTMS-F3			0.63 (-0.98,2.24)	⊕◯◯◯ Very low
a-tDCS-F3 + c-tDCS-Fp2 vs LF-rTMS-F3F4			0.79 (-1.33,2.91)	⊕◯◯◯ Very low
a-tDCS-F3 + c-tDCS-Fp2 vs a-tDCS-F3 + c-tDCS-F4			1.01 (-1.27,3.29)	⊕◯◯◯ Very low
a-tDCS-F3 + c-tDCS-Fp2 vs a-tDCS-T3 + c-tDCS-Fp2			1.14 (-1.08,3.36)	⊕◯◯◯ Very low
a-tDCS-F3 + c-tDCS-Fp2 vs Sham	1.30 (-1.77,4.37)	⊕◯◯◯ Very low	1.14 (-0.17,2.45)	⊕⊕◯◯ Low
a-tDCS-F3 + c-tDCS-Fp2 vs a-tDCS-F3 + c-tDCS-RtLb			1.58 (-0.64,3.81)	⊕◯◯◯ Very low
a-tDCS-T3P3/T4P4 + c-tDCS-LtLb vs a-tDCS-F7 + c-tDCS-Fp2			0.28 (-1.89,2.45)	⊕◯◯◯ Very low
a-tDCS-T3P3/T4P4 + c-tDCS-LtLb vs HF-rTMS-Mx			0.40 (-1.49,2.29)	⊕◯◯◯ Very low
a-tDCS-T3P3/T4P4 + c-tDCS-LtLb vs HF-rTMS-F3T3			0.39 (-2.09,2.88)	⊕◯◯◯ Very low
a-tDCS-T3P3/T4P4 + c-tDCS-LtLb vs HF-rTMS-F3			0.62 (-1.35,2.59)	⊕◯◯◯ Very low
a-tDCS-T3P3/T4P4 + c-tDCS-LtLb vs LF-rTMS-F3F4			0.77 (-1.63,3.18)	⊕◯◯◯ Very low
a-tDCS-T3P3/T4P4 + c-tDCS-LtLb vs a-			0.99 (-1.55,3.54)	⊕◯◯◯ Very low

tDCS-F3 + c-tDCS-F4						
a-tDCS-T3P3/T4P4 + c-tDCS-LtLb vs a-tDCS-T3 + c-tDCS-Fp2					1.12 (-1.37,3.61)	⊕◯◯◯ Very low
a-tDCS-T3P3/T4P4 + c-tDCS-LtLb vs Sham	***1.12 (0.48,1.76)**	⊕⊕⊕◯ Medium			1.12 (-0.61,2.85)	⊕⊕⊕◯ Medium
a-tDCS-T3P3/T4P4 + c-tDCS-LtLb vs a-tDCS-F3 + c-tDCS-RtLb					1.57 (-0.93,4.07)	⊕◯◯◯ Very low
a-tDCS-F7 + c-tDCS-Fp2 vs HF-rTMS-Mx					0.12 (-1.39,1.64)	⊕⊕◯◯ Low
a-tDCS-F7 + c-tDCS-Fp2 vs HF-rTMS-F3T3					0.12 (-2.10,2.33)	⊕◯◯◯ Very low
a-tDCS-F7 + c-tDCS-Fp2 vs HF-rTMS-F3					0.34 (-1.27,1.95)	⊕⊕◯◯ Low
a-tDCS-F7 + c-tDCS-Fp2 vs LF-rTMS-F3F4					0.50 (-1.62,2.62)	⊕◯◯◯ Very low
a-tDCS-F7 + c-tDCS-Fp2 vs a-tDCS-F3 + c-tDCS-F4					0.72 (-1.56,3.00)	⊕◯◯◯ Very low
a-tDCS-F7 + c-tDCS-Fp2 vs a-tDCS-T3 + c-tDCS-Fp2					0.84 (-1.37,3.06)	⊕◯◯◯ Very low
a-tDCS-F7 + c-tDCS-Fp2 vs Sham	0.81 (-0.07,1.68)	⊕⊕◯◯ Low			0.84 (-0.46,2.15)	⊕⊕◯◯ Low
a-tDCS-F7 + c-tDCS-Fp2 vs a-tDCS-F3 + c-tDCS-RtLb					1.29 (-0.94,3.51)	⊕◯◯◯ Very low
HF-rTMS-Mx vs HF-rTMS-F3T3					-0.01 (-1.95,1.93)	⊕◯◯◯ Very low
HF-rTMS-Mx vs HF-rTMS-F3	-0.26 (-1.17,0.64)	⊕⊕◯◯ Low	0.47 (0.71)	⊕⊕⊕◯ Medium	0.21 (-0.87,1.30)	⊕⊕⊕⊕ High
HF-rTMS-Mx vs LF-rTMS-F3F4					0.37 (-1.46,2.20)	⊕◯◯◯ Very low
HF-rTMS-Mx vs a-tDCS-F3 + c-tDCS-F4					0.59 (-1.42,2.61)	⊕◯◯◯ Very low
HF-rTMS-Mx vs a-tDCS-T3 + c-tDCS-Fp2					0.72 (-1.22,2.66)	⊕◯◯◯ Very low

HF-rTMS-Mx vs Sham	***0.78 (0.15,1.42)**	⊕⊕⊕◯ Medium	0.08 (1.13)	⊕⊕◯◯ Low	0.72 (-0.04,1.48)	⊕⊕⊕⊕ High
HF-rTMS-Mx vs a-tDCS-F3 + c-tDCS-RtLb					1.16 (-0.79,3.12)	⊕◯◯◯ Very low
HF-rTMS-F3T3 vs HF-rTMS-F3					0.22 (-1.79,2.24)	⊕◯◯◯ Very low
HF-rTMS-F3T3 vs LF-rTMS-F3F4					0.38 (-2.06,2.82)	⊕◯◯◯ Very low
HF-rTMS-F3T3 vs a-tDCS-F3 + c-tDCS-F4					0.60 (-1.98,3.18)	⊕◯◯◯ Very low
HF-rTMS-F3T3 vs a-tDCS-T3 + c-tDCS-Fp2					0.73 (-1.80,3.25)	⊕◯◯◯ Very low
HF-rTMS-F3T3 vs Sham	0.73 (-0.04,1.50)	⊕⊕◯◯ Low			0.73 (-1.05,2.51)	⊕⊕◯◯ Low
HF-rTMS-F3T3 vs a-tDCS-F3 + c-tDCS-RtLb					1.17 (-1.36,3.71)	⊕◯◯◯ Very low
HF-rTMS-F3 vs LF-rTMS-F3F4					0.16 (-1.76,2.07)	⊕◯◯◯ Very low
HF-rTMS-F3 vs a-tDCS-F3 + c-tDCS-F4					0.38 (-1.71,2.47)	⊕◯◯◯ Very low
HF-rTMS-F3 vs a-tDCS-T3 + c-tDCS-Fp2					0.51 (-1.52,2.53)	⊕◯◯◯ Very low
HF-rTMS-F3 vs Sham	***0.48 (0.04,0.92)**	⊕⊕⊕◯ Medium	1.08 (1.07)	⊕⊕◯◯ Low	0.51 (-0.43,1.45)	⊕⊕⊕⊕ High
HF-rTMS-F3 vs a-tDCS-F3 + c-tDCS-RtLb					0.95 (-1.08,2.98)	⊕◯◯◯ Very low
LF-rTMS-F3F4 vs a-tDCS-F3 + c-tDCS-F4					0.22 (-2.28,2.72)	⊕◯◯◯ Very low
LF-rTMS-F3F4 vs a-tDCS-T3 + c-tDCS-Fp2					0.35 (-2.10,2.79)	⊕◯◯◯ Very low
LF-rTMS-F3F4 vs Sham	0.39 (-0.33,1.11)	⊕⊕◯◯ Low	-0.03 (2.79)	⊕◯◯◯ Very low	0.35 (-1.32,2.02)	⊕⊕⊕◯ Medium
LF-rTMS-F3F4 vs a-tDCS-F3 + c-tDCS-RtLb					0.79 (-1.66,3.25)	⊕◯◯◯ Very low
a-tDCS-F3 + c-tDCS-F4 vs a-tDCS-T3 + c-tDCS-Fp2					0.13 (-2.46,2.72)	⊕◯◯◯ Very low
a-tDCS-F3 + c-tDCS-F4 vs Sham	0.13 (-0.82,1.08)	⊕⊕◯◯ Low			0.13 (-1.74,2.00)	⊕⊕◯◯ Low
a-tDCS-F3 + c-tDCS-F4 vs a-tDCS-F3 + c-					0.57 (-2.02,3.17)	⊕◯◯◯ Very low

tDCS-RtLb				
a-tDCS-T3 + c-tDCS-Fp2 vs Sham	0.00 (-0.79,0.79)	⊕⊕◯◯ Low	-0.00 (-1.79,1.79)	⊕⊕◯◯ Low
a-tDCS-T3 + c-tDCS-Fp2 vs a-tDCS-F3 + c-tDCS-RtLb			0.44 (-2.09,2.98)	⊕◯◯◯ Very low
Sham vs a-tDCS-F3 + c-tDCS-RtLb	0.44 (-0.37,1.26)	⊕⊕◯◯ Low	0.44 (-1.36,2.25)	⊕⊕◯◯ Low

Abbreviation: CI: confidence interval; SE: standard error; SMD: standardized mean difference

Reference of the supplement tables:

1. Page MJ, McKenzie JE, Bossuyt PM, Boutron I, Hoffmann TC, Mulrow CD, et al.: The PRISMA 2020 statement: an updated guideline for reporting systematic reviews. Bmj. 2021; 372:n71.
2. Benussi A, Grassi M, Palluzzi F, Koch G, Di Lazzaro V, Nardone R, et al.: Classification Accuracy of Transcranial Magnetic Stimulation for the Diagnosis of Neurodegenerative Dementias. Ann Neurol. 2020; 87(3):394-404.
3. Kumar S, Zomorrodi R, Ghazala Z, Goodman MS, Blumberger DM, Daskalakis ZJ, et al.: Effects of repetitive paired associative stimulation on brain plasticity and working memory in Alzheimer's disease: a pilot randomized double-blind-controlled trial. Int Psychogeriatr. 2020:1-13.
4. Wang X, Mao Z, Yu X: The role of noninvasive brain stimulation for behavioral and psychological symptoms of dementia: a systematic review and meta-analysis. Neurol Sci. 2020; 41(5):1063-74.
5. Chou YH, Ton That V, Sundman M: A systematic review and meta-analysis of rTMS effects on cognitive enhancement in mild cognitive impairment and Alzheimer's disease. Neurobiol Aging. 2020; 86:1-10.
6. Wang X, Mao Z, Ling Z, Yu X: Repetitive transcranial magnetic stimulation for cognitive impairment in Alzheimer's disease: a meta-analysis of randomized controlled trials. J Neurol. 2020; 267(3):791-801.

7. Lin Y, Jiang WJ, Shan PY, Lu M, Wang T, Li RH, et al.: The role of repetitive transcranial magnetic stimulation (rTMS) in the treatment of cognitive impairment in patients with Alzheimer's disease: A systematic review and meta-analysis. J Neurol Sci. 2019; 398:184-91.
8. Xu Y, Qiu Z, Zhu J, Liu J, Wu J, Tao J, et al.: The modulation effect of non-invasive brain stimulation on cognitive function in patients with mild cognitive impairment: a systematic review and meta-analysis of randomized controlled trials. BMC neuroscience. 2019; 20(1):2.
9. Cruz Gonzalez P, Fong KNK, Chung RCK, Ting KH, Law LLF, Brown T: Can Transcranial Direct-Current Stimulation Alone or Combined With Cognitive Training Be Used as a Clinical Intervention to Improve Cognitive Functioning in Persons With Mild Cognitive Impairment and Dementia? A Systematic Review and Meta-Analysis. Front Hum Neurosci. 2018; 12:416.
10. Dong X, Yan L, Huang L, Guan X, Dong C, Tao H, et al.: Repetitive transcranial magnetic stimulation for the treatment of Alzheimer's disease: A systematic review and meta-analysis of randomized controlled trials. PloS one. 2018; 13(10):e0205704.
11. Vacas SM, Stella F, Loureiro JC, Simoes do Couto F, Oliveira-Maia AJ, Forlenza OV: Noninvasive brain stimulation for behavioural and psychological symptoms of dementia: A systematic review and meta-analysis. Int J Geriatr Psychiatry. 2019; 34(9):1336-45.
12. May BH, Feng M, Hyde AJ, Hugel H, Chang SY, Dong L, et al.: Comparisons between traditional medicines and pharmacotherapies for Alzheimer disease: A systematic review and meta-analysis of cognitive outcomes. Int J Geriatr Psychiatry. 2018; 33(3):449-58.
13. Liao X, Li G, Wang A, Liu T, Feng S, Guo Z, et al.: Repetitive Transcranial Magnetic Stimulation as an Alternative Therapy for Cognitive Impairment in Alzheimer's Disease: A Meta-Analysis. J Alzheimers Dis. 2015; 48(2):463-72.
14. Cai M, Guo Z, Xing G, Peng H, Zhou L, Chen H, et al.: Transcranial Direct Current Stimulation Improves Cognitive Function in Mild to Moderate Alzheimer Disease: A Meta-Analysis. Alzheimer disease and associated disorders. 2019; 33(2):170-8.
15. Yuan LQ, Zeng Q, Wang D, Wen XY, Shi Y, Zhu F, et al.: Neuroimaging mechanisms of high-frequency repetitive transcranial magnetic stimulation for treatment of amnestic mild cognitive impairment: a double-blind randomized sham-controlled trial. Neural Regen Res. 2021; 16(4):707-13.
16. Lu H, Chan SSM, Chan WC, Lin C, Cheng CPW, Linda Chiu Wa L: Randomized controlled trial of TDCS on cognition in 201 seniors with mild neurocognitive disorder. Ann Clin Transl Neurol. 2019; 6(10):1938-48.
17. Cui H, Ren R, Lin G, Zou Y, Jiang L, Wei Z, et al.: Repetitive Transcranial Magnetic Stimulation Induced Hypoconnectivity Within the

Default Mode Network Yields Cognitive Improvements in Amnestic Mild Cognitive Impairment: A Randomized Controlled Study. J Alzheimers Dis. 2019; 69(4):1137-51.
18. Chu CS, Li CT, Brunoni AR, Yang FC, Tseng PT, Tu YK, et al.: Cognitive effects and acceptability of non-invasive brain stimulation on Alzheimer's disease and mild cognitive impairment: a component network meta-analysis. Journal of neurology, neurosurgery, and psychiatry. 2020.
19. Park HK, Song MK, Kim JH, Han JY: A randomized controlled trial to evaluate the effectiveness and safety of electro acupuncture and transcranial direct current stimulation with computerized cognitive rehabilitation in patients with vascular cognitive impairment. Medicine. 2020; 99(29):e21263.
20. Nissim NR, O'Shea A, Indahlastari A, Kraft JN, von Mering O, Aksu S, et al.: Effects of Transcranial Direct Current Stimulation Paired With Cognitive Training on Functional Connectivity of the Working Memory Network in Older Adults. Front Aging Neurosci. 2019; 11:340.
21. Martin DM, Mohan A, Alonzo A, Gates N, Gbadeyan O, Meinzer M, et al.: A Pilot Double-Blind Randomized Controlled Trial of Cognitive Training Combined with Transcranial Direct Current Stimulation for Amnestic Mild Cognitive Impairment. J Alzheimers Dis. 2019; 71(2):503-12.
22. Lau CI, Liu MN, Chang KC, Chang A, Bai CH, Tseng CS, et al.: Effect of single-session transcranial direct current stimulation on cognition in Parkinson's disease. CNS neuroscience & therapeutics. 2019; 25(11):1237-43.
23. Das N, Spence JS, Aslan S, Vanneste S, Mudar R, Rackley A, et al.: Cognitive Training and Transcranial Direct Current Stimulation in Mild Cognitive Impairment: A Randomized Pilot Trial. Front Neurosci. 2019; 13:307.
24. Pelosin E, Cerulli C, Ogliastro C, Lagravinese G, Mori L, Bonassi G, et al.: A Multimodal Training Modulates Short Afferent Inhibition and Improves Complex Walking in a Cohort of Faller Older Adults With an Increased Prevalence of Parkinson's Disease. J Gerontol A Biol Sci Med Sci. 2020; 75(4):722-8.
25. McCall WV, Lisanby SH, Rosenquist PB, Dooley M, Husain MM, Knapp RG, et al.: Effects of continuation electroconvulsive therapy on quality of life in elderly depressed patients: A randomized clinical trial. Journal of psychiatric research. 2018; 97:65-9.
26. Hampstead BM, Sathian K, Bikson M, Stringer AY: Combined mnemonic strategy training and high-definition transcranial direct current

stimulation for memory deficits in mild cognitive impairment. Alzheimers Dement (N Y). 2017; 3(3):459-70.
27. Murugaraja V, Shivakumar V, Sivakumar PT, Sinha P, Venkatasubramanian G: Clinical utility and tolerability of transcranial direct current stimulation in mild cognitive impairment. Asian J Psychiatr. 2017; 30:135-40.
28. Yun K, Song IU, Chung YA: Changes in cerebral glucose metabolism after 3 weeks of noninvasive electrical stimulation of mild cognitive impairment patients. Alzheimers Res Ther. 2016; 8(1):49.
29. Benussi A, Dell'Era V, Cosseddu M, Cantoni V, Cotelli MS, Cotelli M, et al.: Transcranial stimulation in frontotemporal dementia: A randomized, double-blind, sham-controlled trial. Alzheimers Dement (N Y). 2020; 6(1):e12033.
30. Inagawa T, Yokoi Y, Narita Z, Maruo K, Okazaki M, Nakagome K: Safety and Feasibility of Transcranial Direct Current Stimulation for Cognitive Rehabilitation in Patients With Mild or Major Neurocognitive Disorders: A Randomized Sham-Controlled Pilot Study. Front Hum Neurosci. 2019; 13:273.
31. Vatanparasti S, Kazemnejad A, Yoonessi A, Oveisgharan S: The Effect of Continuous Theta-Burst Transcranial Magnetic Stimulation Combined with Prism Adaptation on the Neglect Recovery in Stroke Patients. J Stroke Cerebrovasc Dis. 2019; 28(11):104296.
32. Trung J, Hanganu A, Jobert S, Degroot C, Mejia-Constain B, Kibreab M, et al.: Transcranial magnetic stimulation improves cognition over time in Parkinson's disease. Parkinsonism Relat Disord. 2019; 66:3-8.
33. Padala PR, Padala KP, Lensing SY, Jackson AN, Hunter CR, Parkes CM, et al.: Repetitive transcranial magnetic stimulation for apathy in mild cognitive impairment: A double-blind, randomized, sham-controlled, cross-over pilot study. Psychiatry research. 2018; 261:312-8.
34. Roncero C, Kniefel H, Service E, Thiel A, Probst S, Chertkow H: Inferior parietal transcranial direct current stimulation with training improves cognition in anomic Alzheimer's disease and frontotemporal dementia. Alzheimers Dement (N Y). 2017; 3(2):247-53.
35. Drumond Marra HL, Myczkowski ML, Maia Memoria C, Arnaut D, Leite Ribeiro P, Sardinha Mansur CG, et al.: Transcranial Magnetic Stimulation to Address Mild Cognitive Impairment in the Elderly: A Randomized Controlled Study. Behav Neurol. 2015; 2015:287843.
36. Jacobs HI, Riphagen JM, Razat CM, Wiese S, Sack AT: Transcutaneous vagus nerve stimulation boosts associative memory in older individuals. Neurobiol Aging. 2015; 36(5):1860-7.
37. Turriziani P, Smirni D, Zappala G, Mangano GR, Oliveri M, Cipolotti L: Enhancing memory performance with rTMS in healthy subjects and

individuals with Mild Cognitive Impairment: the role of the right dorsolateral prefrontal cortex. Front Hum Neurosci. 2012; 6:62.
38. Floel A, Suttorp W, Kohl O, Kurten J, Lohmann H, Breitenstein C, et al.: Non-invasive brain stimulation improves object-location learning in the elderly. Neurobiol Aging. 2012; 33(8):1682-9.
39. Bagattini C, Zanni M, Barocco F, Caffarra P, Brignani D, Miniussi C, et al.: Enhancing cognitive training effects in Alzheimer's disease: rTMS as an add-on treatment. Brain stimulation. 2020; 13(6):1655-64.
40. Zanardi R, Poletti S, Prestifilippo D, Attanasio F, Barbini B, Colombo C: Transcranial direct current stimulation: A novel approach in the treatment of vascular depression. Brain stimulation. 2020; 13(6):1559-65.
41. Trebbastoni A, Gilio F, D'Antonio F, Cambieri C, Ceccanti M, de Lena C, et al.: Chronic treatment with rivastigmine in patients with Alzheimer's disease: a study on primary motor cortex excitability tested by 5 Hz-repetitive transcranial magnetic stimulation. Clin Neurophysiol. 2012; 123(5):902-9.
42. Beisteiner R, Matt E, Fan C, Baldysiak H, Schonfeld M, Philippi Novak T, et al.: Transcranial Pulse Stimulation with Ultrasound in Alzheimer's Disease-A New Navigated Focal Brain Therapy. Adv Sci (Weinh). 2020; 7(3):1902583.
43. Nguyen JP, Suarez A, Kemoun G, Meignier M, Le Saout E, Damier P, et al.: Repetitive transcranial magnetic stimulation combined with cognitive training for the treatment of Alzheimer's disease. Neurophysiol Clin. 2017; 47(1):47-53.
44. Rabey JM, Dobronevsky E: Repetitive transcranial magnetic stimulation (rTMS) combined with cognitive training is a safe and effective modality for the treatment of Alzheimer's disease: clinical experience. Journal of neural transmission. 2016; 123(12):1449-55.
45. Bentwich J, Dobronevsky E, Aichenbaum S, Shorer R, Peretz R, Khaigrekht M, et al.: Beneficial effect of repetitive transcranial magnetic stimulation combined with cognitive training for the treatment of Alzheimer's disease: a proof of concept study. Journal of neural transmission. 2011; 118(3):463-71.
46. Cotelli M, Manenti R, Cappa SF, Zanetti O, Miniussi C: Transcranial magnetic stimulation improves naming in Alzheimer disease patients at different stages of cognitive decline. Eur J Neurol. 2008; 15(12):1286-92.
47. Liu CS, Herrmann N, Gallagher D, Rajji TK, Kiss A, Vieira D, et al.: A Pilot Study Comparing Effects of Bifrontal Versus Bitemporal Transcranial Direct Current Stimulation in Mild Cognitive Impairment and Mild Alzheimer Disease. The journal of ECT. 2020; 36(3):211-5.

48. Koch G, Bonni S, Pellicciari MC, Casula EP, Mancini M, Esposito R, et al.: Transcranial magnetic stimulation of the precuneus enhances memory and neural activity in prodromal Alzheimer's disease. Neuroimage. 2018; 169:302-11.
49. Anderkova L, Eliasova I, Marecek R, Janousova E, Rektorova I: Distinct Pattern of Gray Matter Atrophy in Mild Alzheimer's Disease Impacts on Cognitive Outcomes of Noninvasive Brain Stimulation. J Alzheimers Dis. 2015; 48(1):251-60.
50. Meinzer M, Lindenberg R, Phan MT, Ulm L, Volk C, Floel A: Transcranial direct current stimulation in mild cognitive impairment: Behavioral effects and neural mechanisms. Alzheimers Dement. 2015; 11(9):1032-40.
51. Eliasova I, Anderkova L, Marecek R, Rektorova I: Non-invasive brain stimulation of the right inferior frontal gyrus may improve attention in early Alzheimer's disease: a pilot study. J Neurol Sci. 2014; 346(1-2):318-22.
52. Boggio PS, Khoury LP, Martins DC, Martins OE, de Macedo EC, Fregni F: Temporal cortex direct current stimulation enhances performance on a visual recognition memory task in Alzheimer disease. Journal of neurology, neurosurgery, and psychiatry. 2009; 80(4):444-7.
53. Hampstead BM: **Enhancing Spatial Navigation Using Non-Invasive Brain Stimulation**. In. ClinicalTrials.gov: NCT01958437; 2018.
54. Rajji TK, Bowie CR, Herrmann N, Pollock BG, Bikson M, Blumberger DM, et al.: Design and Rationale of the PACt-MD Randomized Clinical Trial: Prevention of Alzheimer's dementia with Cognitive remediation plus transcranial direct current stimulation in Mild cognitive impairment and Depression. J Alzheimers Dis. 2020; 76(2):733-51.
55. Xing Y, Wei P, Wang C, Shan Y, Yu Y, Qiao Y, et al.: TRanscranial AlterNating current Stimulation FOR patients with Mild Alzheimer's Disease (TRANSFORM-AD study): Protocol for a randomized controlled clinical trial. Alzheimers Dement (N Y). 2020; 6(1):e12005.
56. Taylor JL, Hambro BC, Strossman ND, Bhatt P, Hernandez B, Ashford JW, et al.: The effects of repetitive transcranial magnetic stimulation in older adults with mild cognitive impairment: a protocol for a randomized, controlled three-arm trial. BMC Neurol. 2019; 19(1):326.
57. Sanches C, Levy R, Benisty S, Volpe-Gillot L, Habert MO, Kas A, et al.: Testing the therapeutic effects of transcranial direct current stimulation (tDCS) in semantic dementia: a double blind, sham controlled, randomized clinical trial. Trials. 2019; 20(1):632.
58. Park J, Oh Y, Chung K, Kim KJ, Kim CO, Park JY: Effect of home-based transcranial direct current stimulation (tDCS) on cognitive function in patients with mild cognitive impairment: a study protocol for a randomized, double-blind, cross-over study. Trials. 2019; 20(1):278.

59. Andrade SM, de Oliveira EA, Alves NT, Dos Santos ACG, de Mendonca C, Sampaio DDA, et al.: Neurostimulation Combined With Cognitive Intervention in Alzheimer's Disease (NeuroAD): Study Protocol of Double-Blind, Randomized, Factorial Clinical Trial. Front Aging Neurosci. 2018; 10:334.
60. Marron EM, Viejo-Sobera R, Quintana M, Redolar-Ripoll D, Rodriguez D, Garolera M: Transcranial magnetic stimulation intervention in Alzheimer's disease: a research proposal for a randomized controlled trial. BMC research notes. 2018; 11(1):648.
61. Woods AJ, Cohen R, Marsiske M, Alexander GE, Czaja SJ, Wu S: Augmenting cognitive training in older adults (The ACT Study): Design and Methods of a Phase III tDCS and cognitive training trial. Contemporary clinical trials. 2018; 65:19-32.
62. Narita Z, Yokoi Y: Transcranial direct current stimulation for depression in Alzheimer's disease: study protocol for a randomized controlled trial. Trials. 2017; 18(1):285.
63. Cheng CP, Chan SS, Mak AD, Chan WC, Cheng ST, Shi L, et al.: Would transcranial direct current stimulation (tDCS) enhance the effects of working memory training in older adults with mild neurocognitive disorder due to Alzheimer's disease: study protocol for a randomized controlled trial. Trials. 2015; 16:479.
64. Thams F, Kuzmina A, Backhaus M, Li SC, Grittner U, Antonenko D, et al.: Cognitive training and brain stimulation in prodromal Alzheimer's disease (AD-Stim)-study protocol for a double-blind randomized controlled phase IIb (monocenter) trial. Alzheimers Res Ther. 2020; 12(1):142.
65. Vrijsen J, Abu-Hanna A, Maeckelberghe EL, De Deyn PP, de Winter AF, Reesink FE, et al.: Uptake and effectiveness of a tailor-made online lifestyle programme targeting modifiable risk factors for dementia among middle-aged descendants of people with recently diagnosed dementia: study protocol of a cluster randomised controlled trial (Demin study). BMJ Open. 2020; 10(10):e039439.
66. Aguera E, Caballero-Villarraso J, Feijoo M, Escribano BM, Conde C, Bahamonde MC, et al.: Clinical and Neurochemical Effects of Transcranial Magnetic Stimulation (TMS) in Multiple Sclerosis: A Study Protocol for a Randomized Clinical Trial. Front Neurol. 2020; 11:750.
67. Holczer A, Nemeth VL, Vekony T, Vecsei L, Klivenyi P, Must A: Non-invasive Brain Stimulation in Alzheimer's Disease and Mild Cognitive Impairment-A State-of-the-Art Review on Methodological Characteristics and Stimulation Parameters. Front Hum Neurosci. 2020;

14:179.
68. Rajji TK: Transcranial Magnetic and Electrical Stimulation in Alzheimer's Disease and Mild Cognitive Impairment: A Review of Randomized Controlled Trials. Clin Pharmacol Ther. 2019; 106(4):776-80.
69. Iimori T, Nakajima S, Miyazaki T, Tarumi R, Ogyu K, Wada M, et al.: Effectiveness of the prefrontal repetitive transcranial magnetic stimulation on cognitive profiles in depression, schizophrenia, and Alzheimer's disease: A systematic review. Progress in neuro-psychopharmacology & biological psychiatry. 2019; 88:31-40.
70. Gonsalvez I, Baror R, Fried P, Santarnecchi E, Pascual-Leone A: Therapeutic Noninvasive Brain Stimulation in Alzheimer's Disease. Curr Alzheimer Res. 2017; 14(4):362-76.
71. Brem AK, Di Iorio R, Fried PJ, Oliveira-Maia AJ, Marra C, Profice P, et al.: Corticomotor Plasticity Predicts Clinical Efficacy of Combined Neuromodulation and Cognitive Training in Alzheimer's Disease. Front Aging Neurosci. 2020; 12:200.
72. Gangemi A, Colombo B, Fabio RA: Effects of short- and long-term neurostimulation (tDCS) on Alzheimer's disease patients: two randomized studies. Aging Clin Exp Res. 2020.
73. Padala PR, Boozer EM, Lensing SY, Parkes CM, Hunter CR, Dennis RA, et al.: Neuromodulation for Apathy in Alzheimer's Disease: A Double-Blind, Randomized, Sham-Controlled Pilot Study. J Alzheimers Dis. 2020.
74. Sabbagh M, Sadowsky C, Tousi B, Agronin ME, Alva G, Armon C, et al.: Effects of a combined transcranial magnetic stimulation (TMS) and cognitive training intervention in patients with Alzheimer's disease. Alzheimers Dement. 2020; 16(4):641-50.
75. Im JJ, Jeong H, Bikson M, Woods AJ, Unal G, Oh JK, et al.: Effects of 6-month at-home transcranial direct current stimulation on cognition and cerebral glucose metabolism in Alzheimer's disease. Brain stimulation. 2019; 12(5):1222-8.
76. Khedr EM, Salama RH, Abdel Hameed M, Abo Elfetoh N, Seif P: Therapeutic Role of Transcranial Direct Current Stimulation in Alzheimer Disease Patients: Double-Blind, Placebo-Controlled Clinical Trial. Neurorehabilitation and neural repair. 2019; 33(5):384-94.
77. Zhang F, Qin Y, Xie L, Zheng C, Huang X, Zhang M: High-frequency repetitive transcranial magnetic stimulation combined with cognitive training improves cognitive function and cortical metabolic ratios in Alzheimer's disease. Journal of neural transmission. 2019; 126(8):1081-94.

78. Alcala-Lozano R, Morelos-Santana E, Cortes-Sotres JF, Garza-Villarreal EA, Sosa-Ortiz AL, Gonzalez-Olvera JJ: Similar clinical improvement and maintenance after rTMS at 5 Hz using a simple vs. complex protocol in Alzheimer's disease. Brain stimulation. 2018; 11(3):625-7.
79. Zhao J, Li Z, Cong Y, Zhang J, Tan M, Zhang H, et al.: Repetitive transcranial magnetic stimulation improves cognitive function of Alzheimer's disease patients. Oncotarget. 2017; 8(20):33864-71.
80. Bystad M, Gronli O, Rasmussen ID, Gundersen N, Nordvang L, Wang-Iversen H, et al.: Transcranial direct current stimulation as a memory enhancer in patients with Alzheimer's disease: a randomized, placebo-controlled trial. Alzheimers Res Ther. 2016; 8(1):13.
81. Lee J, Choi BH, Oh E, Sohn EH, Lee AY: Treatment of Alzheimer's Disease with Repetitive Transcranial Magnetic Stimulation Combined with Cognitive Training: A Prospective, Randomized, Double-Blind, Placebo-Controlled Study. J Clin Neurol. 2016; 12(1):57-64.
82. Rutherford G, Lithgow B, Moussavi Z: Short and Long-term Effects of rTMS Treatment on Alzheimer's Disease at Different Stages: A Pilot Study. J Exp Neurosci. 2015; 9:43-51.
83. Wu Y, Xu W, Liu X, Xu Q, Tang L, Wu S: Adjunctive treatment with high frequency repetitive transcranial magnetic stimulation for the behavioral and psychological symptoms of patients with Alzheimer's disease: a randomized, double-blind, sham-controlled study. Shanghai Arch Psychiatry. 2015; 27(5):280-8.
84. Cotelli M, Manenti R, Brambilla M, Petesi M, Rosini S, Ferrari C, et al.: Anodal tDCS during face-name associations memory training in Alzheimer's patients. Front Aging Neurosci. 2014; 6:38.
85. Khedr EM, Gamal NF, El-Fetoh NA, Khalifa H, Ahmed EM, Ali AM, et al.: A double-blind randomized clinical trial on the efficacy of cortical direct current stimulation for the treatment of Alzheimer's disease. Front Aging Neurosci. 2014; 6:275.
86. Suemoto CK, Apolinario D, Nakamura-Palacios EM, Lopes L, Leite RE, Sales MC, et al.: Effects of a non-focal plasticity protocol on apathy in moderate Alzheimer's disease: a randomized, double-blind, sham-controlled trial. Brain stimulation. 2014; 7(2):308-13.
87. Rabey JM, Dobronevsky E, Aichenbaum S, Gonen O, Marton RG, Khaigrekht M: Repetitive transcranial magnetic stimulation combined with cognitive training is a safe and effective modality for the treatment of Alzheimer's disease: a randomized, double-blind study. Journal of neural transmission. 2013; 120(5):813-9.
88. Ahmed MA, Darwish ES, Khedr EM, El Serogy YM, Ali AM: Effects of low versus high frequencies of repetitive transcranial magnetic

stimulation on cognitive function and cortical excitability in Alzheimer's dementia. J Neurol. 2012; 259(1):83-92.
89. Cotelli M, Calabria M, Manenti R, Rosini S, Zanetti O, Cappa SF, et al.: Improved language performance in Alzheimer disease following brain stimulation. Journal of neurology, neurosurgery, and psychiatry. 2011; 82(7):794-7.
90. Puhan MA, Schunemann HJ, Murad MH, Li T, Brignardello-Petersen R, Singh JA, et al.: A GRADE Working Group approach for rating the quality of treatment effect estimates from network meta-analysis. Bmj. 2014; 349:g5630.
91. Cipriani A, Furukawa TA, Salanti G, Chaimani A, Atkinson LZ, Ogawa Y, et al.: Comparative efficacy and acceptability of 21 antidepressant drugs for the acute treatment of adults with major depressive disorder: a systematic review and network meta-analysis. Lancet. 2018; 391(10128):1357-66.

Sham control	Study name	Std diff in means	Lower limit	Upper limit	*p*-value	Relative weight
rTMS placebo	Ahmed, M.A. (2012)	-0.180	-0.691	0.330	0.488	10.05
rTMS placebo	Brem, A.K. (2020)	0.524	-0.137	1.185	0.120	5.99
rTMS placebo	Cotelli, M. (2011)	0.000	-0.877	0.877	1.000	3.40
rTMS placebo	Lee, J. (2016)	0.484	-0.248	1.217	0.195	4.87
rTMS placebo	Padala, P.R. (2020)	0.031	-0.560	0.622	0.918	7.48
rTMS placebo	Rabey, J.M. (2013)	-0.211	-0.912	0.489	0.555	5.33
rTMS placebo	Rutherford, G. (2015) Stage 1	0.000	-0.980	0.980	1.000	2.72
rTMS placebo	Sabbagh, M. (2020)	0.173	-0.143	0.490	0.282	26.16
rTMS placebo	Wu, Y. (2015)	0.286	-0.107	0.678	0.154	17.01
rTMS placebo	Zhang, F. (2019)	0.274	-0.280	0.828	0.332	8.53
rTMS placebo	Zhao, J. (2017)	0.304	-0.252	0.860	0.283	8.46
**rTMS placebo**	**rTMS placebo overall**	**0.171**	**0.009**	**0.333**	**0.038**	**100.00**
tDCS placebo	Bystad, M. (2016)	0.100	-0.445	0.645	0.719	13.21
tDCS placebo	Cotelli, M. (2014)	0.086	-0.481	0.653	0.766	12.62
tDCS placebo	Gangemi, A. (2020) study 1	-0.604	-1.195	-0.013	0.045	12.00
tDCS placebo	Gangemi, A. (2020) study 2	-0.769	-1.512	-0.025	0.043	8.86
tDCS placebo	Im, J.J. (2019)	-0.330	-1.090	0.431	0.396	8.58
tDCS placebo	Khedr, E.M. (2014)	-0.087	-0.680	0.505	0.772	11.98
tDCS placebo	Khedr, E.M. (2019)	-0.192	-0.624	0.239	0.383	16.78
tDCS placebo	Suemoto, C.K. (2014)	0.399	-0.056	0.855	0.086	15.96
**tDCS placebo**	**tDCS placebo overall**	**-0.124**	**-0.386**	**0.138**	**0.355**	**100.00**

Statistics for each study

Std diff in means and 95% CI

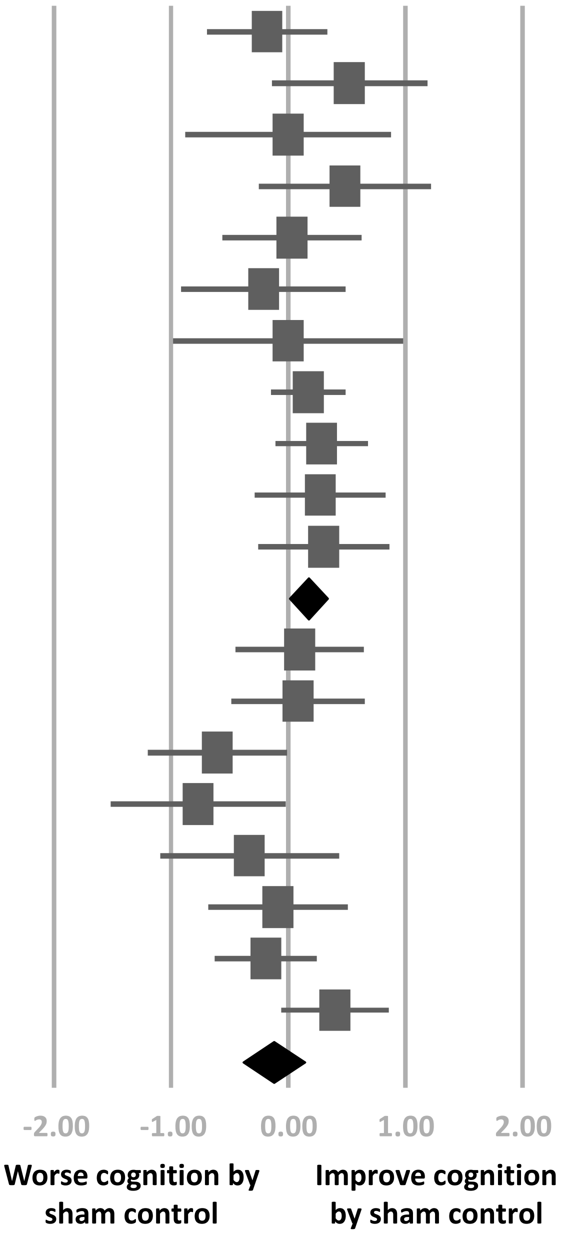

Interaction test *p* value = 0.061

eFigure 1 Test for transitivity assumption of primary outcome: changes of cognition function-overall

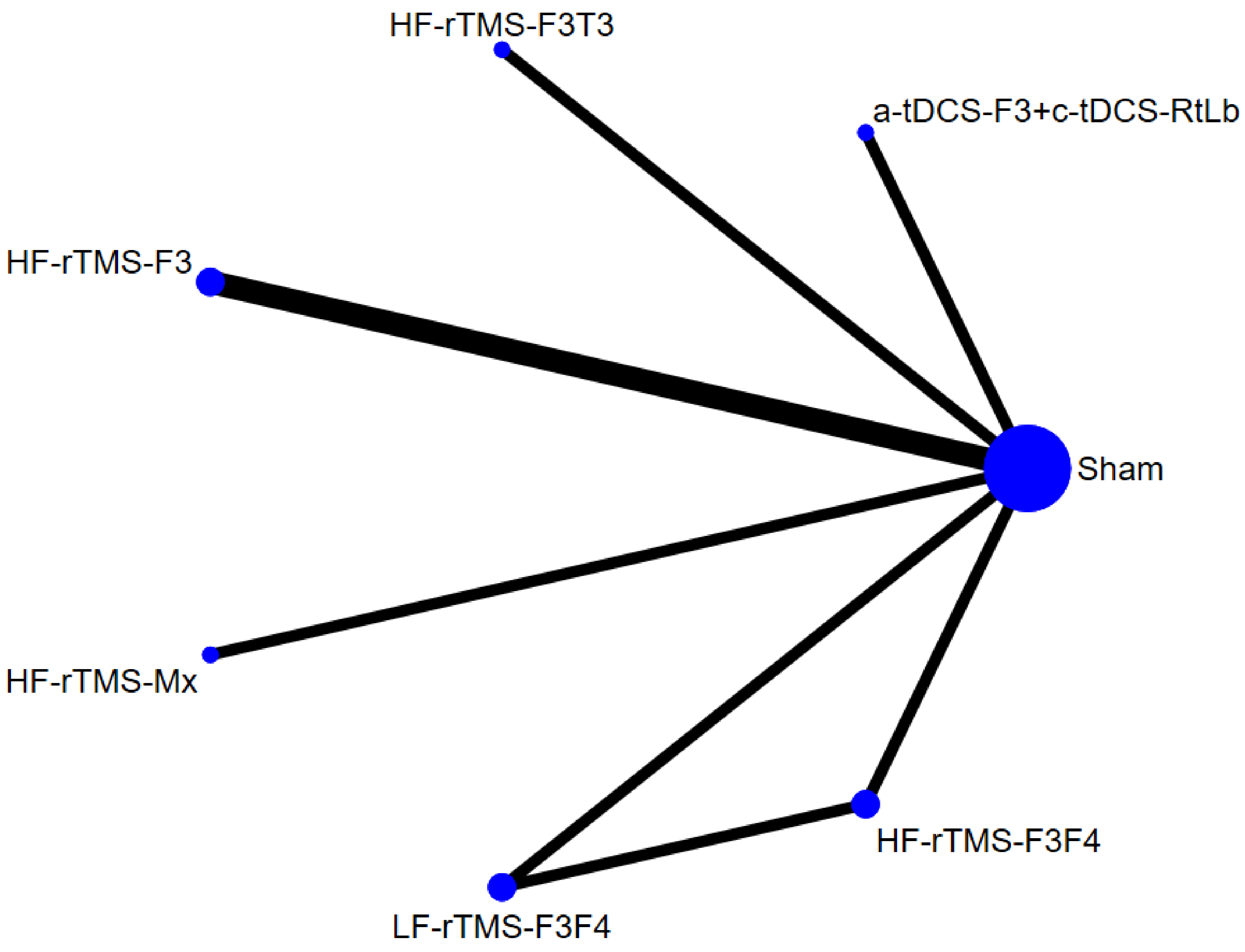

eFigure 2A network structure of NMA of changes of quality of life

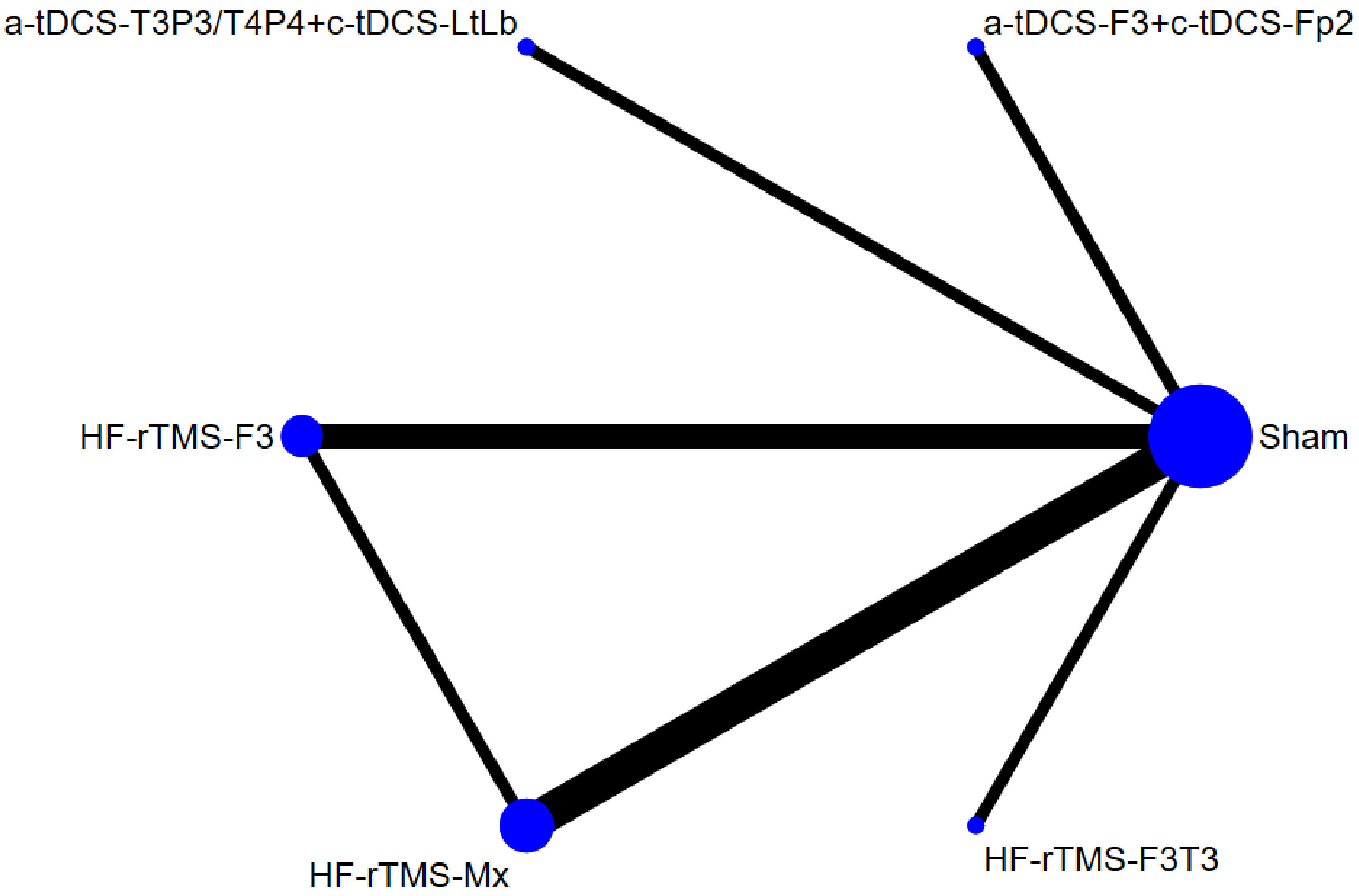

eFigure 2B network structure of NMA of safety profile in aspect of rate of any adverse event

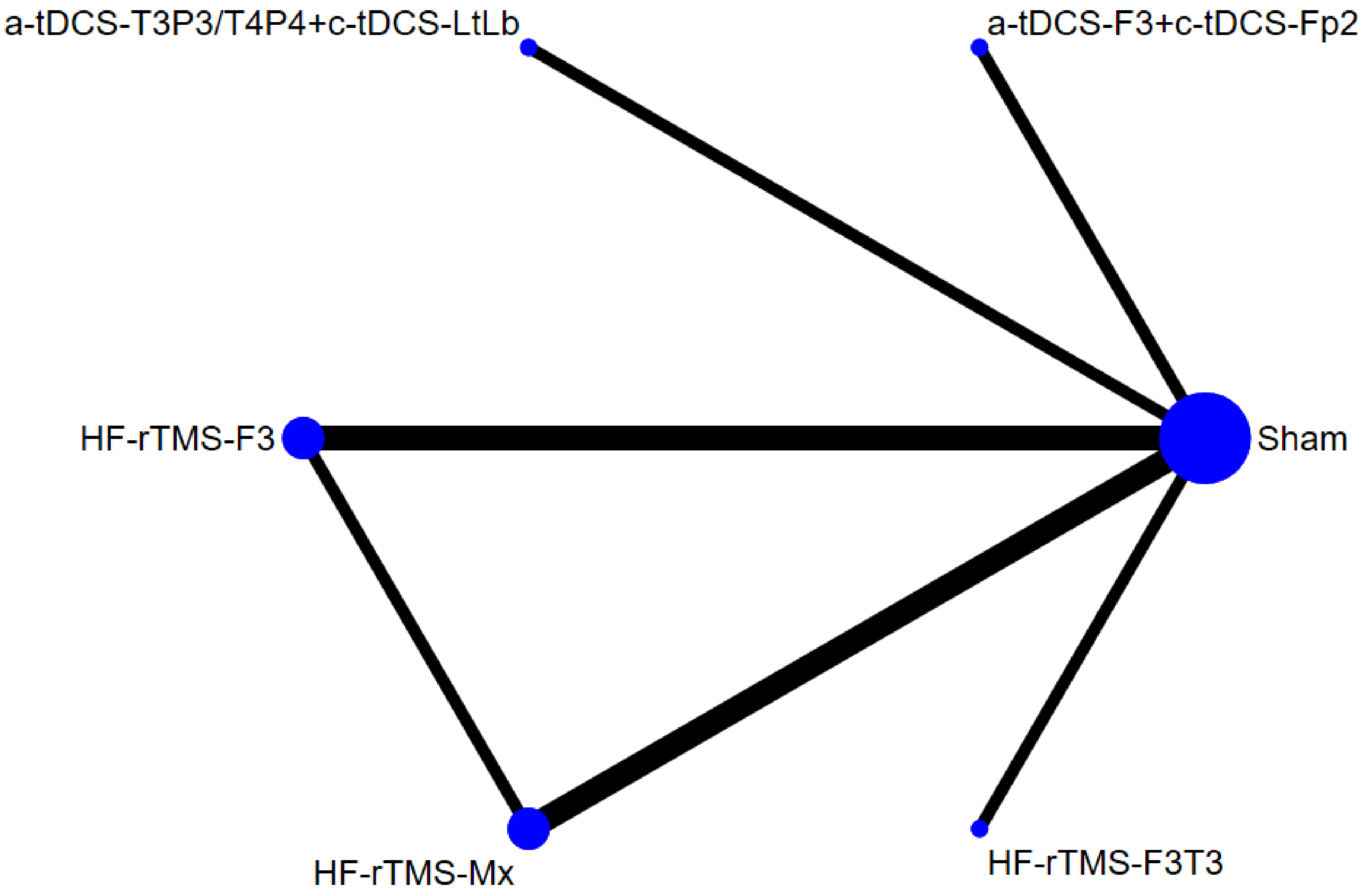

eFigure 2C network structure of NMA of safety profile in aspect of rate of any local discomfort

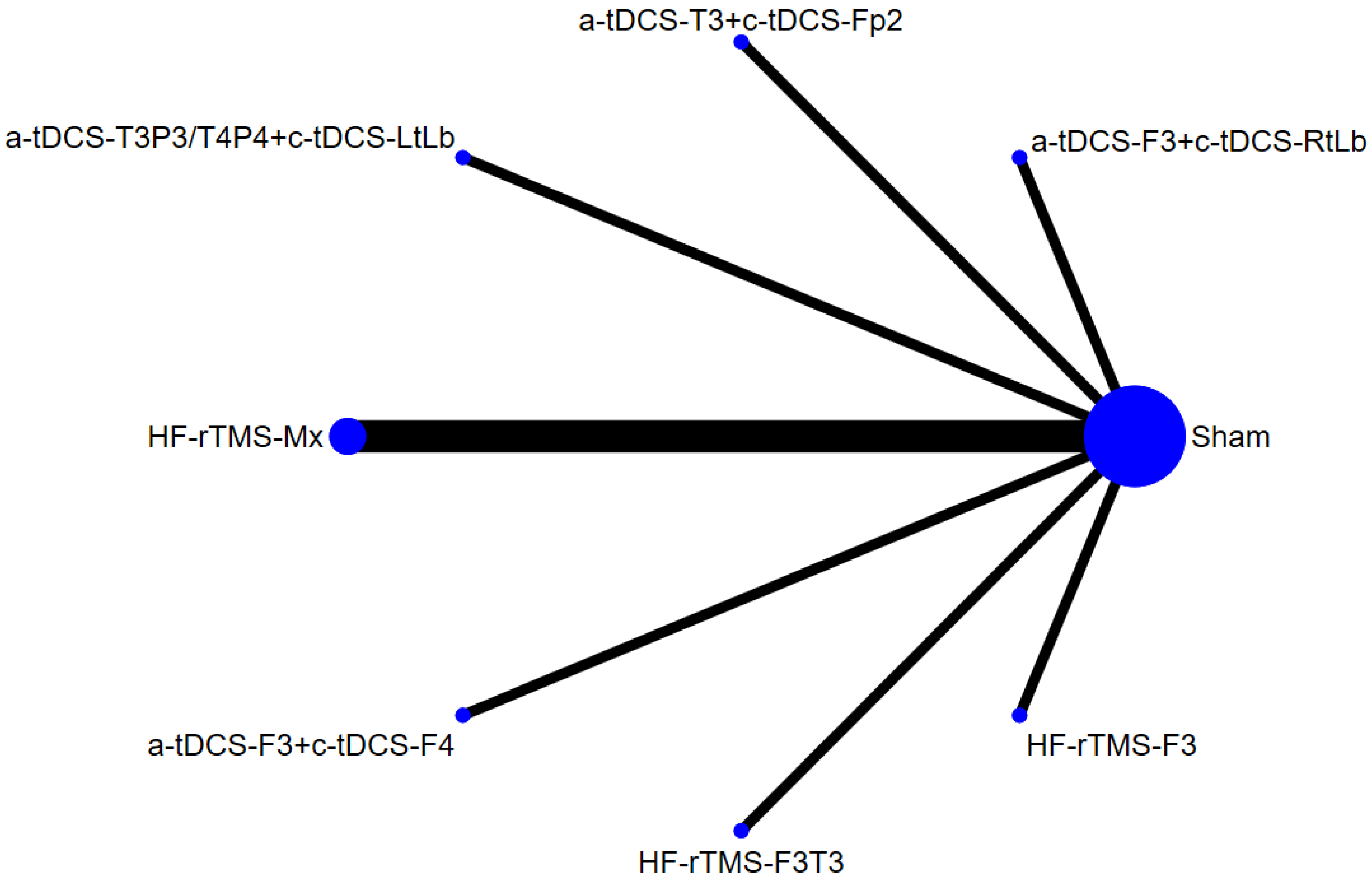

eFigure 2D network structure of NMA of acceptability in aspect of drop-out rate

Figure legend of eFigure 2A-2D

Overall structure of the network meta-analysis. The lines between nodes represent direct comparisons in various trials, and the size of each circle is proportional to the number of participants in each specific treatment. The thickness of the lines is proportional to the number of trials connected to the network.

Abbreviation for eFigure 2A-2D:

AD: Alzheimer's disease; ADAS-Cog: Alzheimer's disease assessment scale-cognitive subscale; a-tDCS-F3 + c-tDCS-F4: anodal tDCS of the left DLPFC and cathodal over the right DLPFC; a-tDCS-F3 + c-tDCS-Fp2: anodal tDCS of the left DLPFC and cathodal over right supraorbital region; a-tDCS-F3 + c-tDCS-RtLb: anodal tDCS of the left DLPFC and cathodal over the right deltoid muscle; a-tDCS-F7 + c-tDCS-Fp2: anodal tDCS of the left frontotemporal lobe and cathodal over right frontal lobe; a-tDCS-T3 + c-tDCS-Fp2: anodal tDCS of the left lateral temporal lobe and cathodal over right frontal lobe; a-tDCS-T3 + c-tDCS-RtLb: anodal tDCS of the left lateral temporal lobe and cathodal over right upper limb; a-tDCS-T3P3/T4P4 + c-tDCS-LtLb: anodal tDCS 2mA alternatively over the bilateral temporo-parietal lobe (T3-P3 or T4-P4) and cathodal over left arm deltoid muscle; CDR: clinical dementia rating; CI: confidence interval; c-tDCS-F3 + a-tDCS-Fp2: cathodal tDCS of the left DLPFC and anodal over right supraorbital region; DLPFC: dorsolateral prefrontal cortex; dTMS: deep TMS; HF-rTMS: high-frequency rTMS; HF-rTMS-F3: high frequency rTMS over left DLPFC; HF-rTMS-F3F4: high frequency rTMS over bilateral DLPFC; HF-rTMS-F3T3: high frequency rTMS over left DLPFC and left lateral temporal lobe; HF-rTMS-F4: high frequency rTMS over right DLPFC; HF-rTMS-Mx: high frequency rTMS multifocal stimulation; IQR: interquartile range; LF-rTMS: low-frequency rTMS; LF-rTMS-F3F4: low frequency rTMS over bilateral DLPFC; MD: mean difference; MMSE: mini-mental state examination; NIBS: noninvasive brain stimulation; NMA: network meta-analysis; OR: odds ratio; PRISMA: preferred reporting items for systematic reviews and the meta-analysis; RCT: randomized controlled trial; rTMS: repetitive transcranial magnetic stimulation; Sham: sham control; SMD: standardized mean difference; SUCRA: surface under the cumulative ranking curve; TBS: theta-burst stimulation; tDCS: transcranial direct current stimulation

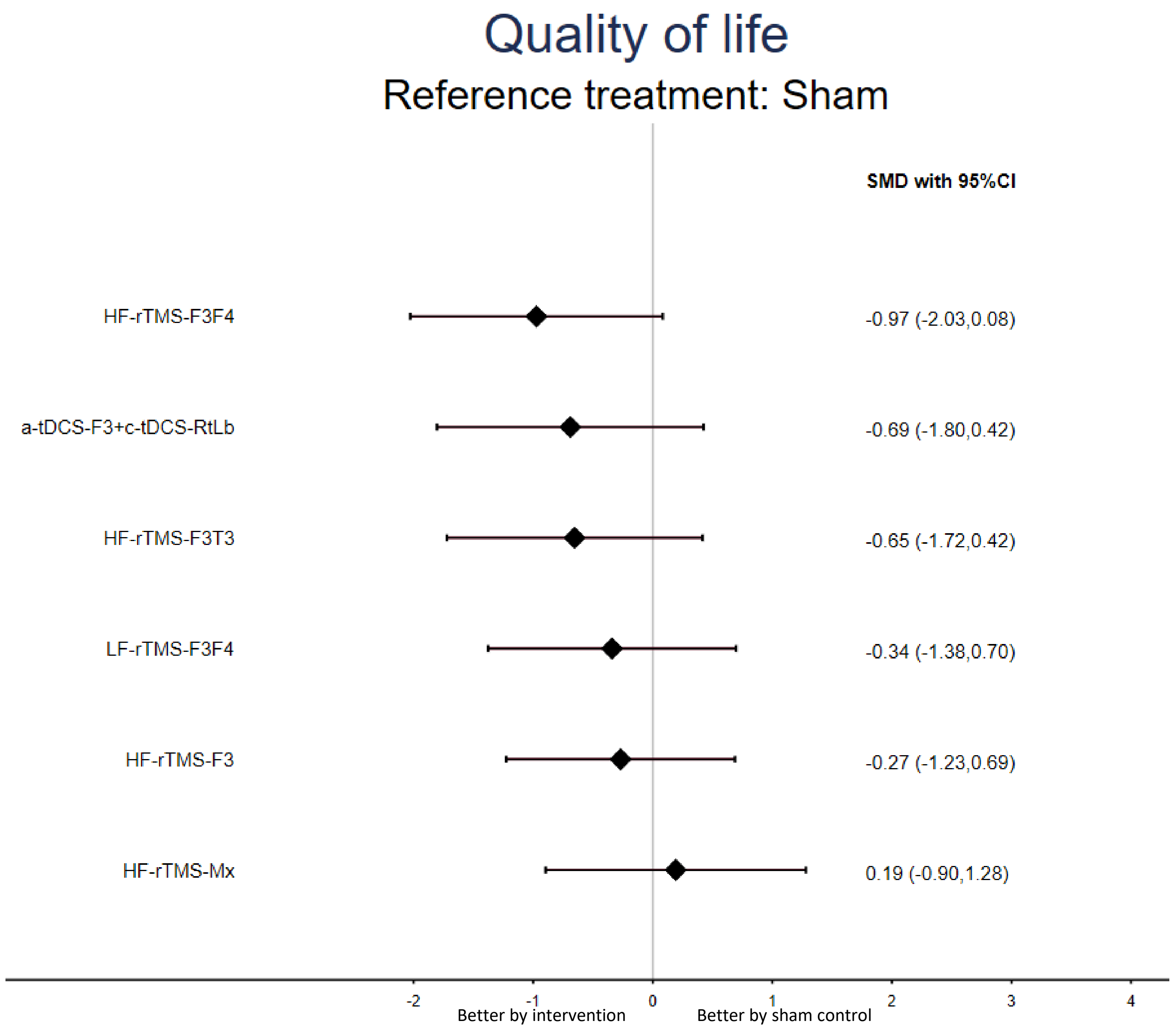

eFigure 3A forest plot of NMA of change of quality of life

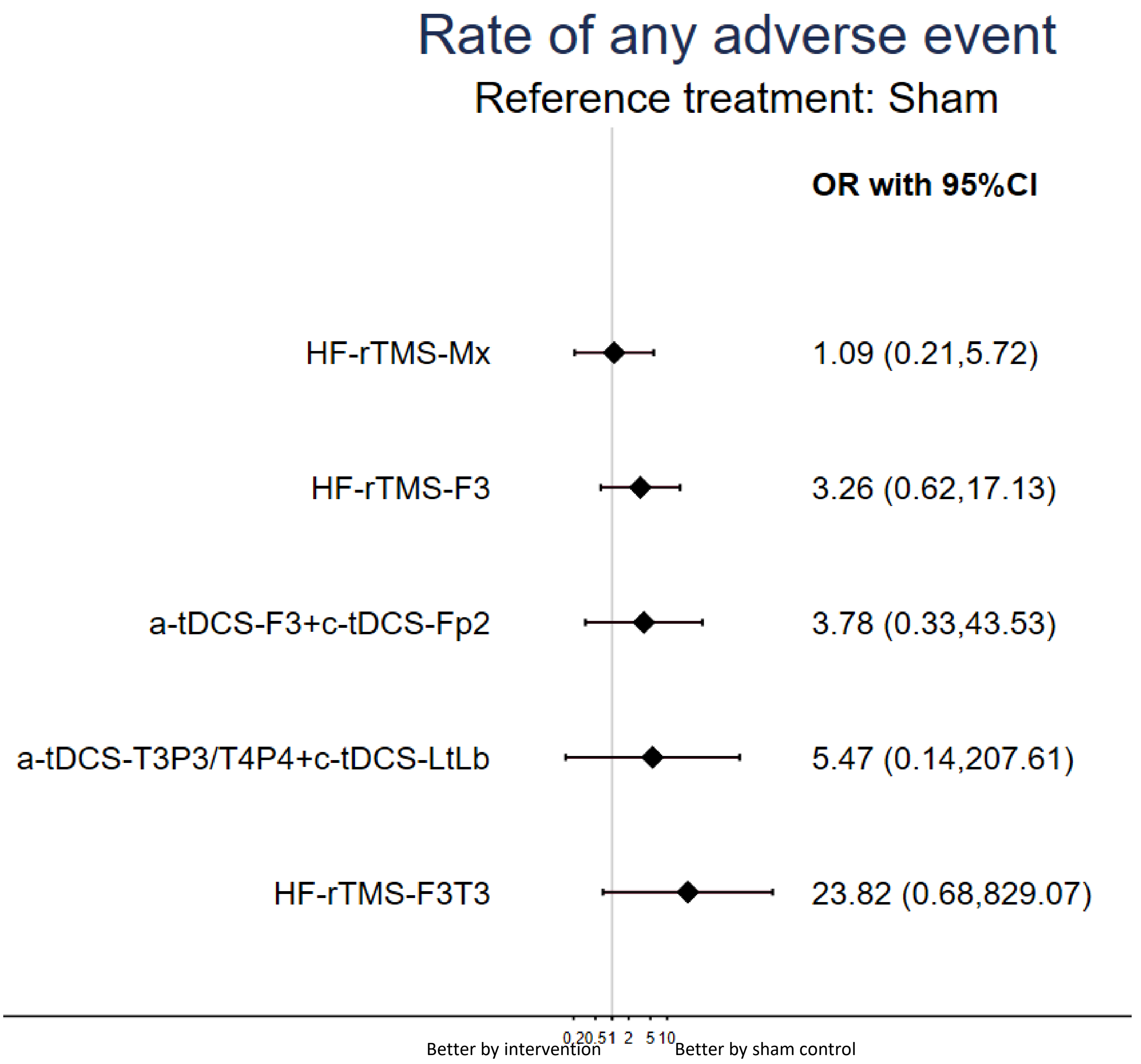

eFigure 3B forest plot of NMA of safety profile in aspect of rate of any adverse event

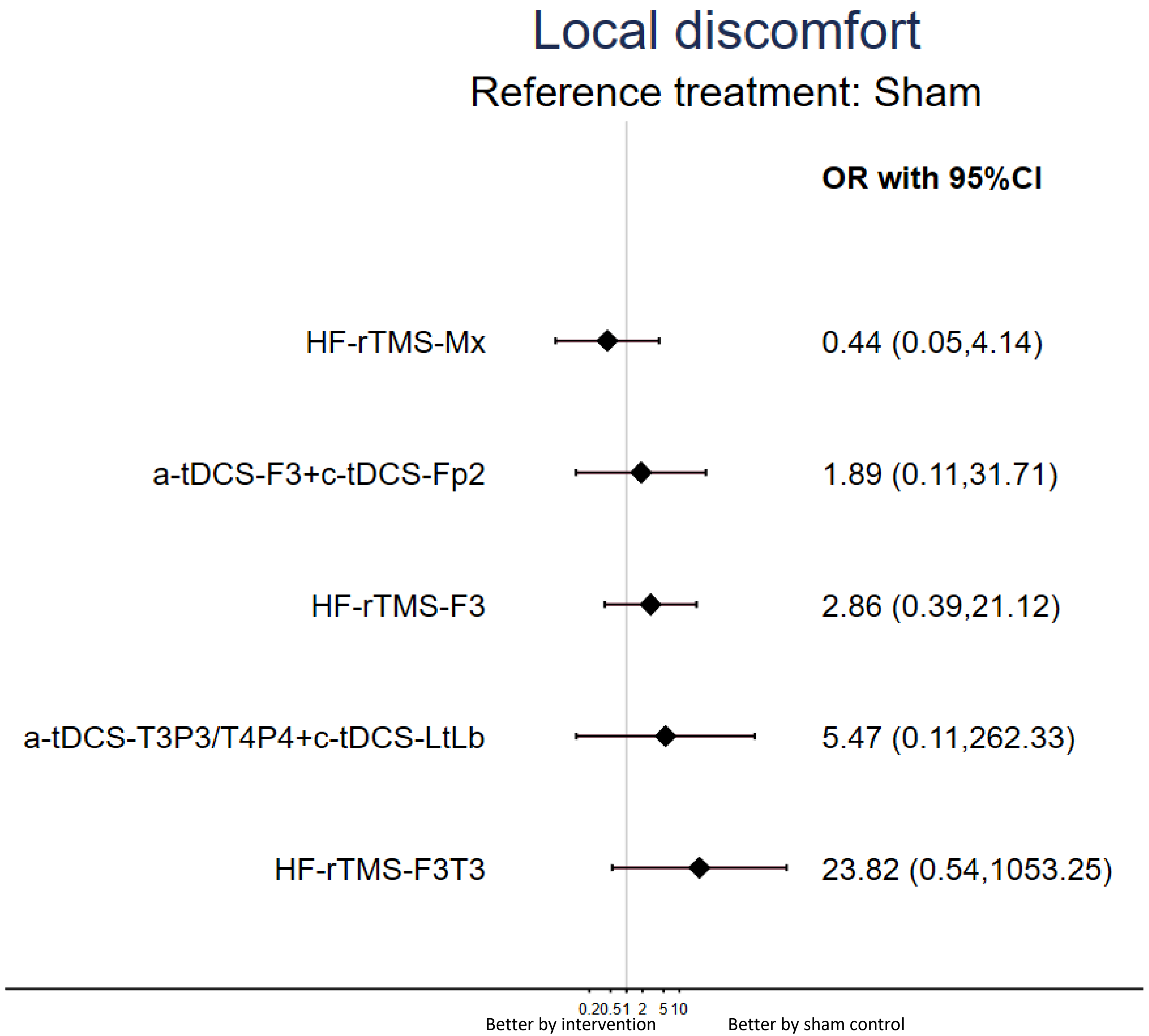

eFigure 3C forest plot of NMA of safety profile in aspect of rate of any local discomfort

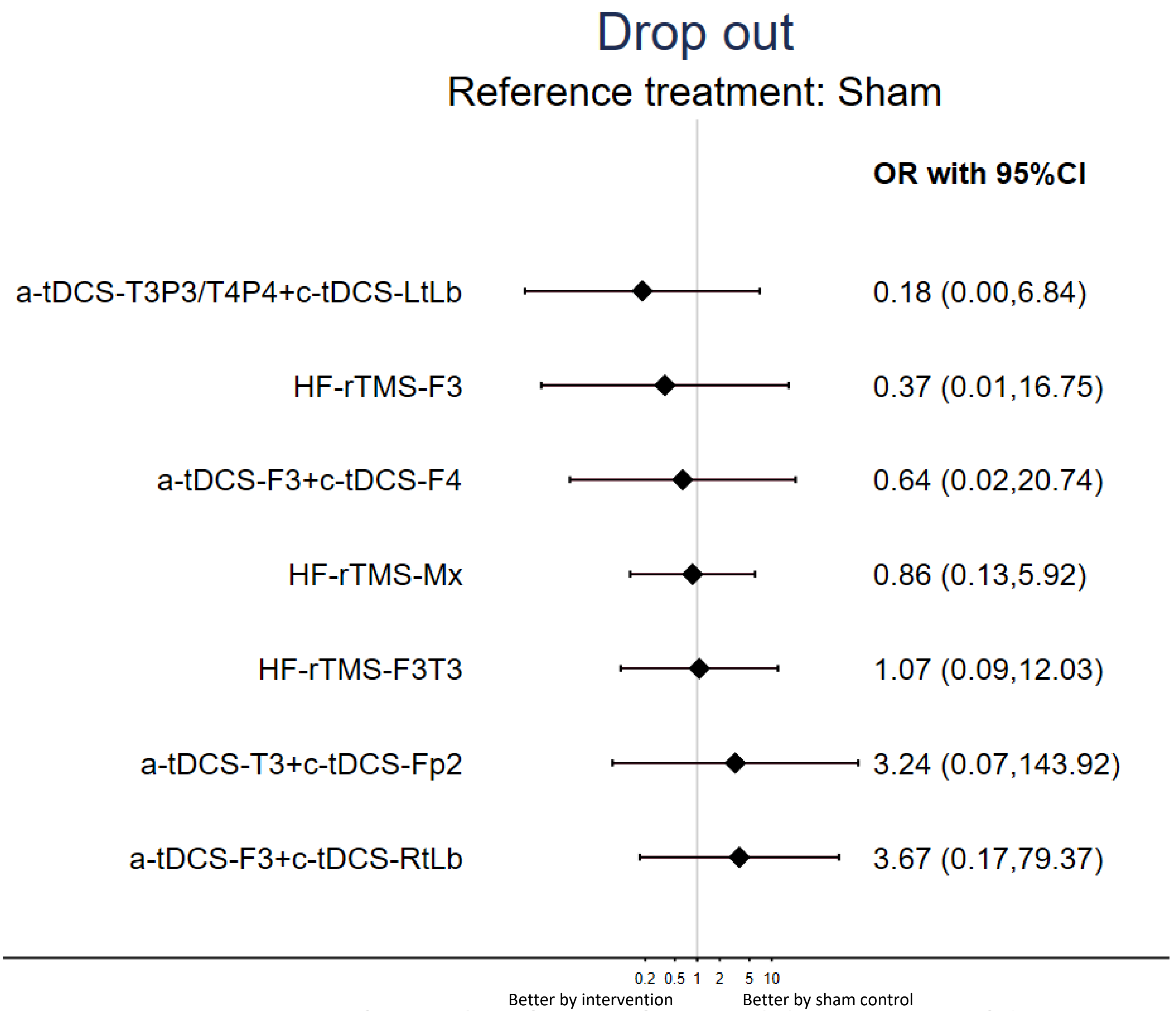

eFigure 3D forest plot of NMA of acceptability in aspect of drop-out rate

Abbreviation for eFigure 3A-3D:

AD: Alzheimer’s disease; ADAS-Cog: Alzheimer's disease assessment scale-cognitive subscale; a-tDCS-F3 + c-tDCS-F4: anodal tDCS of the left DLPFC and cathodal over the right DLPFC; a-tDCS-F3 + c-tDCS-Fp2: anodal tDCS of the left DLPFC and cathodal over right supraorbital region; a-tDCS-F3 + c-tDCS-RtLb: anodal tDCS of the left DLPFC and cathodal over the right deltoid muscle; a-tDCS-F7 + c-tDCS-Fp2: anodal tDCS of the left frontotemporal lobe and cathodal over right frontal lobe; a-tDCS-T3 + c-tDCS-Fp2: anodal tDCS of the left lateral temporal lobe and cathodal over right frontal lobe; a-tDCS-T3 + c-tDCS-RtLb: anodal tDCS of the left lateral temporal lobe and cathodal over right upper limb; a-tDCS-T3P3/T4P4 + c-tDCS-LtLb: anodal tDCS 2mA alternatively over the bilateral temporo-parietal lobe (T3-P3 or T4-P4) and cathodal over left arm deltoid muscle; CDR: clinical dementia rating; CI: confidence interval; c-tDCS-F3 + a-tDCS-Fp2: cathodal tDCS of the left DLPFC and anodal over right supraorbital region; DLPFC: dorsolateral prefrontal cortex; dTMS: deep TMS; HF-rTMS: high-frequency rTMS; HF-rTMS-F3: high frequency rTMS over left DLPFC; HF-rTMS-F3F4: high frequency rTMS over bilateral DLPFC; HF-rTMS-F3T3: high frequency rTMS over left DLPFC and left lateral temporal lobe; HF-rTMS-F4: high frequency rTMS over right DLPFC; HF-rTMS-Mx: high frequency rTMS multifocal stimulation; IQR: interquartile range; LF-rTMS: low-frequency rTMS; LF-rTMS-F3F4: low frequency rTMS over bilateral DLPFC; MD: mean difference; MMSE: mini-mental state examination; NIBS: noninvasive brain stimulation; NMA: network meta-analysis; OR: odds ratio; PRISMA: preferred reporting items for systematic reviews and the meta-analysis; RCT: randomized controlled trial; rTMS: repetitive transcranial magnetic stimulation; Sham: sham control; SMD: standardized mean difference; SUCRA: surface under the cumulative ranking curve; TBS: theta-burst stimulation; tDCS: transcranial direct current stimulation

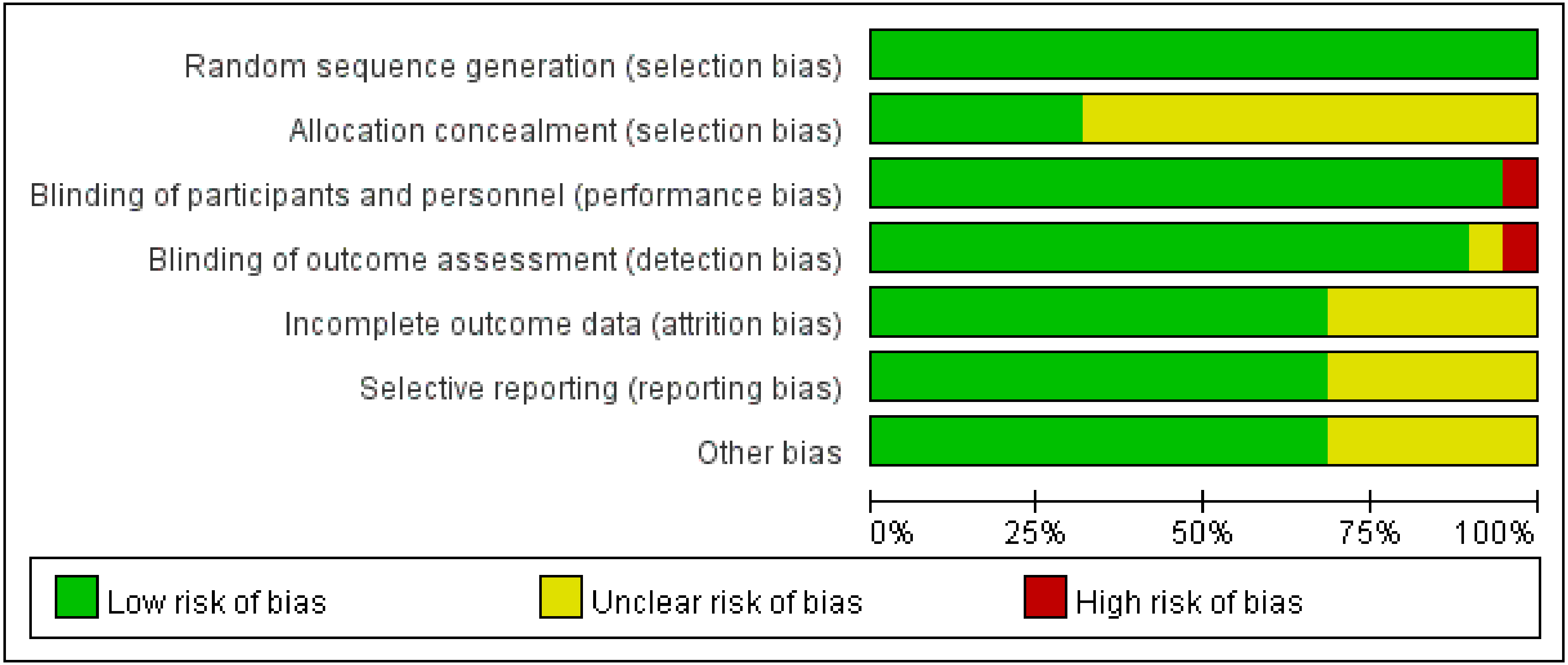

eFigure 4A overview of risk of bias

	Random sequence generation (selection bias)	Allocation concealment (selection bias)	Blinding of participants and personnel (performance bias)	Blinding of outcome assessment (detection bias)	Incomplete outcome data (attrition bias)	Selective reporting (reporting bias)	Other bias
Ahmed, M.A. (2012)	+	+	+	+	+	+	?
Alcala-Lozano, R. (2018)	+	?	−	−	+	+	+
Brem, A.K. (2020)	+	+	+	+	+	+	?
Bystad, M. (2016)	+	+	+	?	+	?	+
Cotelli, M. (2011)	+	?	+	+	+	+	?
Cotelli, M. (2014)	+	?	+	+	+	?	+
Gangemi, A. (2020)	+	?	+	+	+	?	+
Im, J.J. (2019)	+	?	+	+	+	?	+
Khedr, E.M. (2014)	+	+	+	+	+	?	+
Khedr, E.M. (2019)	+	+	+	+	+	+	?
Lee, J. (2016)	+	?	+	+	?	+	+
Padala, P.R. (2020)	+	?	+	+	+	+	?
Rabey, J.M. (2013)	+	?	+	+	+	?	+
Rutherford, G. (2015) Stage 1	+	?	+	+	+	+	?
Sabbagh, M. (2020)	+	?	+	+	?	+	+
Suemoto, C.K. (2014)	+	+	+	+	?	+	+
Wu, Y. (2015)	+	?	+	+	?	+	+
Zhang, F. (2019)	+	?	+	+	?	+	+
Zhao, J. (2017)	+	?	+	+	?	+	+

eFigure 4B detailed risk of bias in each study

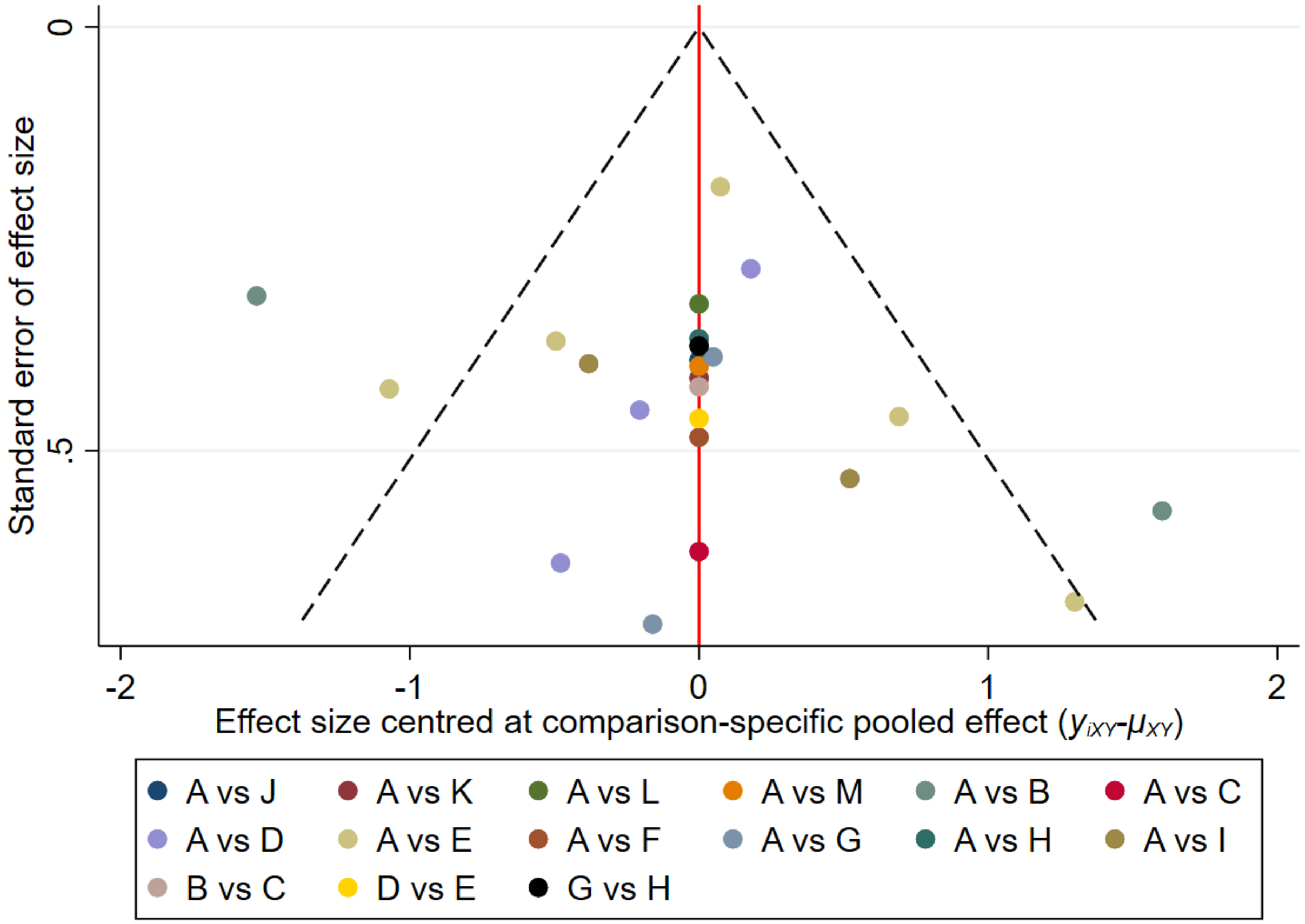

eFigure 5A Funnel plot of changes of cognition function: overall

Treatments used in eFigure 4A

A:	Sham
B:	a-tDCS-F3 + c-tDCS-Fp2
C:	c-tDCS-F3 + a-tDCS-Fp2
D:	HF-rTMS-F3
E:	HF-rTMS-Mx
F:	a-tDCS-F3 + c-tDCS-F4
G:	HF-rTMS-F3F4
H:	LF-rTMS-F3F4
I:	a-tDCS-F7 + c-tDCS-Fp2
J:	HF-rTMS-F3T3
K:	a-tDCS-F3 + c-tDCS-RtLb
L:	a-tDCS-T3P3/T4P4 + c-tDCS-LtLb
M:	a-tDCS-T3 + c-tDCS-Fp2

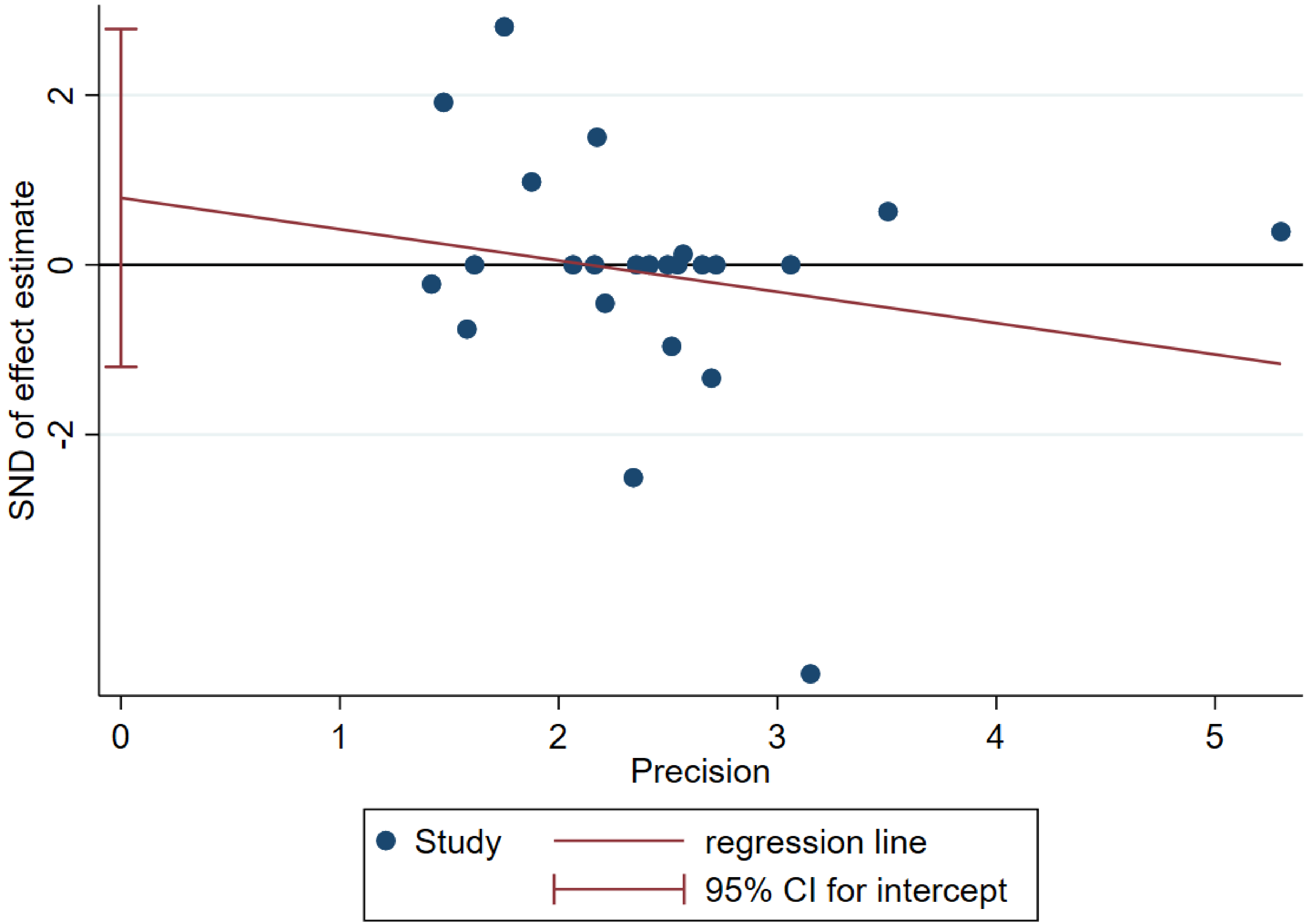

eFigure 5B Egger's regression of changes of cognition function: overall

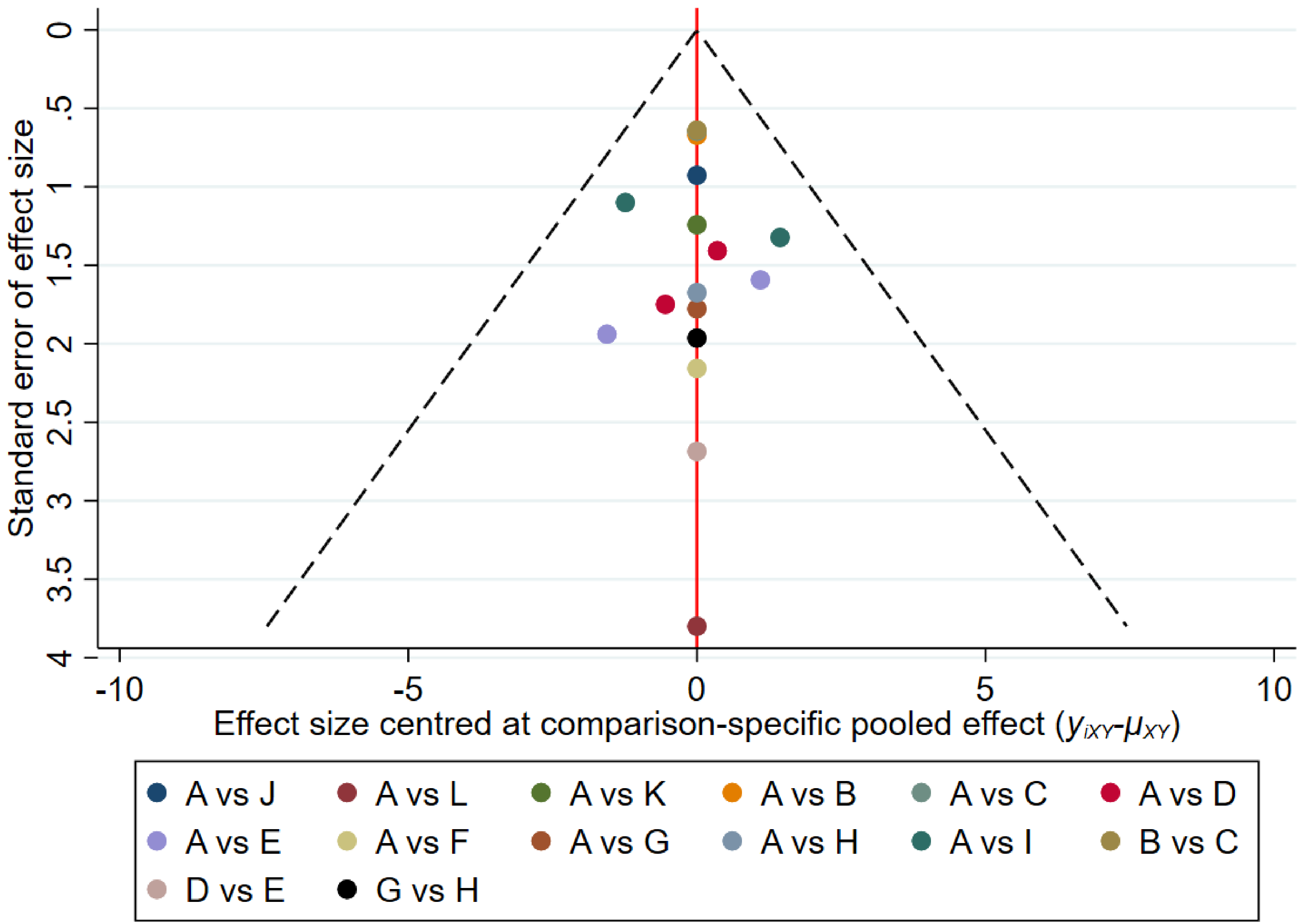

eFigure 5C Funnel plot of changes of cognition function: MMSE measurement

Treatments used in eFigure 4C

A: Sham

B: a-tDCS-F3 + c-tDCS-Fp2

C: c-tDCS-F3 + a-tDCS-Fp2

D: HF-rTMS-F3

E: HF-rTMS-Mx

F: a-tDCS-F3 + c-tDCS-F4

G: HF-rTMS-F3F4

H: LF-rTMS-F3F4

I: a-tDCS-F7 + c-tDCS-Fp2

J: HF-rTMS-F3T3

K: a-tDCS-F3 + c-tDCS-RtLb

L: a-tDCS-T3 + c-tDCS-Fp2

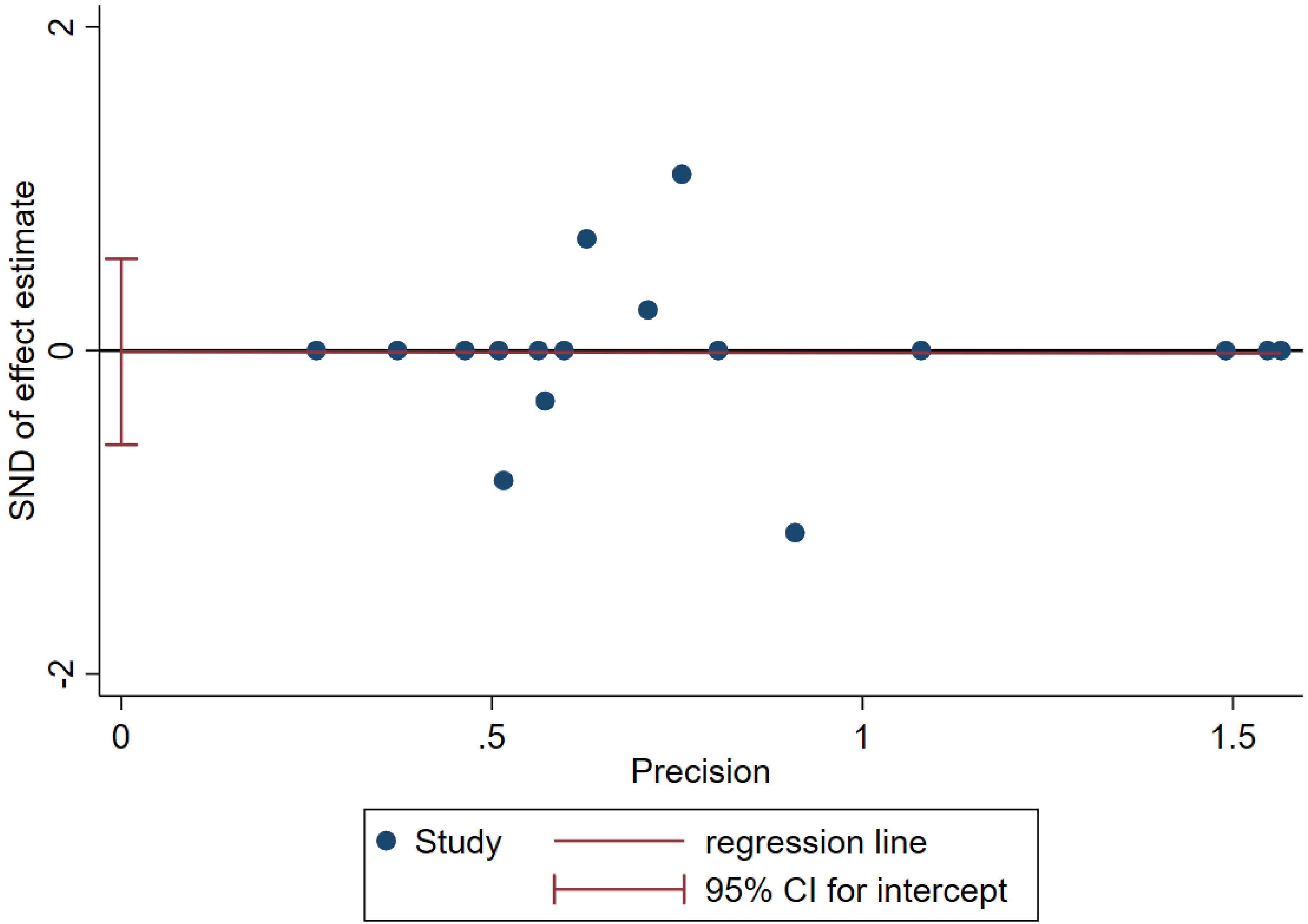

eFigure 5D Egger's regression of changes of cognition function: MMSE measurement

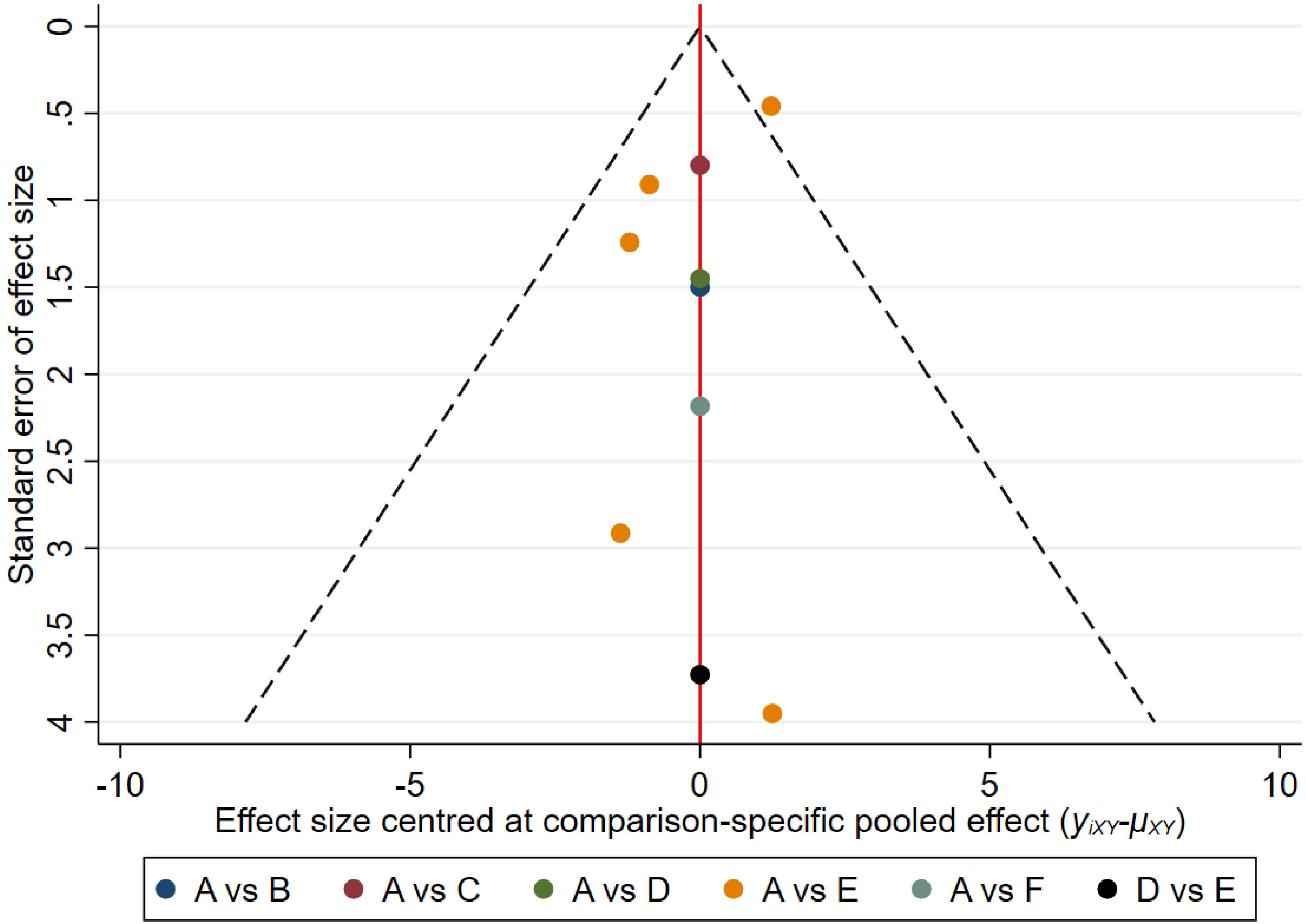

eFigure 5E Funnel plot of changes of cognition function: ADAS-Cog measurement

Treatments used in eFigure 4E

A:	Sham
B:	a-tDCS-F3 + c-tDCS-Fp2
C:	HF-rTMS-F3T3
D:	HF-rTMS-F3
E:	HF-rTMS-Mx
F:	HF-rTMS-F3F4

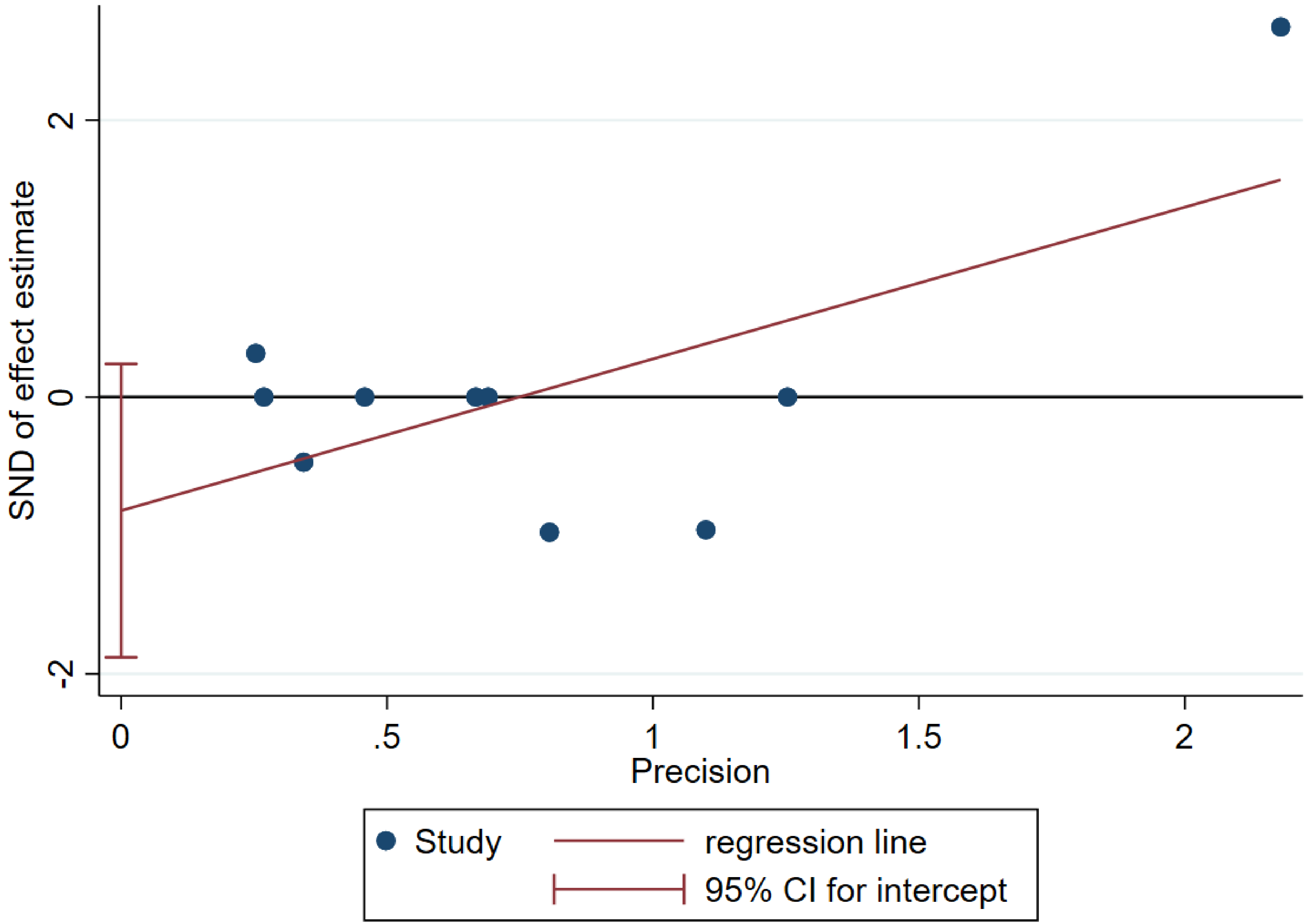

eFigure 5F Egger's regression of changes of cognition function: ADAS-Cog measurement

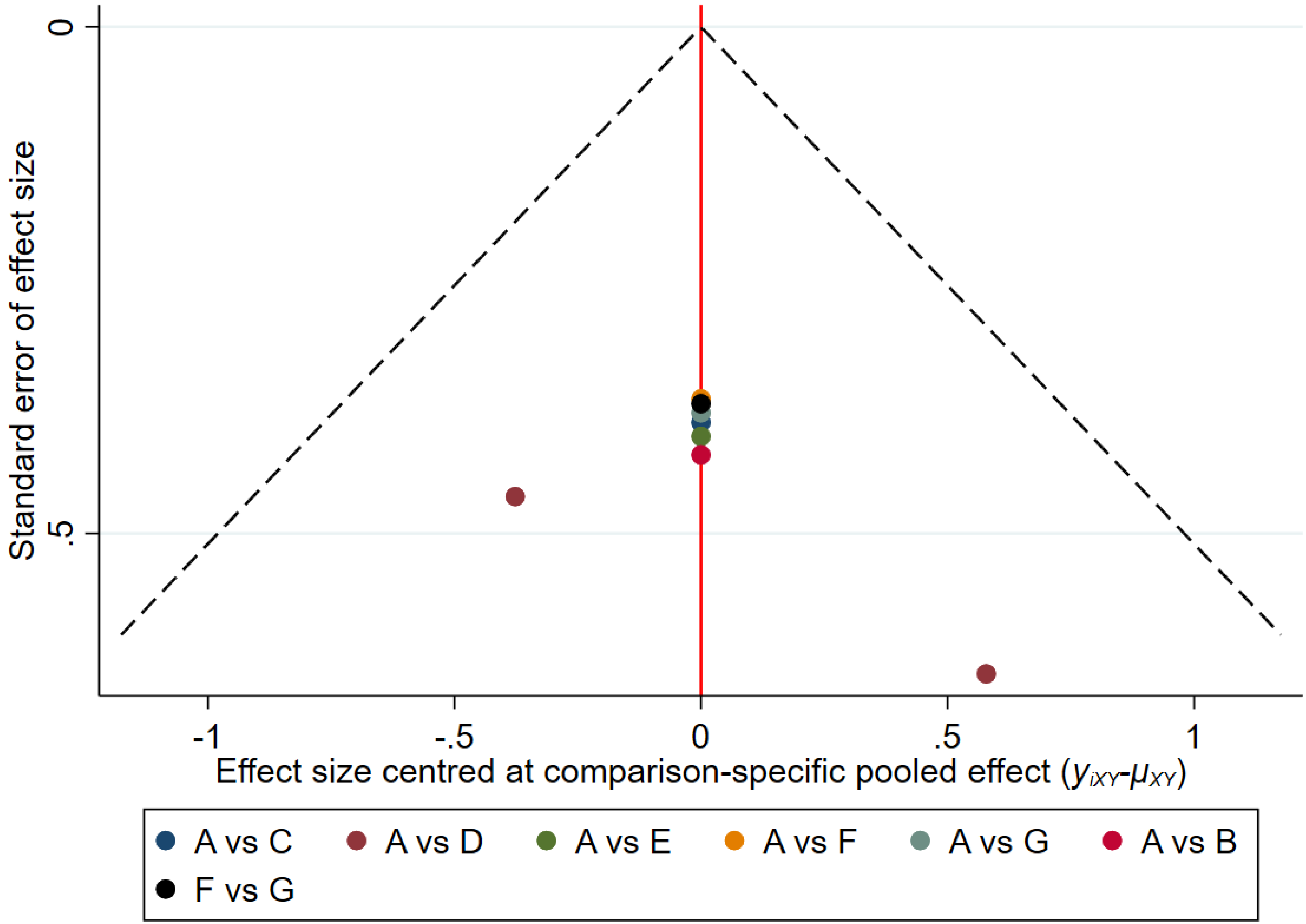

eFigure 5G Funnel plot of changes of quality of life

Treatments used in eFigure 4G

A: Sham

B: a-tDCS-F3 + c-tDCS-RtLb

C: HF-rTMS-F3T3

D: HF-rTMS-F3

E: HF-rTMS-Mx

F: LF-rTMS-F3F4

G: HF-rTMS-F3F4

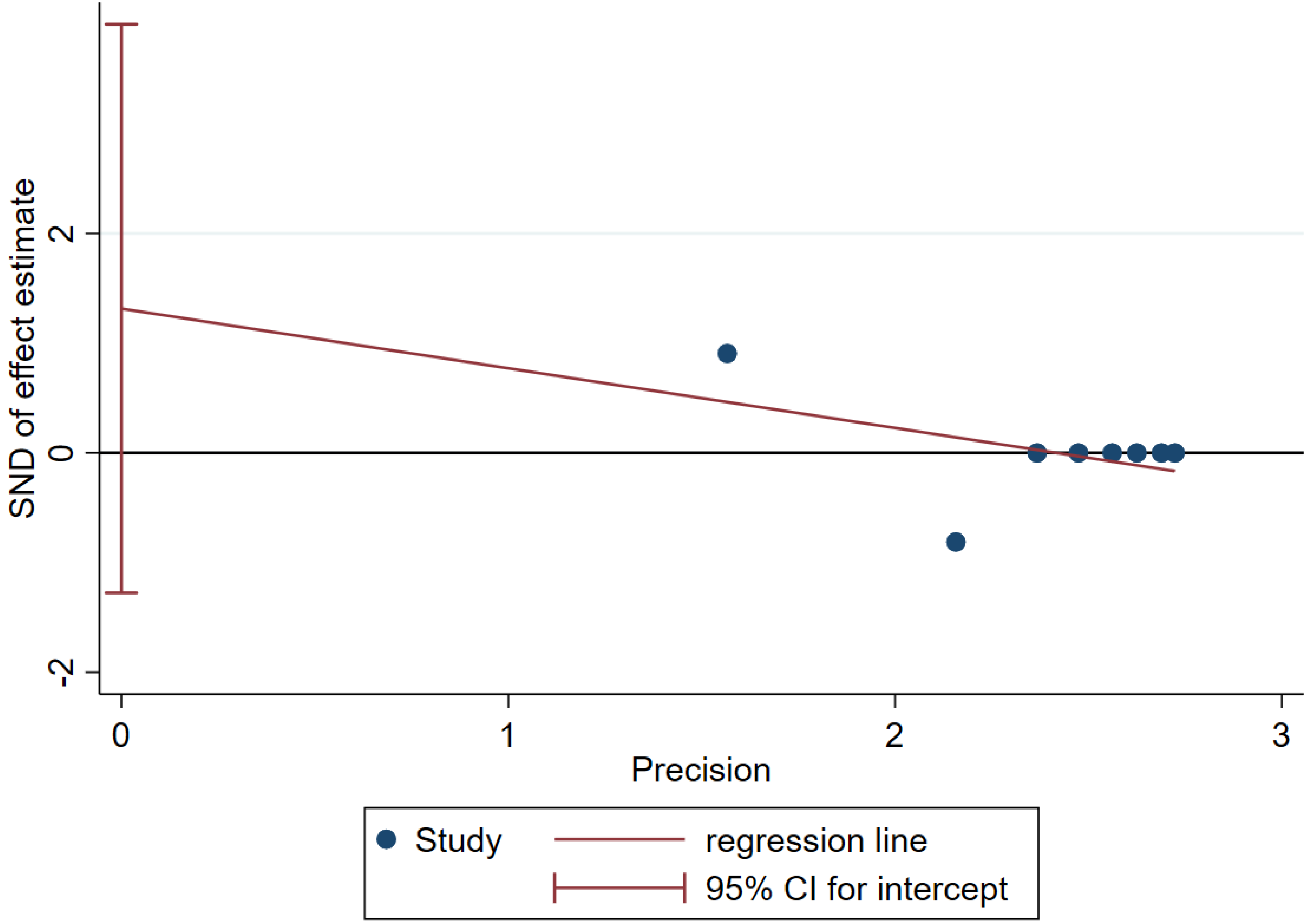

eFigure 5H Egger's regression of changes of quality of life

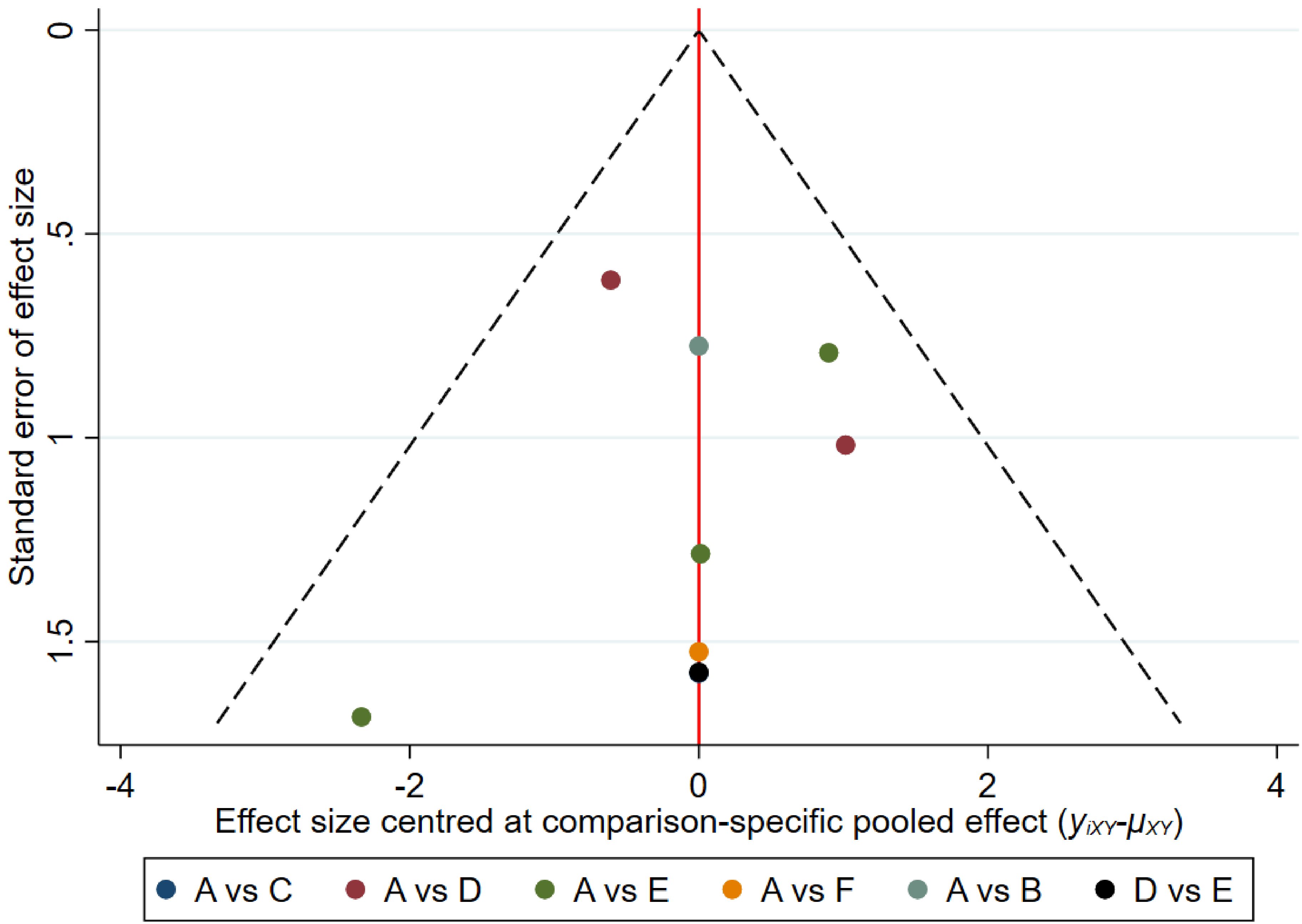

eFigure 5I Funnel plot of safety profile in aspect of rate of any adverse event

Treatments used in eFigure 4I

A:	Sham
B:	a-tDCS-F3 + c-tDCS-Fp2
C:	a-tDCS-T3P3/T4P4 + c-tDCS-LtLb
D:	HF-rTMS-F3
E:	HF-rTMS-Mx
F:	HF-rTMS-F3T3

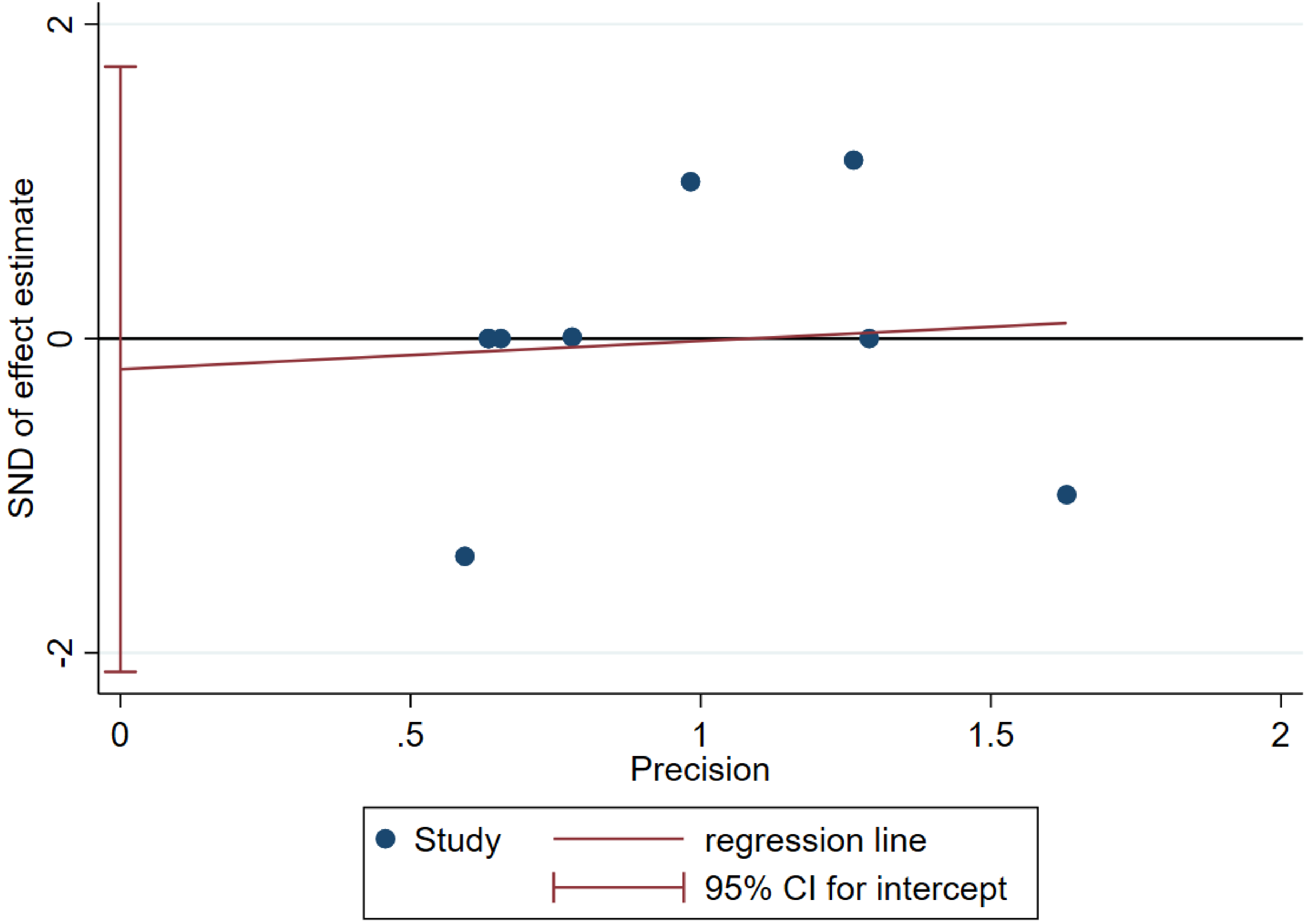

eFigure 5J Egger's regression of safety profile in aspect of rate of any adverse event

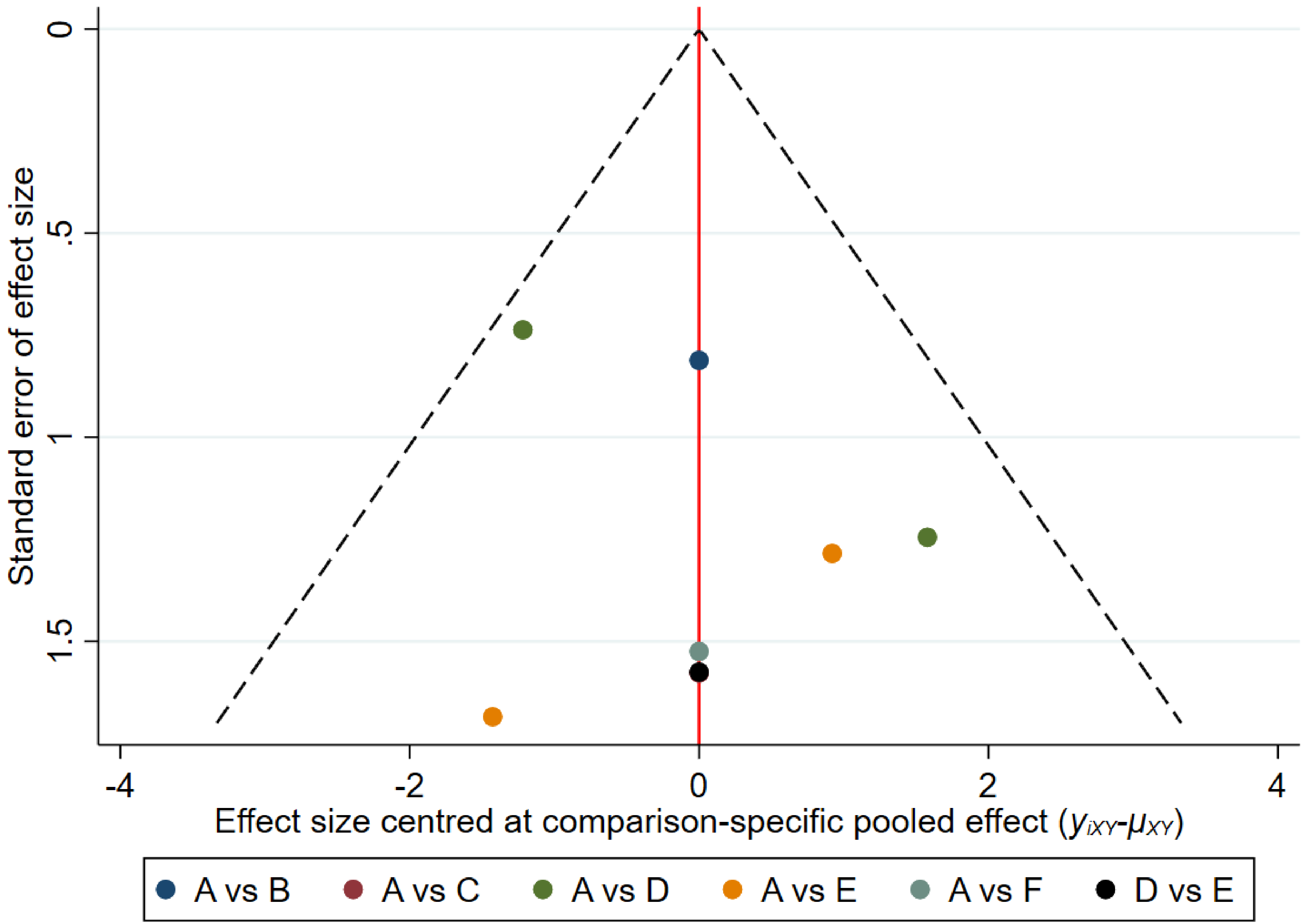

eFigure 5K Funnel plot of safety profile in aspect of rate of any local discomfort

Treatments used in eFigure 4K

A: Sham

B: a-tDCS-F3 + c-tDCS-Fp2

C: a-tDCS-T3P3/T4P4 + c-tDCS-LtLb

D: HF-rTMS-F3

E: HF-rTMS-Mx

F: HF-rTMS-F3T3

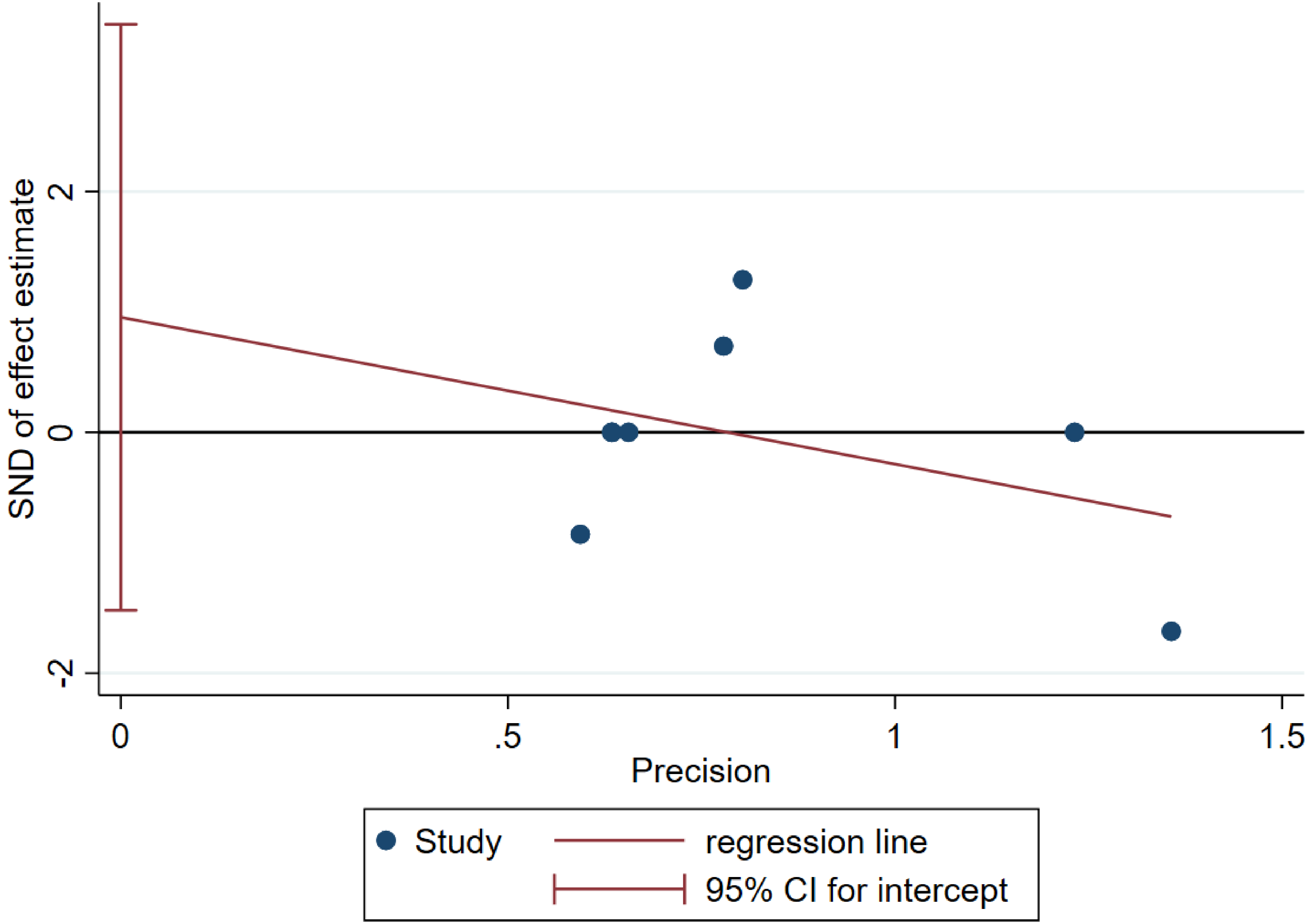

eFigure 5L Egger's regression of safety profile in aspect of rate of any local discomfort

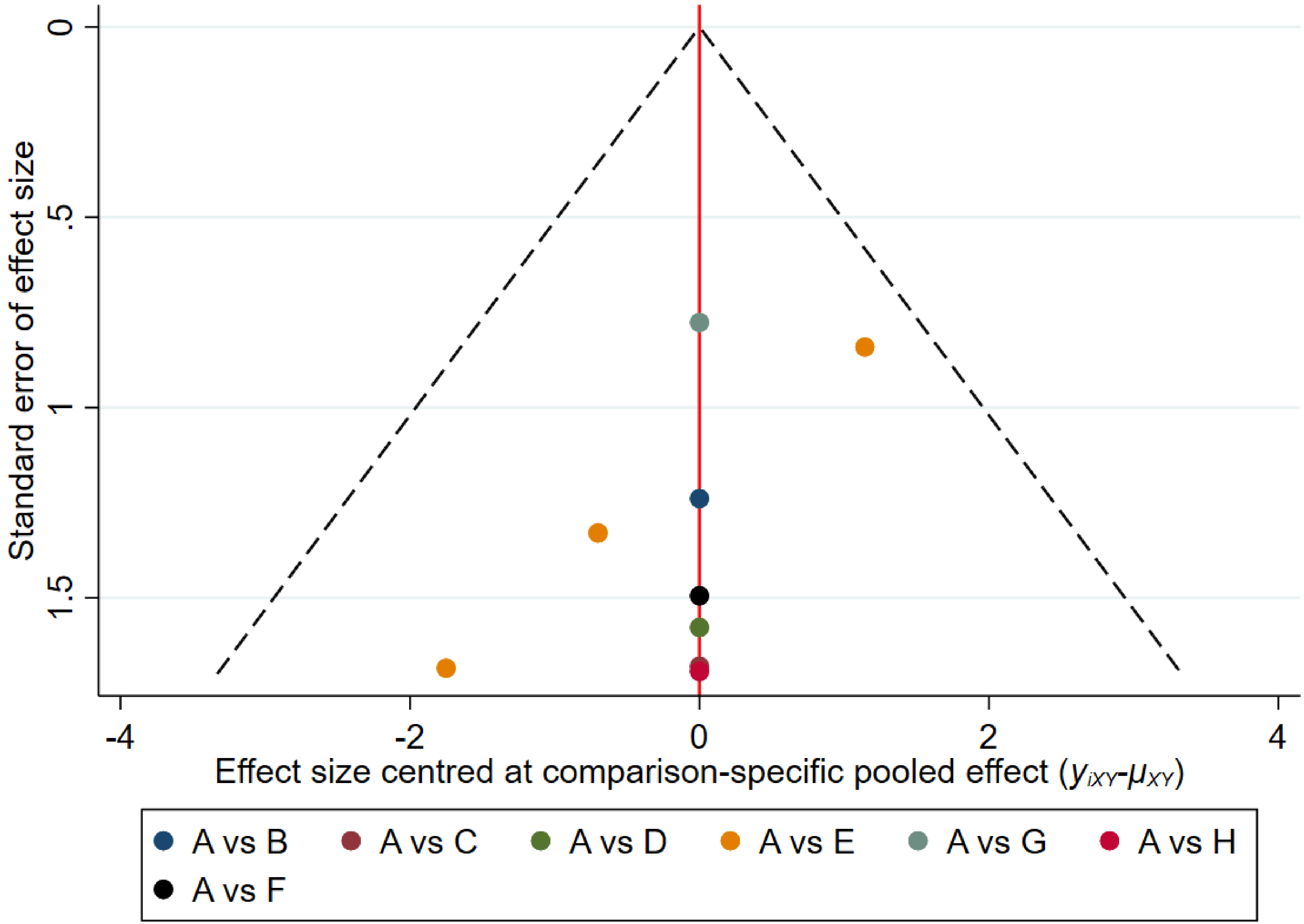

eFigure 5M Funnel plot of acceptability in aspect of drop-out rate

Treatments used in eFigure 4M

A:	Sham
B:	a-tDCS-F3 + c-tDCS-RtLb
C:	a-tDCS-T3 + c-tDCS-Fp2
D:	a-tDCS-T3P3/T4P4 + c-tDCS-LtLb
E:	HF-rTMS-Mx
F:	a-tDCS-F3 + c-tDCS-F4
G:	HF-rTMS-F3T3
H:	HF-rTMS-F3

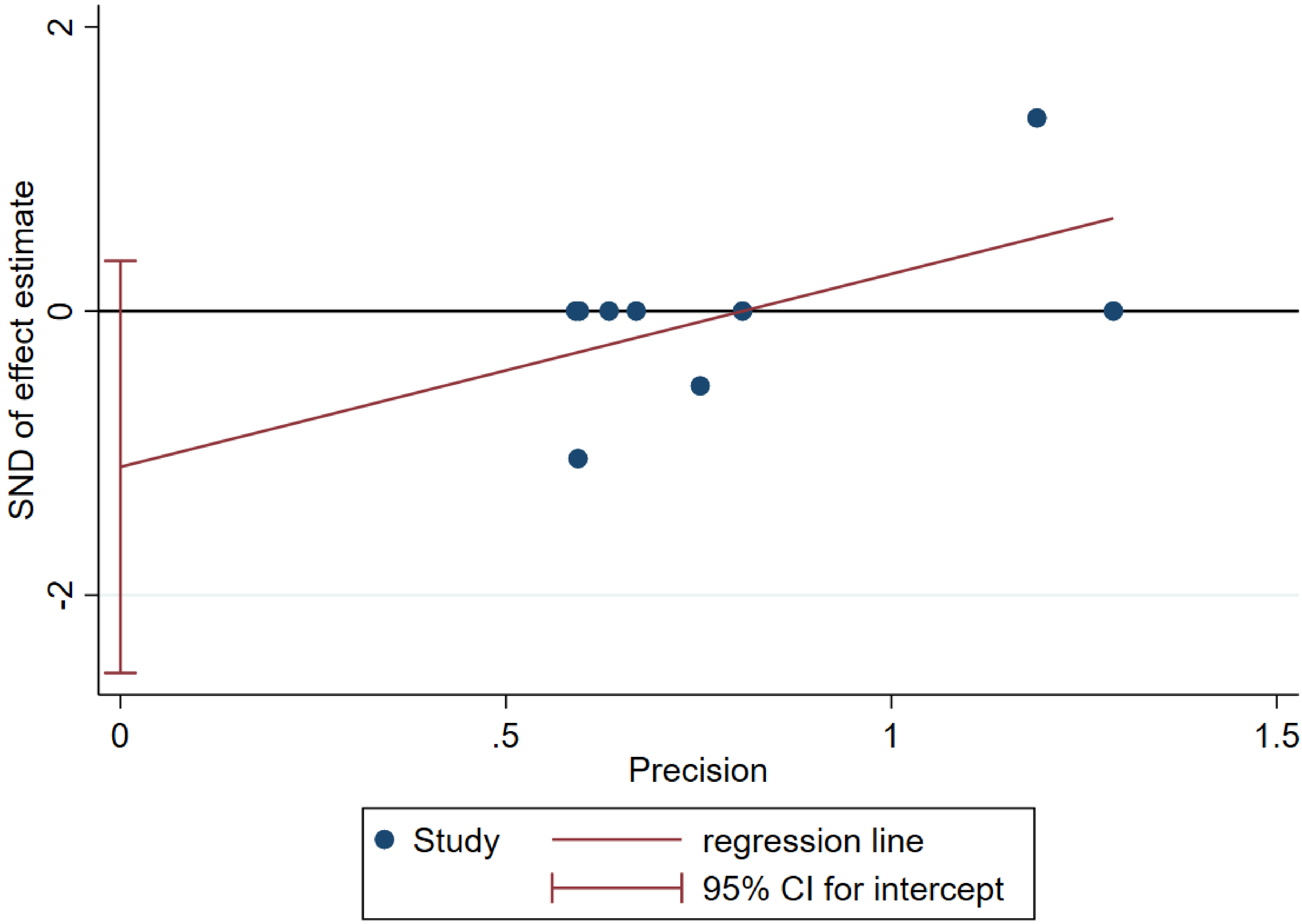

eFigure 5N Egger’s regression of acceptability in aspect of drop-out rate

Abbreviation for eFigure 5A-5N:

AD: Alzheimer's disease; ADAS-Cog: Alzheimer's disease assessment scale-cognitive subscale; a-tDCS-F3 + c-tDCS-F4: anodal tDCS of the left DLPFC and cathodal over the right DLPFC; a-tDCS-F3 + c-tDCS-Fp2: anodal tDCS of the left DLPFC and cathodal over right supraorbital region; a-tDCS-F3 + c-tDCS-RtLb: anodal tDCS of the left DLPFC and cathodal over the right deltoid muscle; a-tDCS-F7 + c-tDCS-Fp2: anodal tDCS of the left frontotemporal lobe and cathodal over right frontal lobe; a-tDCS-T3 + c-tDCS-Fp2: anodal tDCS of the left lateral temporal lobe and cathodal over right frontal lobe; a-tDCS-T3 + c-tDCS-RtLb: anodal tDCS of the left lateral temporal lobe and cathodal over right upper limb; a-tDCS-T3P3/T4P4 + c-tDCS-LtLb: anodal tDCS 2mA alternatively over the bilateral temporo-parietal lobe (T3-P3 or T4-P4) and cathodal over left arm deltoid muscle; CDR: clinical dementia rating; CI: confidence interval; c-tDCS-F3 + a-tDCS-Fp2: cathodal tDCS of the left DLPFC and anodal over right supraorbital region; DLPFC: dorsolateral prefrontal cortex; dTMS: deep TMS; HF-rTMS: high-frequency rTMS; HF-rTMS-F3: high frequency rTMS over left DLPFC; HF-rTMS-F3F4: high frequency rTMS over bilateral DLPFC; HF-rTMS-F3T3: high frequency rTMS over left DLPFC and left lateral temporal lobe; HF-rTMS-F4: high frequency rTMS over right DLPFC; HF-rTMS-Mx: high frequency rTMS multifocal stimulation; IQR: interquartile range; LF-rTMS: low-frequency rTMS; LF-rTMS-F3F4: low frequency rTMS over bilateral DLPFC; MD: mean difference; MMSE: mini-mental state examination; NIBS: noninvasive brain stimulation; NMA: network meta-analysis; OR: odds ratio; PRISMA: preferred reporting items for systematic reviews and the meta-analysis; RCT: randomized controlled trial; rTMS: repetitive transcranial magnetic stimulation; Sham: sham control; SMD: standardized mean difference; SUCRA: surface under the cumulative ranking curve; TBS: theta-burst stimulation; tDCS: transcranial direct current stimulation
